# CD97/*ADGRE5* attenuates the induction of adaptive type 2 immune responses in allergic asthma

**DOI:** 10.1038/s41467-026-76948-9

**Published:** 2026-08-28

**Authors:** Gabriela Aust, Anna-Lena Hoh, Jehan Alladina, Neal P. Smith, Florian Höhna, Bingyu Wang, Christiane Kerner, Marita Wagner, Knut Krohn, Arkadiusz Pierzchalski, Nele Häussler, Alexandra Chloe Villani, Josalyn L. Cho, Benjamin D. Medoff, Ana C. Zenclussen, Matthias Steinert, Jörg Hamann, Marianne Quaas, Tobias Polte

**Affiliations:** 1 Research Laboratories of the Department of Visceral, Transplantation, Vascular, and Thoracic Surgery, Leipzig University and University Hospital Leipzig, Leipzig, Germany; 2 Research Laboratories of the Department of Orthopaedics, Trauma, and Plastic Surgery, Leipzig University and University Hospital Leipzig, Leipzig, Germany; 3 Division of Pulmonary and Critical Care Medicine, Massachusetts General Hospital and Harvard Medical School, Boston, MA USA; 4 Center for Immunology and Inflammatory Diseases, Massachusetts General Hospital and Harvard Medical School, Boston, MA USA; 5 Department of Environmental Immunology, Helmholtz Centre for Environmental Research (UFZ), Leipzig, Germany; 6 Department of Dermatology, Venerology and Allergology, Leipzig University and University Hospital Leipzig, Leipzig, Germany; 7 Core Unit DNA-Technologies, Medical Faculty, Leipzig University, Leipzig, Germany; 8 Division of Pulmonary, Critical Care and Occupational Medicine, University of Iowa Carver College of Medicine, Iowa City, IA USA; 9 German Centre for Child and Adolescent Health (DZKJ), Leipzig, Germany; 10 Department of Experimental Immunology, Amsterdam Institute for Immunology and Infectious Diseases, Amsterdam University Medical Center, Amsterdam, The Netherlands; 11 Institute of Pathology, Martin Luther University Halle-Wittenberg, Halle, Germany

**Keywords:** Asthma, Experimental models of disease, Conventional dendritic cells, Mucosal immunology, T-helper 2 cells

## Abstract

Allergic asthma results from an uncontrolled type 2 immune response to inhaled allergens. Here, we investigate the function of CD97/*ADGRE5*, expressed in mouse and human immune and lung epithelial cells, in this disease. Female *Cd97*^*−/−*^ mice exhibit an exacerbated asthmatic phenotype across multiple models, primarily due to CD97 loss on immune cells. A single CD97 antibody treatment before allergen sensitization worsens allergic responses, highlighting a role for CD97 in early immune regulation. Post-sensitization, *Cd97*^*−/−*^ mice display higher frequencies of lung conventional type 2 and monocyte-derived dendritic cells (DCs). Allergen-pulsed *Cd97*^*−/−*^ bone marrow-derived DCs are more activated, promote enhanced proliferation and type 2 cytokine secretion by CD4⁺ OT-II cells, and induce stronger airway inflammation. Consistently, *ADGRE5* expression is reduced in airway mucosa-derived mononuclear phagocyte subsets in human asthmatics after allergen-induced exacerbation. These results identify CD97 as an important regulator of DC-driven type 2 allergic responses and a potential target in asthma.

## Abstract (web summary)

Allergic asthma involves different lung cells and mediators. Here, the authors examine the function of CD97/*ADGRE5* and show that its absence increases the severity of allergic lung inflammation in mice. Allergen-pulsed *Cd97*^*-/-*^ DCs are more activated and promote stronger type 2 immune responses than WT DCs and, in humans, lung DCs from allergen-exposed asthmatics express less CD97 than allergic non-asthmatics.

## Introduction

Allergic asthma is a common chronic disease that affects more than 340 million people worldwide (globalasthmareport.org)^1^. It encompasses a complex and heterogeneous disease spectrum with mild to severe disease phenotypes, characterized by airway inflammation, reversible airway hyper-responsiveness (AHR), elevated serum IgE levels, and airway remodelling with increased subepithelial fibrosis and smooth muscle mass^1^.

Allergic asthma is considered to be an inappropriate type 2 immune response to environmental allergens such as house dust mite (HDM) in susceptible individuals. This response involves T helper 2 (Th2) cells, T follicular helper 2 cells (Tfh2), and innate lymphoid cells type 2 (ILC2) producing type 2 cytokines such as IL-4, IL-5, and IL-13^2^. The initiation of a specific allergic response depends on signals delivered by lung dendritic cells (DCs), which capture and process the allergen, migrate to mediastinal lymph nodes (mLNs), and activate naïve CD4^+^ T cells, leading to their polarization. In the asthmatic lung, Th2-mediated immunity is established and maintained by CD11b^+^ DCs, consisting of conventional type 2 (cDC2s) and monocyte-derived (moDC) DCs, with cDC2s being potent inducers of Tfh2 cells^3–5^. Th2 cells are crucial for eosinophilic airway inflammation and AHR via the release of type 2 cytokines, while Tfh2 cells activate B cells to produce antigen-specific IgE^6^.

In addition to Th2/Tfh2-driven specific immunity, the innate immune response and the interaction between the two play an important role in allergic asthma. The airway epithelium is the first line of defence against pathogenic environmental triggers and releases alarmins, pro-inflammatory cytokines such as thymic stromal lymphopoietin (TSLP), IL-25, and IL-33 that attract and/or activate DCs, ILC2s and other immune cells^7,8^. While the immunological mechanisms underlying allergic asthma are relatively well characterized, the specific mechanisms that modulate type 2 allergic inflammatory responses in the lung are poorly understood.

The adhesion G protein-coupled receptor (GPCR) CD97, encoded by *ADGRE5*, is highly expressed on immune cells and, to a lesser extent, on various epithelial cells, particularly in the lung^9,10^. CD97 features an extended extracellular N-terminal fragment (NTF) that mediates adhesion to interaction partners such as CD55^11^ and THY1/CD90^12^. The NTF is associated with the C-terminal fragment, that comprises a short extracellular region containing the tethered intramolecular agonist (TIA), critical for receptor activation^13,14^, the seven-transmembrane domain, and the intracellular tail. TIA exposure, f. e. via shedding the NTF by CD55^15^, activates Gα13-Rho GTPase signaling leading to actin cytoskeleton alterations and enhanced migration^13,14,16^.

Remarkably, the cellular distribution and structure of CD97 are similar in human and mouse, suggesting a conserved biological role. Therefore, *Cd97*^−/−^ mice^9,16,17^ and anti-mouse CD97 antibodies (Abs) directed to the EGF-like folds in the NTF^9,18^ have been used to investigate the role of CD97 in mouse models of innate and adaptive immunity. Overall these results suggest that CD97 plays an important role, particularly in models of innate immunity^9^. However, it remains to be elucidated whether this also applies to allergic asthma, in which both innate and adaptive immunity are involved.

Our study aimed to investigate the role of CD97 in allergic airway inflammation using (conditional) *Cd97*^−/−^ mice in both ovalbumin (OVA)- and/or HDM-induced acute and chronic asthma models. We administered an CD97 Ab, selectively accumulating in the lung^19^, before and during asthma induction to assess the function of CD97 in sensitization and maintaining the allergic response. Lastly, we analysed *ADGRE5* expression using single-cell transcriptomic data from lower-airway mucosal cells obtained from allergic asthmatics and allergic non-asthmatics.

Our data show that loss of CD97/*ADGRE5* exacerbates allergic asthma in mice, with cDC2s and moDCs playing a causal role during allergen challenge. Consistent with these findings, several subsets of lung mononuclear phagocytes (MNPs), including cDC2s, express lower levels of *ADGRE5* in allergen-challenged allergic asthmatics than in allergic non-asthmatics.

## Results

### Loss of CD97 leads to an exacerbation of allergic asthma

**Fig. 1:**
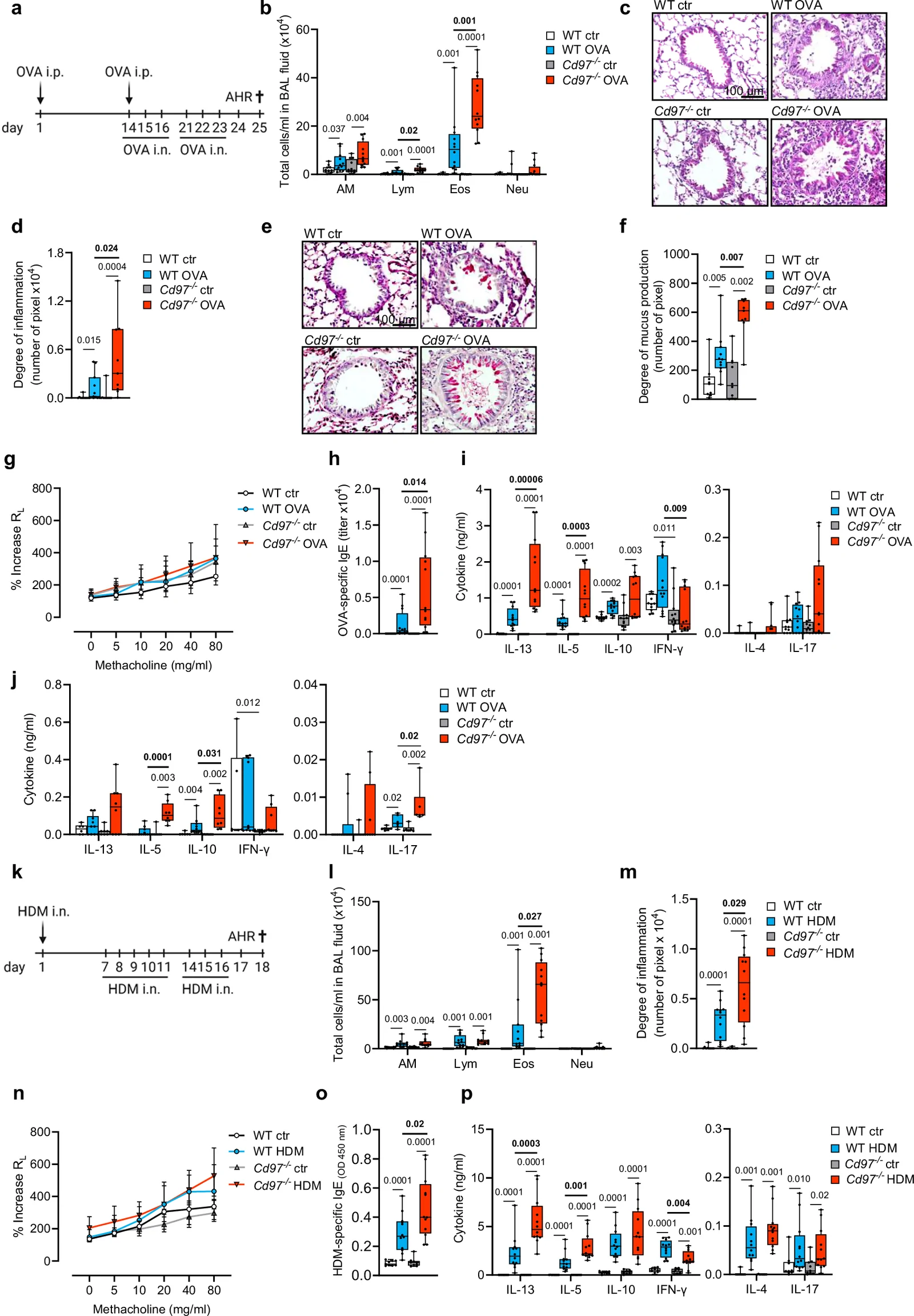
Loss of CD97 leads to an exacerbated allergic asthma. **a** Using an OVA-induced asthma model (airway hyperresponsivess, AHR) **b** total numbers of cells in BAL fluid, **c** inflammation of lung tissues (HE stained lung sections), **d** quantified by computer-based image-analysis, **e** mucus production (PAS-stained lung sections), **f** quantified by computer-based image-analysis, **g** lung resistance (AHR), **h** OVA-specific serum IgE and cytokine levels in **i** restimulated splenocytes and **j** mLN were determined in WT and *Cd97*^*−/−*^ mice. **k** In an adjuvant-free HDM-induced asthma model **l** total cell count in bronchoalveolar lavage (BAL) fluid, **m** inflammation of lung tissues, **n** lung resistance, **o** HDM-specific serum IgE, and **p** cytokine levels in restimulated splenocytes were determined in WT and *Cd97*^*−/−*^ mice. (**b**, **g**, **h**, **i**) WT ctr: *n* = 11; WT OVA, *Cd97*^*−/−*^ ctr, *Cd97*^*−/−*^ OVA: *n* = 12 (**d**, **f**) WT ctr, *Cd97*^*−/−*^ OVA: *n* = 9; WT OVA: *n* = 11; *Cd97*^*−/−*^ ctr: *n* = 10 (**j**) WT ctr: *n* = 6; WT OVA: *n* = 10; *Cd97*^*−/−*^ ctr, *Cd97*^*−/−*^ OVA: *n* = 8 (**l**–**p**) ctr: *n* = 9; HDM: *n* = 12 mice/group. The data shown are from 3 independent experiments. Data are presented as box-and-whisker plots, each data point represents an individual mouse, the centre line indicates the median, the box represents the interquartile range (IQR), and whiskers extend from minimum to the maximum. Lung resistance data (**g**, **n**) are presented as median with IQR. For statistical analysis, 2-sided Mann–Whitney *U* test was used. i.p. intraperitoneal, i.n. intranasal, AM alveolar macrophages, Lym lymphocytes, Neu neutrophils, Eos eosinophils.

The role of CD97 in allergic asthma was studied using *Cd97*^*−/−*^ and WT mice in acute and chronic asthma models^20,21^. In an OVA-induced model (Fig. 1a), *Cd97*^*−/−*^ mice exhibited increased eosinophilic inflammation, airway mucus production, allergen-specific IgE levels, and elevated type 2 cytokines (IL-5, IL-13), whereas IFN-γ was reduced. AHR was unaffected, as were IL-17 levels (Fig. 1b–j).

Similar findings were observed in an adjuvant-free acute asthma model with pulmonary sensitization to the clinically relevant allergen HDM (Fig. 1k), including enhanced eosinophilic inflammation, elevated IgE and splenic type 2 cytokines, and no pronounced effects on AHR (Fig. 1l–p).

Finally, chronic asthma studies demonstrated an increased number of neutrophils in the BAL fluid, elevated lung inflammation and lung resistance, higher splenic IL-5 and IL-13 levels, and increased lung smooth muscle mass in *Cd97*^*−/−*^ compared to WT mice (Supplementary Fig. 1a–i), confirming in detail that CD97 deficiency exacerbates allergic asthma regardless of the allergen or model used.

### Depletion of CD97 in leukocytes, but not in lung epithelial cells, increases allergic asthma

**Fig. 2:**
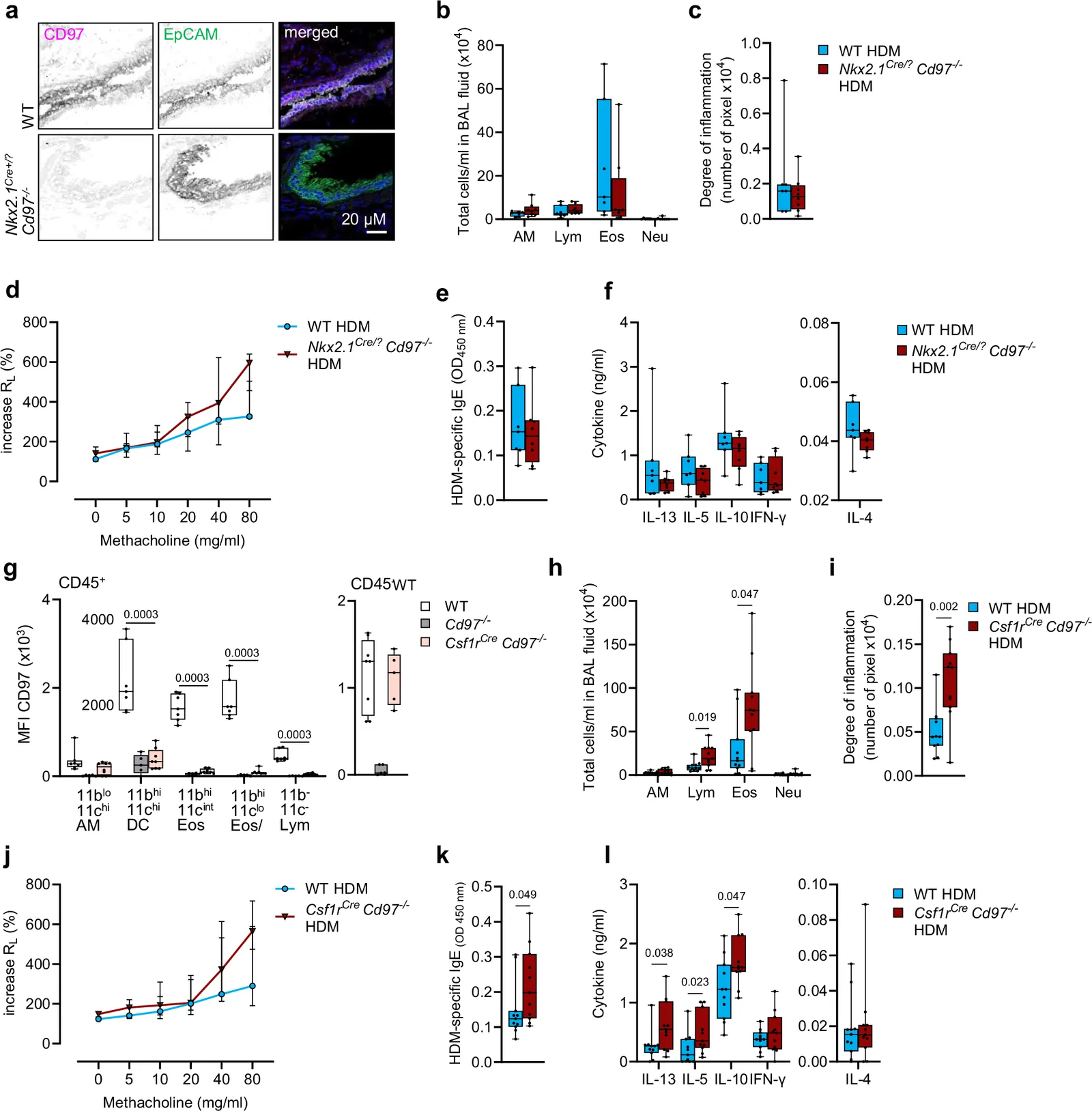
Depletion of CD97 in leukocytes, but not in lung epithelial cells, exacerbates allergic asthma phenotype. **a** Characterization of *Nkx2.1*^*Cre/?*^ *Cd97*^*−/−*^ in comparison to WT mice by lung immunohistology. CD97 was lost in bronchial epithelial cells (EpCAM^+^) of the *Nkx2.1*^*Cre/?*^ *Cd97*^*−/−*^ mouse. In the HDM-induced asthma model (see Fig. 1k), the asthma phenotype of WT and *Nkx2.1*^*Cre/?*^ *Cd97*^*−/−*^ mice was assessed by **b** cell numbers in the BAL fluid, **c** lung inflammation, **d** lung resistance, **e** HDM-specific IgE, and **f** cytokine production in restimulated splenocytes. **g** CD97 expression was quantified in lung leukocytes (CD45^+^) and CD45^-^ non-immune cells of WT, *Cd97*^*−/−*^, and *Csf1r*^*Cre/?*^ *Cd97*^*−/−*^ mice by flow cytometry. For the gating strategy, subtyping of leukocytes by CD11b/CD11c and subset designation see Supplementary Fig. 2c. In the HDM-induced asthma model, the asthma phenotype of WT and *Csf1r*^*Cre/?*^ *Cd97*^*−/−*^ mice was assessed by **h** cell numbers in BAL fluid, **i** lung inflammation, **j** lung resistance, **k** HDM-specific IgE, and **l** cytokine production in the spleen. **a** Representative micrographs from 3 independent experiments, (**b**–**f**) WT HDM: *n* = 7, *Nkx2.1*^*Cre/?*^ *Cd97*^*−/−*^ HDM: *n* = 8 (g) *Cd97*^*−/−*^: *n* = 5, WT: *n* = 6, *Csf1r*^*Cre/?*^ *Cd97*^*−/−*^ CD45^+^: *n* = 8, CD45^-^: *n* = 5; (**h**–**l**) *n* = 11 mice/group. Data are presented as box-and-whisker plots, each data point represents an individual mouse, the centre line indicates the median, the box represents the IQR, and whiskers extend from minimum to the maximum. Lung resistance data (**d**, **j**) are presented as median with IQR. For statistical analysis, 2-sided Mann–Whitney *U* test was used.

CD97/*ADGRE5* expression is not restricted to hematopoietic cells in mice^9^ and humans^10^. To identify the CD97-positive lung cell type critical for its asthma-attenuating effects, mice with cell type-specific CD97 loss were generated (Supplementary Fig. 2a, b). In *Nkx2.1*^*Cre/?*^ *Cd97*^*−/−*^ mice, CD97 was absent in bronchial epithelial cells (Fig. 2a) but remained active in various immune cell subsets (Supplementary Fig. 2c, d). These mice showed an asthma phenotype comparable to WT mice in the acute HDM-induced model (Fig. 2b–f). We further quantified TSLP and IL-33, as well as ILC2s in the lungs of WT and *Cd97*^*−/−*^ mice (Supplementary Fig. 3). We confirmed that CD97 expression in the airway epithelium and in ILC2s were not the primary mediators of CD97’s asthma-attenuating effect, because alarmin levels were similar in both genotypes (Supplementary Fig. 3a, b), and only WT mice showed a higher frequency of ILC2s among leukocytes post-sensitization (Supplementary Fig. 3c–f). Instead, *Csf1r*^*Cre/?*^ *Cd97*^*−/−*^ mice in which CD97 was absent in leukocytes but present in non-immune cells (Fig. 2g) showed an enhanced asthma phenotype (Fig. 2h–l).

### Loss of *Cd55* has no effect on allergic asthma

To investigate the role of the well-characterized CD97 interaction partner CD55/DAF^11^ in the development of an allergic immune response, we used *Cd55*^*−/−*^ mice, previously named *Daf1*^*−/−*^^22^, in the HDM-induced asthma model. Loss of *Cd55* had no apparent effect on asthma development compared to WT mice (Supplementary Fig. 4a–e). In *Cd55*^*−/−*^ mice, alveolar macrophages, eosinophils/neutrophils and lymphocytes were nearly CD55^-^, but CD55 was still present in CD11b^hi^ CD11c^hi^ cells, mainly DCs (Supplementary Fig. 4f). This is attributable to the second gene encoding mouse CD55, *Cd55b*, which remains intact in *Cd55*^*−/−*^ mice^22^. Furthermore, CD97 was higher in isolated lung leukocyte subsets (alveolar macrophages are CD97^-^) and non-immune cells of *Cd55*^*−/−*^ *vs*. WT mice (Supplementary Fig. 4g).

### CD97 Ab treatment before HDM sensitization exacerbates allergic asthma

**Fig. 3:**
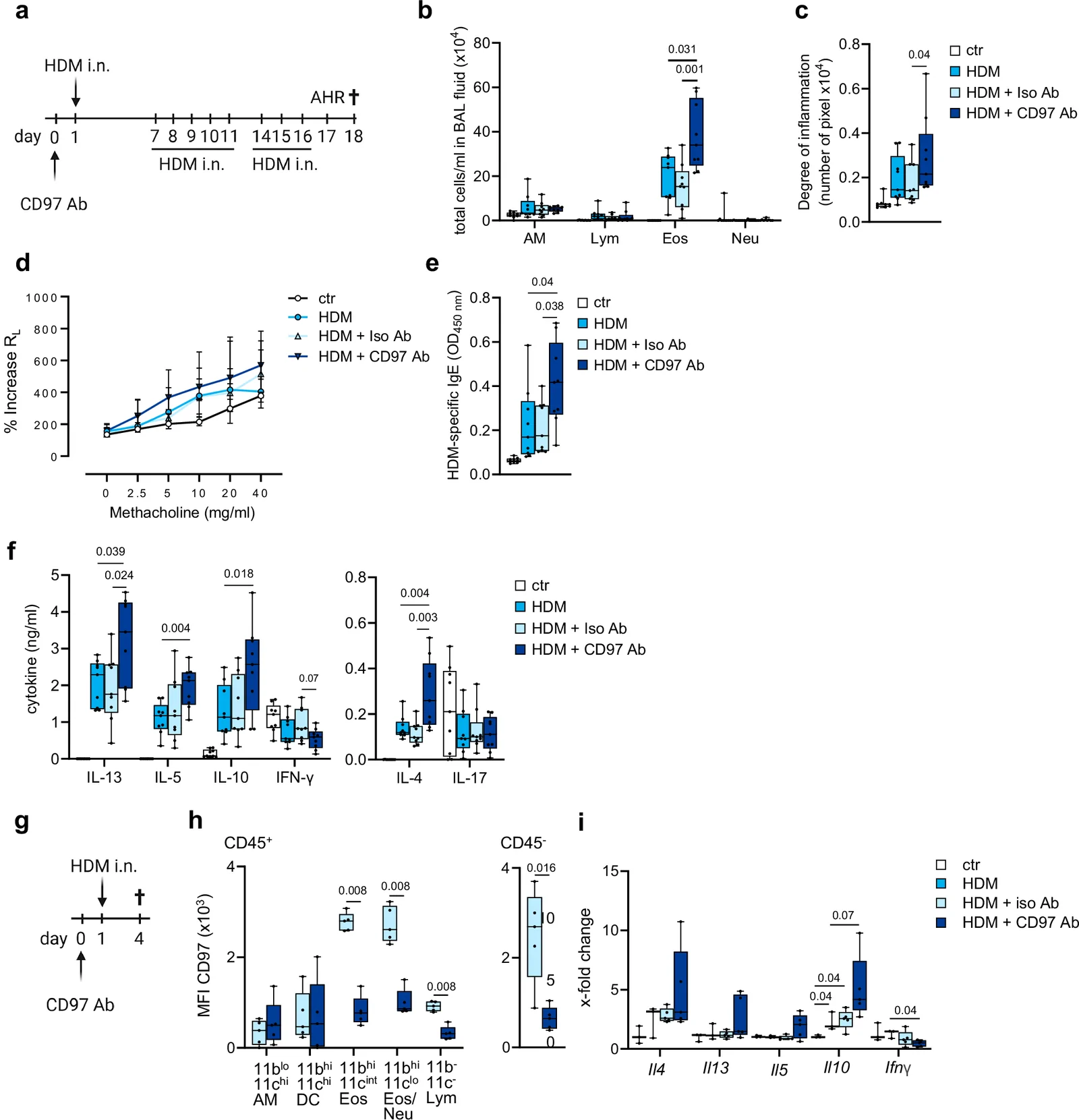
Single CD97 Ab application before allergen sensitization leads to an increased asthma phenotype in HDM-sensitized mice. **a** The Abs were applied one day before HDM sensitization. **b** The number of cells in BAL fluid, **c** inflammation of lung tissues, **d** lung resistance, **e** HDM-specific IgE, and **f** cytokine production in the spleen were assessed in CD97 Ab- and isotype Ab-treated BALB/c mice. **g** In a short-term protocol the Abs were applied one day before HDM sensitization, **h** CD97 expression was quantified in lung leukocytes (CD45^+^) and CD45^−^ non-immune cells 72 h after HDM sensitization by flow cytometry. Gating strategy and subtyping of leukocyte subsets see Supplementary Fig. 2c. **i** qRT-PCR of Th1- and Th2-related cytokines of lung leukocytes. CD45^+^ cells were enriched from isolated lung cells by magnetic cell separation and afterwards restimulated with 50 µg/ml HDM for 6 h. **b**–**f** *n* = 9 (**h**) *n* = 5 (**i**) ctr, HDM: *n* = 3; HDM + iso Ab, HDM + CD97 Ab: *n* = 5 mice/group, 2 technical replicates/mouse. Data are presented as box-and-whisker plots, each data point represents an individual mouse, the centre line indicates the median, the box represents the IQR, and whiskers extend from minimum to the maximum. Lung resistance data **d** are presented as median with IQR. For statistical analysis, 2-sided Mann–Whitney *U* test was used.

To further elucidate the role of CD97 in allergic asthma, we used the CD97 Ab 1B2^19^, which inhibits the neutrophil migration in models of innate immunity^23^, and compared it with an isotype control Ab during HDM-induced asthma in BALB/c mice. Interestingly, a single CD97 Ab application before allergen sensitization increased the asthma phenotype (Fig. 3a–f), similar to the protocol using three Ab applications, once before sensitization and twice during allergen challenge (Supplementary Fig. 5a–f), and to Cd97^−/−^ mice. In contrast, CD97 Ab treatment during antigen challenge to investigate the role of CD97 in the maintenance of allergic airway inflammation, including inflammatory cell migration into the lung, did not result in differences compared to the isotype control Ab-treated mice (Supplementary Fig. 5g–l).

Further analysis showed that the CD97 Ab reduced CD97 expression on both leukocytes and non-immune cells in the lung 72 h after HDM sensitization (Fig. 3g, h), ultimately mimicking the *Cd97*^*−/−*^ mouse. This was accompanied by an altered cytokine mRNA profile in enriched lung CD45^+^ cells (Fig. 3i).

### Early increase in the frequency and activation of lung cDC2s and moDCs of HDM-treated *Cd97*^*−/−*^ mice

Notably, a single CD97 Ab treatment before allergen sensitization exacerbated allergic asthma, highlighting a role for CD97 in early immune regulation. As lung epithelial cell-derived alarmins and ILC2s were excluded as mediators of this CD97-attenuating effect, DCs became the focus of our study.

**Fig. 4:**
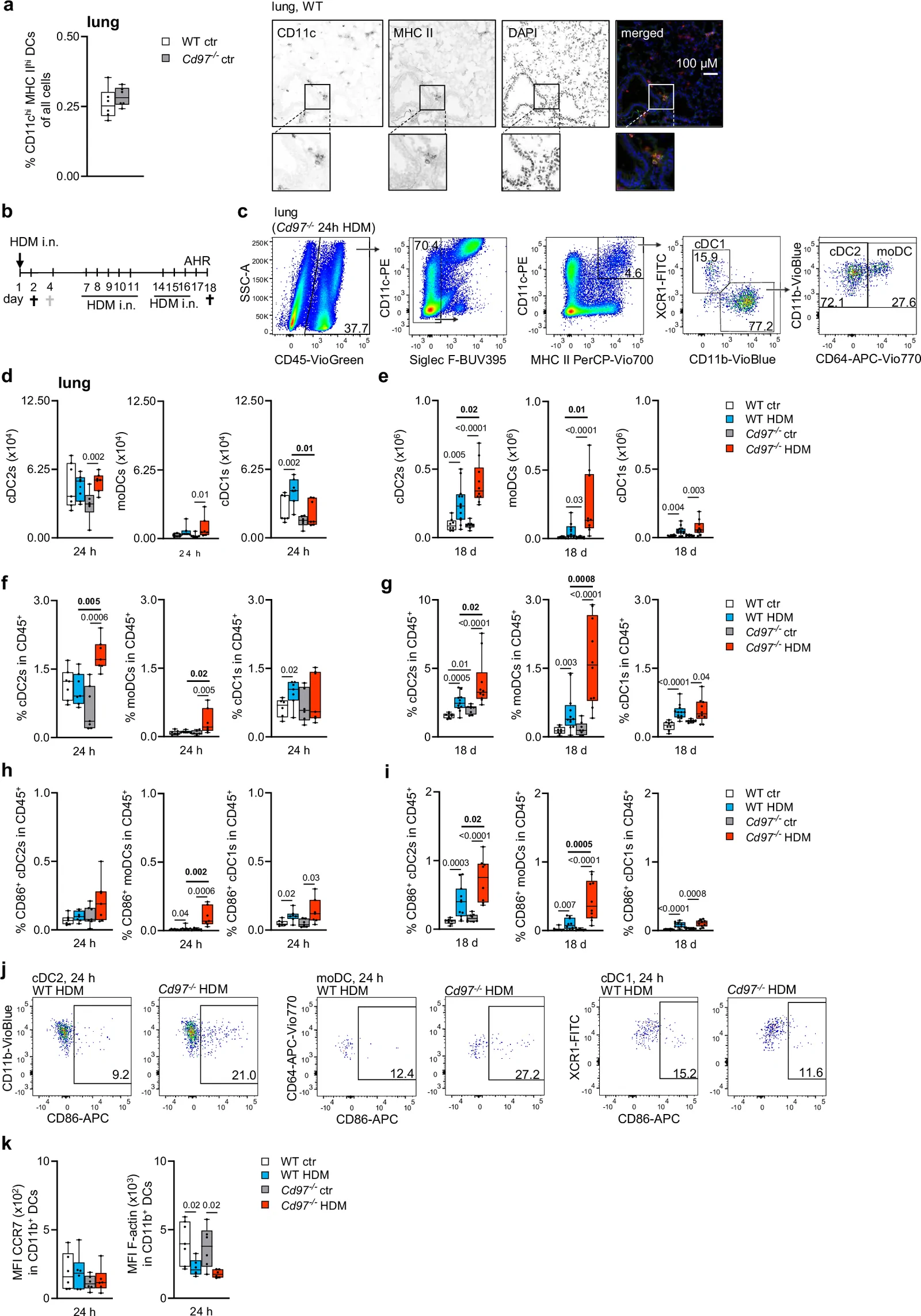
Increased frequencies of lung cDC2s and (activated) moDCs post- sensitization in *Cd97*^*−/−*^ mice. **a** Absolute numbers of CD11c^hi^ MHC II^hi^ DCs in lung cryosections from naïve WT and *Cd97*^*−/−*^ mice. CD11c^hi^ leukocytes were evenly distributed throughout the lung, and only a few co-expressed high levels of MHC II (MHC II^hi^). Left: Frequency of CD11^hi^ MHCII^hi^ among all DAPI^+^ cells, quantified in two non-serial lung sections per mouse (*n* = 6 mice/genotype); Right: Representative immune-stained lung section (inserts show detail). **b** Protocol: isolated lung cells were analysed by flow cytometry 24 h post-sensitization and on day 18 of the acute HDM model for the presence of cDC2s, moDCs, and cDC1s. **c** gating strategy, representative example: cDC2s and moDCs are characterized as CD45^+^ CD11c^hi^ MHC II^hi^ Siglec F^-^ CD11b^+^ XCR1^−^. cDC2s are CD64^-^, whereas moDCs are CD64^+^. cDC1s are CD11b^-^ XCR1^+^. **d**, **e** Absolute numbers cDC2s, moDCs, and cDC1s. **f**, **g** Frequencies of cDC2s, moDCs, and cDC1s and of **h**, **i** activated CD86^+^ cDC2s, moDCs, and cDC1s among lung leukocytes. **j** Flow cytometry dot blots of a representative HDM-treated WT and *Cd97*^*−/−*^ mouse 24 h post-sensitization; pregated according to the gating strategy in **c**; the frequency of CD86^+^ cells are indicated in the quadrants. **k** CCR7 and F-actin MFI of lung CD11b^+^ DCs, consisting of cDC2s and moDCs, 24 h post-sensitization. **a** *n* = 6; (**d**, **f**, **h**) *n* = 7; (**e**, **g**, **i**) ctr: *n* = 8, WT HDM: *n* = 12, *Cd97*^*−/−*^ HDM: *n* = 10; (**k**) *n* = 6 mice/group. Data are presented as box-and-whisker plots, each data point represents an individual mouse, the centre line indicates the median, the box represents the IQR, and whiskers extend from minimum to the maximum. For statistical analysis, 2-sided Mann–Whitney *U* test was used.

In naïve mice of both genotypes, the absolute numbers of lung CD11c^hi^ MHC II^hi^ DCs (comprising cCD2s, moDCs, and cDC1s^24^), assessed by immunohistology, were similar (Fig. 4a). Likewise, the frequencies and activation status across these lung DC subsets, as well as CCR7 and F-actin levels in CD11b^+^ DCs, were similar between genotypes (Fig. 4b–k). In the mLNs resident and migratory DCs were examined. As expected, migratory DCs expressed higher CCR7 and F-actin levels (Supplementary Fig. 6a, b). Consistent with the lung, the frequencies of (migratory and resident) mLN cDC2s and moDCs were also comparable in naïve mice (Supplementary Fig. 6c, d). In contrast, splenic cDC2 frequencies were already reduced in naïve *Cd97*^*−/−*^ compared with WT mice (Supplementary Fig. 6e), confirming recent findings^16^.

In HDM-treated mice, cDC2s and moDCs were predominantly affected in the lungs and, to lesser extent, in the mLNs. After sensitization, the absolute numbers and the frequencies of cDC2s and moDCs, as well as activated moDCs, increased only in the lung of *Cd97*^*−/−*^ mice (Fig. 4d, f, h, j). The absolute numbers of lung cDC1s, promoting Th1 polarization, increased less in *Cd97*^*−/−*^ than in WT mice (Fig. 4d).

By day 18, the elevated absolute numbers and frequencies of (activated) cDC2s and moDCs were significantly higher in *Cd97*^*−/−*^ than in WT mice (Fig. 4e, g, i). In the mLNs, obvious changes were observed only in migratory DCs post-sensitization. *Cd97*^*−/−*^ mice showed higher frequencies of moDCs and activated moDCs compared with WT mice (Supplementary Fig. 6c, d).

### CD97 deficiency affects the function of bone marrow (BM)-derived DCs

Due to the low abundance and high heterogeneity of lung DCs^24^, and the difficulty of isolating sufficient DCs for in vitro and in vivo studies, we used BMDCs as a surrogate model to assess the impact of CD97 deficiency on DC function.

**Fig. 5:**
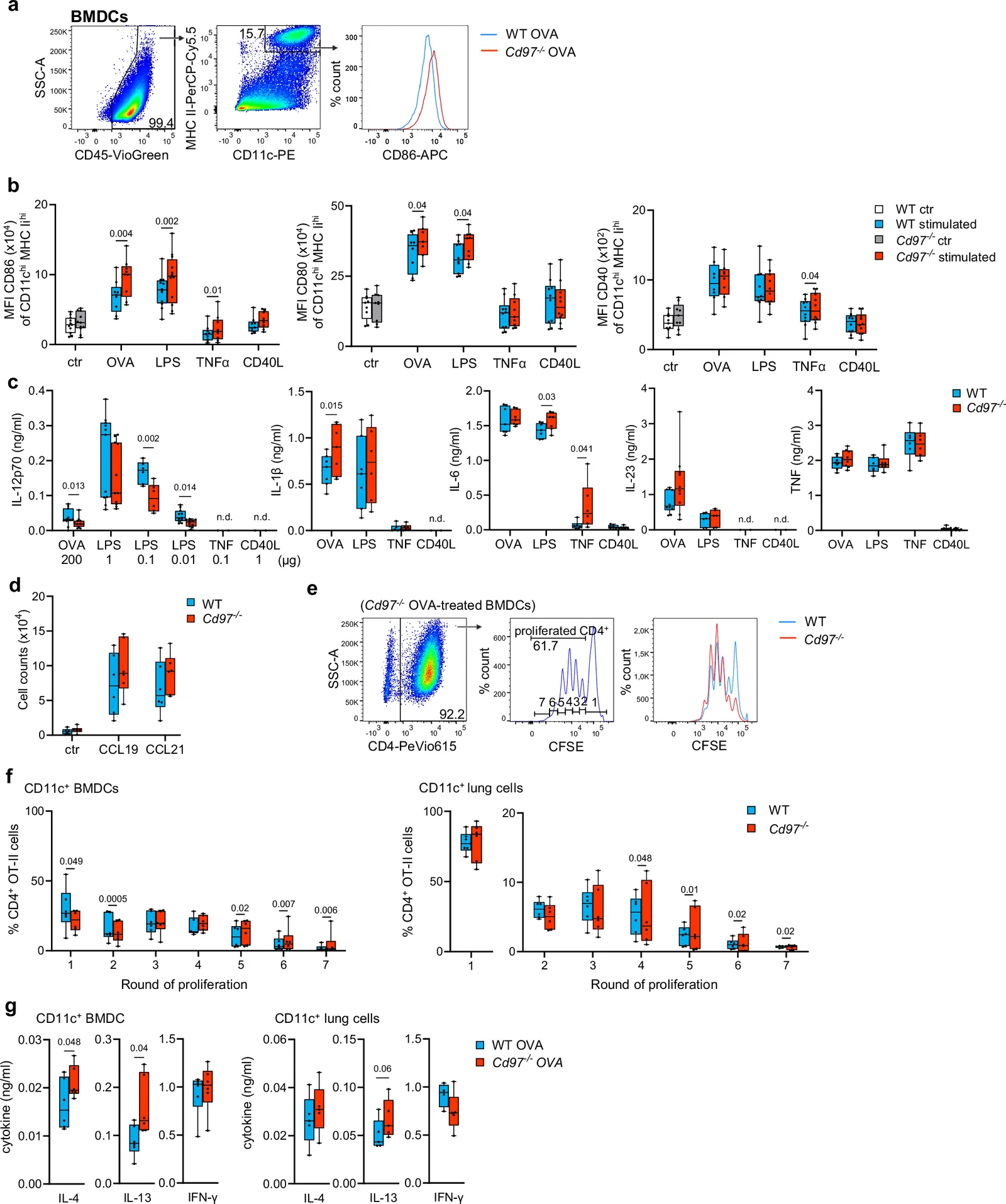
Absence of CD97 affects the function of bone-marrow (BM)-derived DCs. **a**–**c** BMDCs were generated in parallel from one WT and one *Cd97*^*−/−*^ mouse, stimulated with the indicated substances, and analysed for activation by flow cytometry. Cytokines were quantified in the corresponding cell culture supernatants. **a** Flow cytometry gating strategy: CD45^+^ cells were gated for CD11c^hi^ MHC II^hi^ BMDCs; the median fluorescence intensity (MFI) of activation markers was quantified. Right panel: Representative overlay of CD86 histograms from one experiment. **b** Expression of activation markers (CD86, CD80, CD40L) in OVA-, LPS-, TNF-, and CD40-stimulated BMDCs. **c** Quantitation of cytokines (IL-12p70, IL-1β, IL-6, IL-10, IL-23) in the BMDC supernatants 24 h after stimulation; n.d., not detected. **d** Quantitation of BMDC migration toward CCL19 and CCL21 through a transwell chamber. **e**–**g** In vitro proliferation of splenic CD4^+^ OT-II cells analysed by flow cytometry. Enriched CD4^+^ OT-II cells were labeled with CFSE and co-cultured with OVA-pulsed 1 × 10^4^ CD11c^+^ BMDCs or 2 × 10^4^ CD11c^+^ lung cells from one mouse/genotype for 72 h. **e** CD4^+^ cells were analysed for CFSE expression by flow cytometry; analysis strategy. Individual CFSE peaks were gated in histograms and quantified. Right: Representative overlay of CFSE histograms of one experiment. **f** The percentage of cells that had accomplished 1, 2, 3… rounds of proliferation are shown. **g** Quantitation of cytokines (IL-4, IL-13, IFN-γ) in the co-culture supernatants. **b** CD86: *n* = 9, CD80: *n* = 7–9, CD40: *n* = 8–9 mice/group; (**c**,**d**) *n* = 7 mice/group; **f** *n* = 6 experiments; **g** *n* = 7 experiments (each experiment included one mouse/genotype). Data are presented as box-and-whisker plots, each data point represents an individual mouse, the centre line indicates the median, the box represents the IQR, and whiskers extend from minimum to the maximum. For statistical analysis, two-sided Mann–Whitney *U* test (**b**–**d**) and two-sided paired Wilcoxon test (**f**, **g**) were used.

Activated DCs orchestrate type 1, type 2, and Th17 immune responses, guided by cytokines. IL-12 drives type 1 polarization, whereas IL-1β, IL-6 and IL-23 promote Th17 responses^25,26^. Upon OVA, lipopolysaccharide (LPS), and in part also TNF stimulation, *Cd97*^*−/−*^ BMDCs exhibited higher CD86 and CD80 expression and, importantly, produced less IL-12. OVA-stimulation elevated IL-1β levels, whereas LPS and TNF induced higher IL-6 secretion in *Cd97*^*−/−*^ than in WT BMDCs (Fig. 5a–c).

**Fig. 6:**
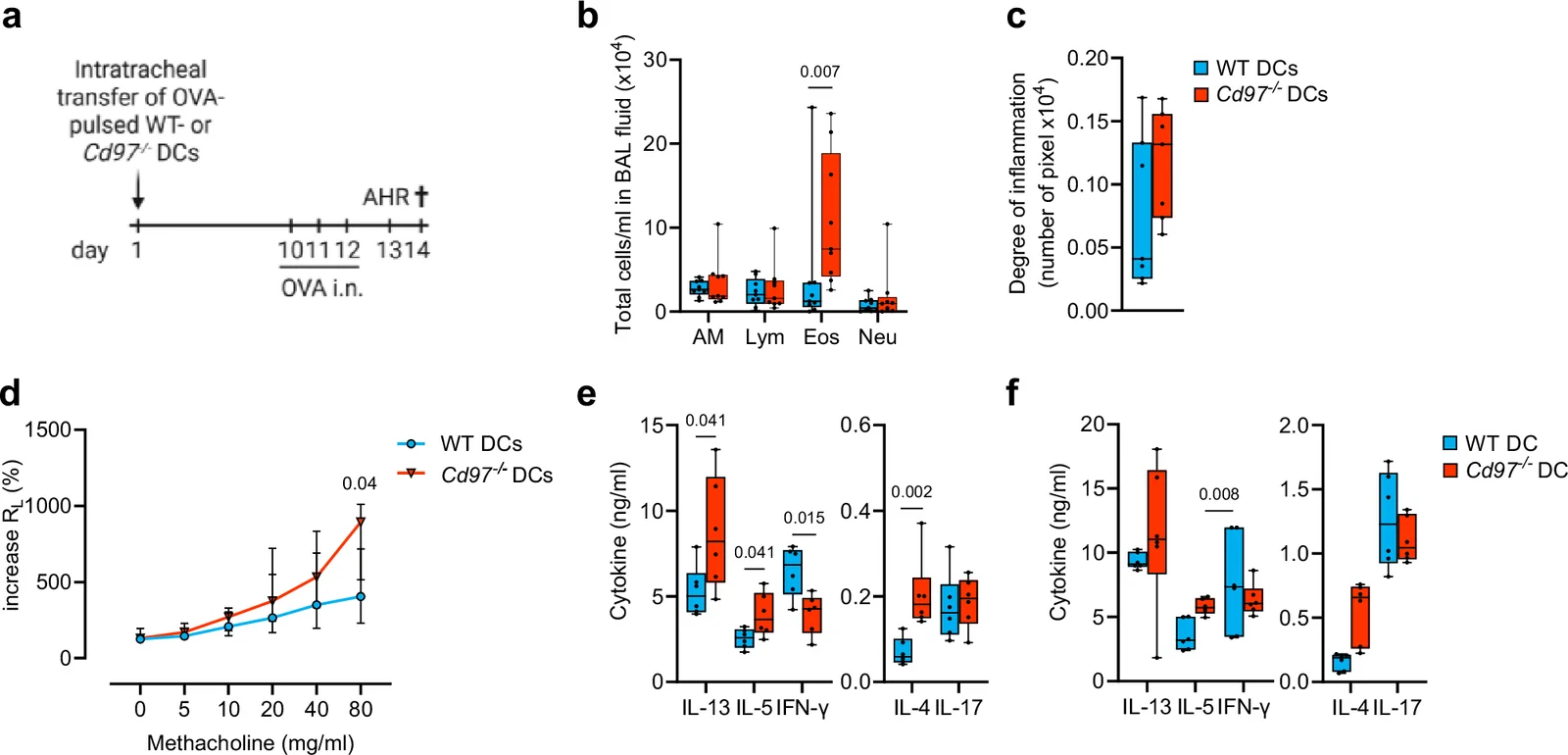
Intratracheal transfer of *Cd97*^*−/−*^ BMDCs worsened allergic asthma. **a**  Using an adoptive BMDC transfer protocol, **b** the number of cells in BAL fluid, **c** inflammation of lung tissues, **d** lung resistance, and Th2 cytokine levels in **e** restimulated splenocytes and **f** mLN cells were analysed in WT mice receiving OVA-pulsed DCs from *Cd97*^*−/−*^ mice compared to control mice. **b**–**d** *n* = 9; (**e**,**f**) *n* = 6 mice/genotype. Data are presented as box-and-whisker plots, each data point represents an individual mouse, the centre line indicates the median, the box represents the IQR, and whiskers extend from minimum to the maximum. Lung resistance data **d** are presented as median with IQR. For statistical analysis, two-sided Mann–Whitney *U* test was used.

Furthermore, the migration rate of LPS-stimulated *Cd97*^*−/−*^ BMDCs toward CCL19 and CCL21, chemokines that primarily bind to CCR7 and regulate immune cell homing to lymphoid tissues, was comparable to that of WT cells (Fig. 5d). To evaluate the impact of CD97 on DC-mediated T cell skewing, OVA-pulsed CD11c^+^ BMDCs or CD11c^+^ lung cells were co-cultured with CD4^+^ cells from OT-II mice carrying a transgenic OVA-recognising T cell receptor. CD11c^+^ BMDCs and CD11c^+^ lung cells from *Cd97*^*−/−*^ mice induced a stronger proliferation of CD4^+^ cells, and CD11c^+^ BMDCs also promoted a higher IL-13 release, compared to WT cells (Fig. 5e–g). Moreover, OVA-pulsed BMDCs were transferred into naïve BALB/c mice, which were subsequently treated with OVA (Fig. 6a). Mice receiving *Cd97*^*−/−*^ CD11c^+^ BMDCs exhibited increased eosinophilic inflammation, lung resistance, and enhanced splenic and mLN type 2 cytokine levels, while airway inflammation remained unchanged (Fig. 6b–f).

Notably, RNA-sequencing (RNA-seq) analysis of CD11c^+^ OVA-pulsed BMDCs revealed distinct transcriptional profiles between WT and *Cd97*^*−/−*^ DCs, with 153 genes differentially expressed between the two genotypes (Supplementary Data 1). Subsets of these genes (Supplementary Fig. 7) showed highly significant enrichment (DAVID enrichment score 3.48) in a gene set analysis to UniProt and Gene Ontology (GO) terms “signal” (KW-0732), “extracellular region” (GO:0005576), “secreted” (KW-0964), and “extracellular space” (GO:0005615). This indicates that CD97 deficiency primarily affects how DCs communicate by altering the secretome (cytokines and chemokines), cell–cell and cell–extracellular matrix interactions, and paracrine signalling, thereby ultimately shaping local immune responses.

### Early increase of lung and mLN GATA3^+^ in CD4^+^ T cells and early activation of lung T cells in HDM-sensitized *Cd97*^*−/−*^ mice

**Fig. 7:**
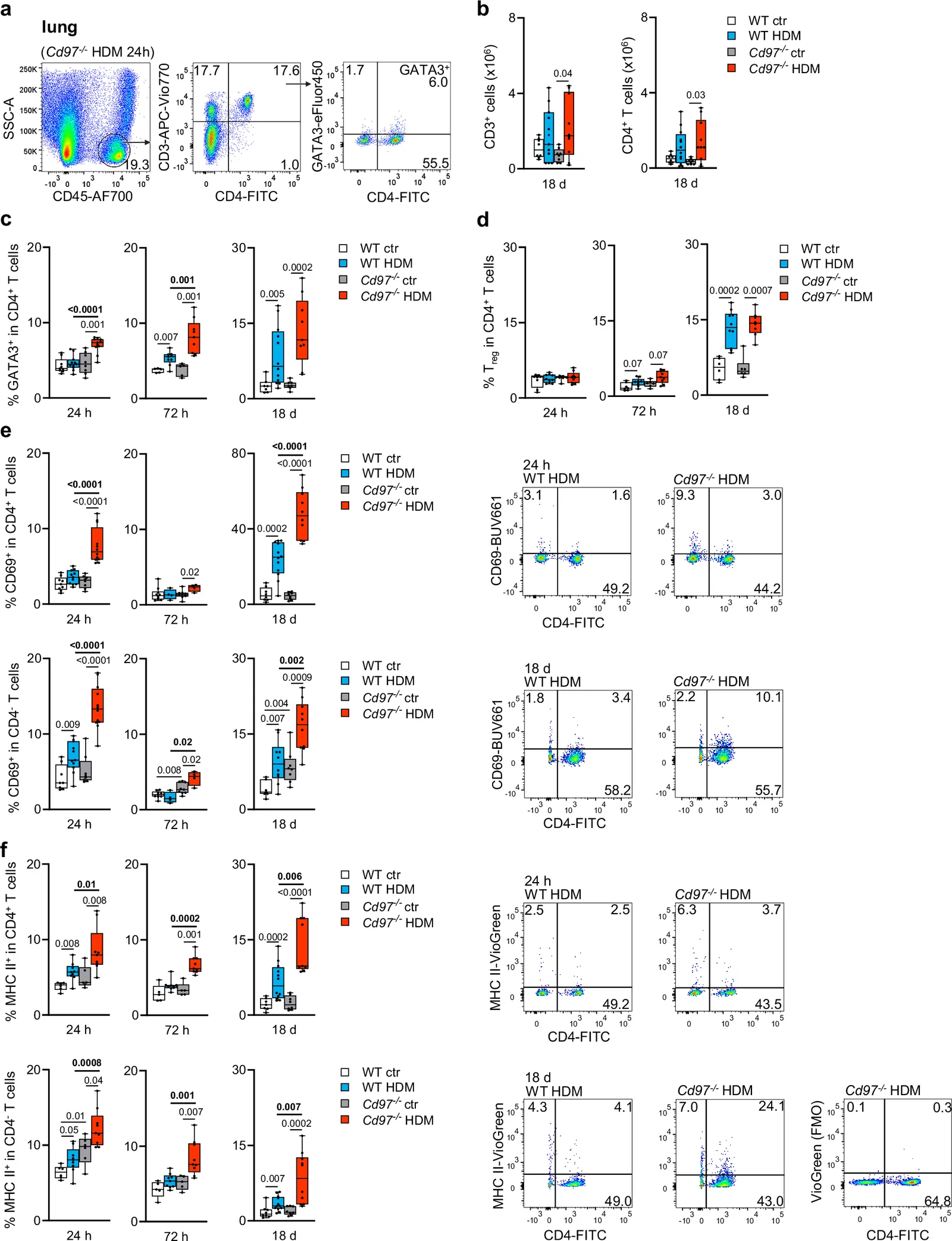
Increased numbers of GATA3^+^ and activated CD4^+^ T cells in the lungs of *Cd97*^*−/−*^ mice after sensitization. 24 and 72 h after HDM sensitization and at day 18 of the acute HDM asthma model, isolated lung cells were characterized by flow cytometry (protocol Fig. 4b). **a** Gating strategy to determine the frequency of GATA3^+^, T_regs_, CD69^+^ and MHC II^+^ CD4^+^, partly also CD4^-^ cells. **b** Absolute numbers of lung CD3^+^ and CD4^+^ T cells in naïve mice and at day 18. The frequency of **c** GATA3^+^ CD4^+^, **d** T_regs_ and of **e** CD69^+^, and **f** MHC II^+^ CD4^+^ and CD4^-^ cells within T cells were quantified. Right panels: Flow cytometry dot blots of a representative HDM-treated WT and *Cd97*^*−/−*^ mouse 24 h post-sensitization; for MHC II, the respective VioGreen FMO control is added. The frequency of positive cells (in 100 % of CD3^+^ cells) is indicated in the quadrants. **b** WT ctr, *Cd97*^*−/−*^ ctr: *n* = 8; WT HDM: *n* = 12; *Cd97*^*−/−*^ HDM: *n* = 10 (**c**–**f**). Sample sizes are reported in the following group order: WT ctr/*Cd97*^*−/−*^ ctr/WT HDM/*Cd97*^*−/−*^ HDM. 24 h: GATA3, CD69, T_reg_: *n* = 8/8/11/11; MHC II: *n* = 6/6/9/9. 72 h: GATA3, MHC II: *n* = 5/5/9/9, T_reg_: *n* = 6/6/9/9; CD69: *n* = 9/9/5/5. 18 d: GATA3: *n* = 7/7/12/10; T_reg_: *n* = 6/6/10/8; CD69: *n* = 7/7/10/10; MHC II: *n* = 8/8/12/10 mice/group. Data are presented as box-and-whisker plots, each data point represents an individual mouse, the centre line indicates the median, the box represents the IQR, and whiskers extend from minimum to the maximum. For statistical analysis, two-sided Mann–Whitney *U* test was used.

The key transcription factor GATA3 promotes type 2 immune responses by inducing type 2 cytokines and inhibiting type 1 specific factors^27^. Notably, absolute lung T cell and CD4^+^ T cell numbers were similar between WT and *Cd97*^*−/−*^ mice under baseline conditions and increased comparably following HDM sensitization (Fig. 7a, b). The frequency of GATA3^+^ among CD4^+^ T cells was higher in the lungs (Fig. 7c) and mLNs (Supplementary Fig. 8a, b) of *Cd97*^*−/−*^ compared to WT mice 24 and 72 h post-sensitization. At day 18, the frequency of these cells was similarly enhanced in both HDM-treated mice.

Furthermore, we examined the frequency of FoxP3⁺ CD25⁺ regulatory T cells (T_regs_), which maintain the balance between effector T cell subsets and promote tolerance to allergens. At 24 and 72 h their frequency among lung CD4⁺ T cells remained low (Fig. 7d). In mLNs, a higher proportion of T_regs_ among CD4⁺ T cells was observed in *Cd97⁻*^*/*^*⁻* mice post-sensitization (Supplementary Fig. 8c). By day 18, T_reg_ frequencies in lung and mLNs were similarly increased, up to ~ 3-fold, in both genotypes.

Moreover, early activation and intracellular cytokine expression by lung and mLN CD4^+^ T cells were quantified. 24 and 72 h after sensitization, the frequency of lung CD69^+^ and MHC II^+^ cells in both CD4^+^ and CD4^-^ T cells increased either exclusively or significantly more in *Cd97*^*−/−*^ compared to WT mice (Fig. 7e, f). On day 18, the frequencies of these cells were elevated in both mice, but were twice as high in *Cd97*^*−/−*^ mice compared to WT. In mLNs the frequency of CD69^+^ and MHC II^+^ CD4^+^ T cells was similarly enhanced in both HDM-treated mice (Supplementary Fig. 8d, e).

Finally, 24 h post-sensitization, restimulated isolated lung cells from *Cd97*^*−/−*^ mice showed increased frequencies of IL-4^+^ and IL-10^+^ CD4^+^ cells. By day 18, the frequency of IL-13^+^ and IL-10^+^ was enhanced in CD4^+^ cells of both mice, but stronger in *Cd97*^*−/−*^ compared to WT cells (Supplementary Fig. 9a–d). Intracellular cytokine expression by CD4^+^ mLN cells was relatively unchanged (Supplementary Fig. 9e–g).

### Lower *ADGRE5* levels in bronchial-derived MNPs of allergic asthmatics 24 h after HDM challenge

**Fig. 8:**
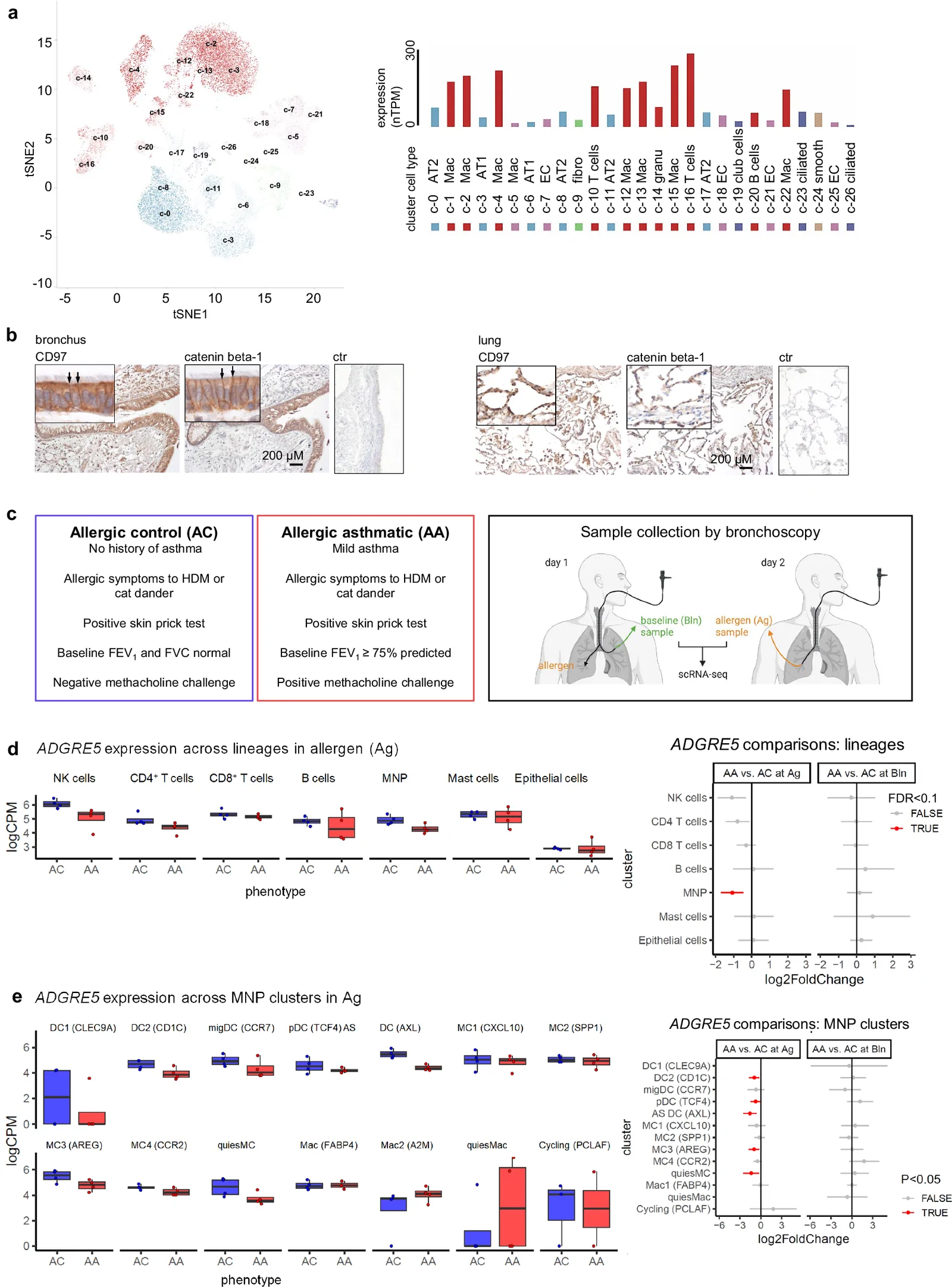
Expression of CD97/*ADGRE5* in the human lung. **a** *ADGRE5* expression in single cell type clusters identified in the lung and visualized by a UMAP plot (left; tSNE t-distributed stochastic neighbour embedding) and a bar chart (right); *ADGRE5* scRNA-seq data available from v24.proteinatlas.org^10,28^. The nTPM (transcripts per million) value represents the number of *ADGRE5* transcripts; cells: immune (Mac macrophages, granu granulocytes, T cells, B cells); epithelial (AT1 alveolar type 1 and AT2, ciliated, Club); fibro fibroblasts; smooth muscle cells; EC endothelial cells. **b** Immunostaining of lung cryosections: CD97 staining compared with catenin beta-1 and a control Ab; arrows: lateral cell-cell contacts. One representative micrograph series from three independent experiments is shown. **c**–**e** *ADGRE5* expression in a human model of asthma exacerbation^29^. **c** Left: Clinical characteristics of the allergic non-asthmatic controls (AC, *n* = 4) and allergic asthmatics (AA, *n* = 4) compared in this model. Right: Samples were obtained by endobronchial brushing of the lower airway mucosa at baseline (day 1, from the lingula) and 24 h after bronchoscopic segmental allergen challenge (day 2, from the right middle lobe). RNA-seq was performed on single cells. Created in BioRender. Aust, G. (2026) https://BioRender.com/be8pryq. *ADGRE5* expression analysis across **d** the main cell lineages and across **e** mononuclear phagocyte (MNP) clusters in the allergen-challenged samples (AC *n* = 4, AA *n* = 4). Left: logCPM (counts per million) of *ADGRE5* in the cell lineages and MNP clusters. Boxes represent the median (line) and interquartile range (IQR) with whiskers extending to the remainder of the distribution no more than 1.5x IQR with dots representing individual samples. Right: Forest plots demonstrate log2 fold-changes of *ADGRE5* expression, with error bars representing 95% confidence intervals, from a two-sided Wald test. Log2 fold-changes of the *ADGRE5* expression levels are significantly lower in lower airway-derived MNPs (pooled) after allergen challenge in AA compared to AC (FDR = 0.023). In detailed analyses of the MNP clusters **e**, log2 fold-changes of *ADGRE5* expression levels are significantly lower in cDC2s (that express *CD1C* etc.; *p* = 0.0134), plasmacytoid DCs (pDCs; *p* = 0.0410), Axl-Siglec6 (AS) DCs (*p* = 0.0012), macrophage-like monocyte-derived cells 3 (MC3; *p* = 0.0131), and quiescent MCs (quiesMC; *p* = 0.0172); Mac2 had zero expression of *ADGRE5* in some samples, so statistics could not be performed on that MNP cluster. None of the individual MNP cluster comparisons met the FDR < 0.1 threshold after correction for multiple comparisons, so significance in **e** is based on unadjusted *p* < 0.05.

Single-cell (sc)RNA-seq data from human tissues and blood show that circulating, tissue-infiltrating, and -resident immune cells are always those with the highest *ADGRE5* levels (www.proteinatlas.org)^10,28^. Among non-immune, tissue-specific cell types, especially lung epithelial cells, express moderate *ADGRE5* (Fig. 8a), confirmed at the protein level in immuno-stained human lung sections (Fig. 8b). CD97 localizes in lateral cell contacts and the cytoplasm of bronchial epithelial cells and in small airway epithelial cells.

To interrogate the specific relevance of CD97/*ADGRE5* in human allergic asthma, we evaluated the expression of *ADGRE5* in the lower airway mucosa of allergic asthmatics and allergic non-asthmatic controls using a model of allergen-induced asthma exacerbation^29^ (Fig. 8c). While *ADGRE5* expression was similar between both groups at baseline, its expression was significantly lower in MNPs after allergen challenge in allergic asthmatics compared to allergic non-asthmatics. No difference was seen in CD4^+^ T, CD8^+^ T, NK, B, mast, and airway epithelial cells (Fig. 8d). Among MNPs, cDC2s and immature monocyte-derived cells (MC4) were uniquely enriched in the lower airway mucosa of allergic asthmatics after allergen challenge compared to allergic controls^29^. These cDC2s, as well as plasmacytoid DCs (pDC), Axl-Siglec6 DCs (AS DCs), quiescent monocyte-derived cells (quiesMC), and macrophage-like monocyte-derived cells (MC3) had significantly lower *ADGRE5* expression in allergic asthmatics compared with non-asthmatics after allergen challenge (Fig. 8e).

These findings align with mouse models suggesting that reduced CD97/*ADGRE5* expression in specific MNP subsets promotes allergic asthma.

## Discussion

Here we demonstrate that the absence of CD97, induced either genetically or via CD97 Ab targeting, exacerbates allergic asthma in mouse models. CD97 attenuates the development of a Th2- and Tfh2-driven adaptive allergic response. Notably, a single CD97 Ab application before antigen sensitization exacerbated asthma. Given the transient availability of the CD97 Ab in circulation^19^, CD97 likely modulates processes during allergic sensitization. Interestingly, late-phase CD97 Ab treatment, when eosinophils infiltrate the lung, did not alter their BAL number and asthma severity, suggesting that these cells are not primarily involved. In contrast, CD97 Abs inhibited neutrophil accumulation at sites of inflammation, via CD55-independent, Fcγ receptor (FcγR)-dependent mechanisms^9,23,30^. Notably, FcγR types and number vary among granulocytes^31^, which, along with lung-specific lung CD97 Ab accumulation^19^, may explain the differing Ab effects across disease models.

CD97 is highly expressed in immune cells and also present in human and mouse bronchial and alveolar epithelial cells^9,10^. However, epithelial CD97 is not critical for asthma modulation, as disease severity was similar in *Nkx2.1*^*Cre/?*^ *Cd97*^*−/−*^ and WT mice, as were the levels of epithelial-released alarmins in *Cd97*^*−/−*^ and WT mice. CD97’s protective effect depends on immune cells, as shown in leukocyte-specific *Cd97*^*−/−*^ mice.

As CD97 is crucial for the early phase of the allergic immune response, we investigated its influence on DCs, which are key in T cell differentiation. Naïve *Cd97*^*−/−*^ and WT mice exhibited similar numbers and frequencies of lung and mLN cDC2s and moDCs, both of which initiate and sustain Th2-mediated immunity^3,4^. As recently shown, HDM sensitization increases the number and frequency of cDC2s and especially moDCs in the lungs and mLNs^3^. We confirmed these findings, observing increased frequencies of cDC2s and moDCs exclusively in the lungs of *Cd97*^*−/−*^ mice as early as 24 h after sensitization. moDCs present antigens to T cells directly in tissues rather than in lymphoid organs (reviewed in^32^). Concurrently, the frequency of migratory (activated) moDCs were increased in *Cd97*^*−/−*^ compared to WT mLNs.

To verify DC functions depending on CD97 we used BMDCs as a surrogate model. Activated DCs shape T cell differentiation towards Th1, Th2, or Th17 lineages through cytokine secretion^33,34^. *Cd97*^*−/−*^ BMDCs displayed enhanced activation and reduced IL-12 secretion upon stimulation. As IL-12 promotes Th1 differentiation, its reduction may favour Th2-skewed responses^35–37^. In contrast, levels of Th17-polarizing cytokines such as IL-1β, IL-6, and IL-23^38^ varied by the stimulus or were unchanged in *Cd97*^*−/−*^ compared to WT BMDCs. However, IL-17 levels did not differ between sensitised WT and *Cd97*^*−/−*^ mice in our asthma models. Furthermore, loss of CD97 in DCs enhances proliferation and promotes Th2 cytokine secretion of CD4⁺ OT-II cells. Ultimately, the CD97-altered BMDC function results in exacerbated allergic airway inflammation. RNA-seq analysis further revealed distinct transcriptional profiles in WT and *Cd97*^*−/−*^ DCs, indicating that their differences are already evident at the level of gene expression. Collectively, these findings suggest intrinsic functional differences between WT and *Cd97*^*−/−*^ DCs, the nature of which remains to be determined.

Additionally, CD97 loss modulates CD4^+^ T cell responses towards a stronger type 2 phenotype. Twenty-four hours post-sensitization, increased frequencies of GATA3^+^ CD4^+^ T cells were detected exclusively in the lungs and mLNs of *Cd97*^*−/−*^ mice. GATA3 is necessary and sufficient for type 2 cytokine production^39^. CD97 deficiency further increased the frequencies of activated CD69^+^ CD4^+^ and MHC II^+^ CD4^+^ lung T cells compared to WT mice. CD69 is transiently expressed on activated lymphocytes^40,41^ and can mark lung-resident memory cells mediating type 2 responses to inhaled allergens^42,43^. In humans, MHC II expression on T cells, a marker of activation (reviewed in ref. ^44^), is elevated on CD4^+^ T cells in severe asthma^45,46^, and identifies recently divided antigen-specific effector CD4^+^ T cells^47^, whereas in mice it is epigenetically silenced via CIITA repression^48^. Allergic asthma regulates epigenetically Th differentiation and memory^49^, and other immune functions^50^, likely enabling mouse lung and mLN T cells to express MHC II after HDM treatment.

Overall, conditional *Cd97*^*−/−*^ mice selectively targeting naïve CD4^+^ T cells would help clarify whether CD97 acts exclusively on DCs or also on CD4^+^ T cells.

Our data indicate that CD97 attenuates type 2 immune responses independent of ILC2s. Other immunoregulatory mechanism may be also involved. For example, the transient increase in the frequency of mLN T_regs_ in *Cd97*^*−/−*^ mice post-sensitization may represent an (ultimately ineffective) compensatory response to the heightened Th2 activity, as T_regs_ mitigate asthma severity^51^. Moreover, in the chronic asthma model prolonged exposure to allergens can induce tolerance^52,53^, and CD97 might play a role in regulating this process.

Further studies are required to identify the CD97 interaction partner(s) mediating its effects in allergic asthma. In humans, CD97 binds CD55^11^, THY1^12^, chondroitin-sulphate B (CS)^54^, and integrins αvβ3 and α5β1^55^; however, mouse CD97 lacks the CS binding domain^56^ and the integrin binding site^55^, precluding their analysis in our models. In OVA-induced asthma, *Thy1*^*−/−*^ mice showed reduced eosinophil and monocyte infiltration^57^, arguing against a role for THY1 in mediating CD97 effects. Moreover, THY1 is highly expressed on mouse T cells and DCs, but not in humans^58^, limiting translational relevance.

*Cd55* deficiency did not alter asthma development in our study. However, lung DCs retained CD55 expression from *Cd55.b*, and *Cd55*^*−/−*^ mice showed increased CD97 on lung immune cells. Thus, a contribution of CD97–CD55 interactions in allergic asthma cannot be excluded. Notably, naïve *Adgre5*^*−/−*^^16^ and our *Cd97*^*−/−*^ mice showed reduced splenic cDC2 numbers. cDC2s localize in the splenic bridging channels, where the CD97 NTF is shed upon interaction with CD55 on circulating erythrocytes^15^. This promotes splenic retention of cDC2s, whereas in *Adgre5*^*−/−*^ mice more cDC2s enter the circulation^16^. Such a spleen-specific mechanism is unlikely to operate in the lung or mLNs, which differ in microanatomy and lack direct DC–red blood cell contact. The release of the CD97 TIA after NTF shedding in the spleen activates Gα13–Rho GTPase signaling, altering the F-actin cytoskeleton and cellular migration^13,14,16^. CCR7 regulates DC homing to lymphoid tissues. However, F-actin and CCR7 levels in lung and mLN CD11b^+^ DCs, as well as BMDC migration toward CCR7 ligands, were similar in *Cd97*^*−/−*^ and WT mice.

Beyond this, reports implicating CD97-CD55 in a TCR co-regulatory complex, and in priming type 1 regulatory T cells (Tr1)^59–61^ have not been examined further. It is unknown whether CD55 is present at the T cell immunological synapse and interacts with CD97 there. CD97 localizes to the synapse, as shown in vitro. *Salmonella enterica* effector (SteD)-mediated CD97 depletion reduced mouse tumor (Mutu) DC-T cell interactions, likely via synapse destabilization^62^, and CD97 was identified in synaptic vesicles^63^. Knockdown of CD97 in naïve CD4^+^ T cells impaired activation by superantigen-presenting moDCs^64^. Notably, both DCs and T cells express CD97, however, its respective role in signal transduction and activation within the synapse remains unclear.

Importantly, our findings from mouse models, loss of CD97 enhanced allergic asthma, are supported by corresponding data obtained from our human study^29^. cDC2s were uniquely enriched in the lower airway mucosa and, together with other MNP clusters, showed reduced *ADGRE5* expression 24 h after bronchoscopic segmental allergen challenge in allergic asthmatics, compared to allergic non-asthmatics.

In summary, our mouse and human data identify CD97 as an immune co-regulator in allergic asthma, modulating disease severity via dampening adaptive type 2 responses. These findings support CD97 as a potential biomarker for future asthma diagnostics.

## Methods

### Institutional review board and informed consent statements

All mouse experiments were performed in accordance with the institutional and state guidelines, as approved by the Committee on Animal Welfare of Saxony, Germany (TVV 61/16, TVV 56/20, TVV 38/21, TVV 06/25). Research involving human participants was performed in accordance with the Declaration of Helsinki. Human lung samples for immunostaining cryosections were collected after written informed consent under a study protocol investigating the role of CD97/*ADGRE5* in mechanotransduction, approved by the Ethics Committee of Leipzig University (Reg. No. 269/17, 13.07.2017). Lower airway mucosal samples from allergic asthmatics and allergic non-asthmatic controls were obtained with written informed consent from study participants. The protocol of this nonrandomized, nonblinded mechanistic study was approved by the Massachusetts General Brigham Institutional Review Board (IRB# 2007P001050) and registered at clinicaltrials.gov (NCT00595491).

### Mouse study

#### Mice

C57BL/6 *Cd97*^*−/−*^ and the corresponding WT mice were characterized for tissue-specific CD97 expression^9^. To attribute the CD97-dependent effects in asthma to a specific cell type, we generated a *Cd97*^*loxP/loxP*^ mouse in which exons 7–9 of *Cd97* are flanked by loxP sites (Supplementary Fig. 2a–d). By crossing this line with cell type-specific Cre-recombinase mice, we produced conditional *Cd97*^*−/−*^ mice. C57BL/6J-Tg(Nkx2-1-cre)2Sand/J^65^ and C57BL/6-Tg(Csf1r-cre)1Mnz/J mice^66^ were obtained from The Jackson Laboratory (Bar Harbor, US). Furthermore, we used *Cd55*^*−/−*^ mice^22^, formerly designated as *Daf1*^*−/−*^, and their corresponding WT mice. Female BALB/cByJ mice (6-8 weeks old) were purchased from Elevage Janvier Laboratory (Le Genest St Isle, France). C57BL/6-Tg(TcraTcrb)425Cbn/Crl (OT-II) mice, expressing an αβTCR specific for OVA 323-339, were obtained from Charles River France at 6 weeks of age. All mice underwent a 7-day adaption period before the start of experiments. The animals were maintained in groups of 3–6 mice per cage in the animal facility at the University of Leipzig (Germany) under conventional conditions with a room temperature of 21.5–23 °C, an average of 55% humidity, and a 12 h day/night rhythm. Only female mice were studied, as they are more susceptible in mouse models of asthma^67^. The mice were euthanised by CO₂ inhalation. Cardiac arrest was confirmed by palpation for a heartbeat and verification of the absence of respiration.

#### Asthma induction and Ab treatments

Female *Cd97*^*−/−*^ and WT mice (8–10 weeks old) were sensitized with OVA (20 μg, Merck KGaA, Darmstadt, Germany) adsorbed to 2 mg of an aqueous solution of aluminium hydroxide and magnesium hydroxide (Imject Alum, Perbio Science, Bonn, Germany) intraperitoneally (i.p.) on days 1 and 14, followed by 20 μg OVA in 40 μl normal saline given intranasally (i.n.) on days 14–16 and 21–23. Control mice received i.p. injections of alum alone and normal saline. AHR was measured on day 24, and mice were sacrificed on day 25. To induce a chronic asthma-like phenotype, *Cd97*^*−/−*^ and WT mice were sensitized i.p. with OVA on days 1 and 14 and challenged with OVA i.n. twice per week for 8 weeks. AHR was measured on day 71, and mice were sacrificed the following day. For the HDM-induced asthma model female *Cd97*^*−/−*^, conditional *Cd97*^*−/−*^, WT C57BL/6, or BALB/c mice (8–10 weeks old) were sensitized via the airways with 5 µg HDM extract (*Dermatophagoides pteronyssinus*, 646 EU/ml endotoxin, LOT 360923, Greer Laboratories Inc., Lenoir, USA) in 40 µl saline on day 1. This was followed by i.n. administration of 5 µg HDM on days 7–11 and 14–16. The anti-mouse CD97 Ab 1B2^19^ or the IgG1 isotype control, an anti-human EMR3 Ab (clone 3D7, since mice do not have *Adgre3*), were applied i.p. (0.5 mg in 200 µl) at different time points (protocol 1: day 0, 7, 14; protocol 2: day 7, 14; protocol 3: day 0) during HDM-induced asthma in BALB/c mice.

Mice were randomly allocated to the treatment groups. Tissue and cell processing was performed blinded to genotype and treatment, with mice identified by numeric codes only.

#### Measurement of AHR

Lung resistance (R_L_) was measured by invasive plethysmography (EMKA Technologies, Paris, France) in response to inhaled methacholine (Merck). Therefore, mice were anesthetized (100 mg/kg ketamine and 10 mg/kg xylazine, Bayer, Leverkusen, Germany), intubated, and mechanically ventilated at a tidal volume of 0.2 ml and a frequency of 150 breath/min. Baseline R_L_ and responses to aerosolized 0.9% NaCl were measured first, followed by responses to increasing doses (2.5–40 mg/ml) of aerosolized methacholine.

#### Collection of bronchoalveolar lavage (BAL) fluid

The extent of inflammation in asthma development was characterized by the differential leukocyte count within the BAL fluid. BAL fluid was obtained post-euthanasia through a small incision in the trachea, followed by the insertion of a syringe to lavage the right lung three times with 400 µl NaCl 0.9%. The total cell count within the fluid was determined using a hemocytometer. The cytospins were then subjected to Diffquick (Medion Diagnostics AG, Düdingen, CH) staining, followed by cell differentiation into eosinophils, macrophages, lymphocytes and neutrophils according to conventional morphological criteria^68^.

#### Lung histology

The left mouse lung was fixed in 10% formalin and stained with Haematoxylin & Eosin (HE, Merck) or with a Periodic Acid Schiff (PAS) staining protocol (Schiff's reagent, Merck). For quantification and objective evaluation of the degree of histological inflammation or mucus production, whole lung sections were scanned with a digital camera (5 shots per lung) and analysed with the HistoClick-Software based on morphometric image analysis^20,21^. The degree of inflammation or mucus production expressed by the number of pixels, which correlate to the stained lung sections. Airway remodelling was assessed by measuring total matrix deposition on Martius Scarlet Blue (MSB)-stained lung sections^69^. Furthermore, lung sections were subjected to immunostaining with α-smooth muscle actin (SMA) primary Ab (Abcam, Cambridge, UK) to distinguish smooth muscle cells, followed by a horse radish peroxidase (HRP)-labeled secondary Ab (Abcam) and colorimetric detection using 3,3’ diaminobenzidine (DAB)^20,21^.

To characterize conditional *Cd97*^*−/−*^ mice for CD97 expression in the lung, cryosections were co-stained with the CD97-PE (Miltenyi Biotec, Bergisch Gladbach, Germany; cat. no. 130-110-329, dilution 1:10) and EpCAM (BD Biosciences Heidelberg, Germany, cat. no. 552370, dilution 1:400) Abs. Subsequently, the EpCAM Ab was detected with donkey anti-rat Alexa 488-labeled IgG (Thermo Fisher, Darmstadt, Germany; cat. no. A21208, dilution 1:400).

To quantify lung CD11c^hi^ MHC II^hi^ DCs, consisting of cDC2s, moDCs, and cDC1s^24^, two non-serial cryosections of the left lung lobe were co-stained with CD11c-PE (Miltenyi, cat. no. 130-110-838; 1:50) and MHC II VioBright FITC (Miltenyi, cat. no. 130-124-709; 1:25). The sections, each with an area of > 20 mm^2^, were scanned using a BZ-X800 (Keyence GmbH, Frankfurt aM, Germany). The number of CD11c^hi^ MHC II^hi^ DCs and DAPI^+^ nuclei was quantified using ImageJ/Fiji (fiji.sc).

#### IgE assay

OVA- or HDM-specific IgE serum levels were measured by sandwich ELISA. Briefly, 96-well plates (Nunc, Roskilde, Denmark) were coated overnight with 10 µg/ml OVA or 25 µg/ml HDM. After washing and blocking, serum samples were added and incubated overnight at 4 °C. Plates were then washed and incubated with 100 µl of biotin-anti-mouse IgE (BioLegend, CA, USA) for 1 h, followed by washing and avidin-HRP (BioLegend) for 30 min. 100 µl tetramethylbenzidine substrate solution was added and incubated in the dark for 30 min. The optical density (OD) was determined at 450 nm using a BioTek microplate reader (BioTek Instruments, Bad Friedrichshall, Germany). The specific IgE titer was calculated by logarithmical regression as the reciprocal dilution of the sera, where the extinction was 2-fold the background extinction.

#### In vitro cytokine production

5 × 10^6^ isolated splenocytes or mLN cells were restimulated in vitro with 200 μg/ml OVA or 50 µg/ml HDM in RPMI1640 medium/10% FCS. Cytokines were measured in duplicate in the cell supernatants after three days using DuoSet ELISA kits (R&D Systems, Minneapolis, US) according to the manufacturer’s instructions^20,21^.

#### Collagen analysis

The collagen content of lung tissue homogenates was measured by a biochemical assay according to the manufacturer’s instructions (Sircol collagen assay, Biocolor, Ireland) as previously described^20,21^.

#### Quantitation of lung alarmins

To quantify TSLP and IL-33, lung tissue was collected in 500 µl PBS with a complete protease inhibitor cocktail (Merck), homogenized (Kimble Pellet Pestle, Merck), and centrifuged (10 min, 4 °C, 16,000 × *g*). The supernatants were collected for further analysis. Protein concentrations were quantified using the Bradford assay (Bio-Rad Laboratories, Feldkirchen, Germany). TSLP and IL-33 in the supernatants and BAL fluid were measured using DuoSet ELISA kits according to the manufacturer’s instructions.

#### Lung, spleen, and mLN preparation

Lung tissue was placed on ice, and the enzyme solution (Lung Dissociation Kit mouse, Miltenyi) was gently injected into each lobe. The tissue was minced and subsequently dissociated using the gentleMACS Octo Dissociator with Heaters (Miltenyi; program 37C_m_LDK_1). The resulting cell suspension was passed through a 70 µm strainer and washed with PBS (0.5% BSA, 1 mM EDTA; pH 7.2) at 300 g. Erythrocytes were lysed using the ammonium chloride-potassium lysis buffer (150 mM NH_4_Cl, 10 mM KHCO_3_, 0.1 mM EDTA; pH 7.3) for 2 min and washed twice. After passing the cells through a 40 µm strainer, they were stained for flow cytometric analysis.

The CD45^+^ lung leukocytes were isolated by MACS magnetic cell separation (CD45 MicroBeads, mouse; Miltenyi), resulting in > 95% enrichment, as confirmed by flow cytometry. Spleens were mashed through a 70 μm cell strainer in RPMI 1640/10% FCS using the plunger end of a syringe. The resulting cells were washed once with PBS. Erythrocytes were lysed in 2 mL ammonium chloride-potassium buffer for 2 min, followed by two PBS washes. mLNs were manually collected under a stereo microscope, homogenized in RPMI 1640/10% FCS using a 0.1 mL micro tissue grinder (pestle clearance 0.1–0.15 mm), and washed once before use.

#### Culture, treatment, and transfer of BMDCs

BM cells were collected from one naïve *Cd97*^*−/−*^ and one WT in parallel, depleted of red blood cells using ammonium chloride lysis buffer, and grown in RPMI1640 medium/10% FCS and 20 ng/ml rmGM-CSF for 7 days. Mouse CD11c^+^ cells were separated by using magnetic cell sorting systems and CD11c Microbeads (Milteny Biotec, Bergisch Gladbach, Germany). To induce BMDC maturation, cultures were treated with OVA (200 µg/ml), LPS (10 ng/ml, Merck), recombinant mouse (rm) TNF (100 ng/ml), and rm sCD40L (1 µg/ml, both Immunotools, Friesoythe, Germany) for 18 h at day 7. All cytokines were measured using DuoSet ELISA kits (R&D Systems, Minneapolis, US) according to the manufacturer’s instructions.

Sensitization by intrapulmonary transfer of OVA-pulsed BMDCs was conducted as previously described^70,71^. Here, BMDCs (2 × 10^6^ cells in 80 μl PBS) from *Cd97*^*−/−*^ or WT mice were pulsed with 200 μg/ml OVA for 24 h and then adoptively transferred into the trachea of anesthetized naïve WT mice. From day 10 onwards, mice were challenged with OVA i.n. for 3 consecutive days and sacrificed on day 14^20^.

#### DC co-culture with CD4^+^ OT-II cells

CD4^+^ T cell from the spleen of OT-II mice were isolated by magnetic cell sorting (CD4^+^ T cell Isolation Kit, Miltenyi) and stained with 5 µM CFSE (Hycultec GmbH, Beutelsbach, Germany). 1.5 × 10^4^ OVA-pulsed (200 µg, 24 h) CD11c^+^ BMDCs or CD11c^+^ lung cells of *Cd97*^*−/−*^ and WT mice were co-cultured with 1 × 10^5^ or 2 × 10^5^ CD4^+^ cells, respectively, within 96-well plates. After 4 days T cell proliferation was quantified by flow cytometry. Viable CD4^+^ cells were analysed for CFSE expression, with individual CFSE^+^ peaks gated and the percentage of cells that had accomplished x rounds of proliferation plotted on the graph. Cytokine production was measured in the cell culture supernatant using DuoSet ELISA kits.

#### BMDC migration assay

BMDCs were stimulated with 10 ng/ml LPS for 18 h. 1.5 × 10^5^ BMDCs in 100 µl RPMI1640/5% FCS were added to 24-well transwell inserts (polycarbonate membrane, 5 µm pore size; Merck). The lower chamber contained 0.6 mL RPMI1640/5%FCS and either 100 ng/ml CCL19 or CCL21 (BioLegend) in the bottom well. Cells were incubated for 3 h at 5% CO_2_, 37 °C. Migrated cells recovered from the lower chamber were quantified with a 3 L Cytek Aurora full-spectrum flow cytometer (Cytek Biosciences, CA, USA).

#### Flow cytometric analysis

The optimal concentration for each antibody (Ab; Supplementary Table 1) was determined by titration. To optimize panel performance, each Ab was sequentially replaced with fluorescence minus one (FMO) controls. Fc receptor (FcR) binding was assessed using isotype-matched control Abs conjugated to the corresponding fluorophores. When possible, REAfinity Abs (Miltenyi), which lack FcR binding, were used. If necessary, cells were preincubated with mouse FcR Blocking Reagent (Miltenyi).

Typically, 2–5 × 10⁵ isolated cells were stained with the respective Abs for 30 min at 4 °C, followed by three washes with PBS/0.5% BSA, 1 mM EDTA (washing buffer). Cells were fixed in PBS/1% formaldehyde. For transcription factor detection, fixed cells were permeabilized and stained using the Fixation/Permeabilization Buffers (FoxP3 Staining Kit, Miltenyi). For intracellular cytokine staining, lung and mLNs cells were pre-stimulated for 4 h at 37 °C in RPMI 1640/10% FCS with 50 ng/ml PMA, 750 ng/ml ionomycin, and 10 µg/ml brefeldin A (all Thermo Fisher). Cells were then harvested, washed, surface-stained, and fixed in 2% formaldehyde/PBS for 30 min at room temperature, followed by a washing step. Subsequently, cells were washed with PBS/0.3% saponin and incubated with cytokine-specific Abs in PBS/0.3% saponin for 30 min at 4 °C, washed, and fixed in 1% formaldehyde/PBS.

Cells were measured on a BD LSRFortessa X-20 Cell Analyser (BD Biosciences), and cytometry data were analysed using FlowJo Software version 10 (FlowJo LLC, Ashland, USA). The FlowClean plugin was applied, automatically detecting and removing abnormal events based on the time channel. Sample quality was assessed using the Sample Quality Check function in FlowJo. Doublet discrimination was performed using FSC-H vs. FSC-A gating. FSC and SSC thresholds were set to remove electronic noise, and a minimum FSC threshold was applied to exclude debris. Dead cells were excluded by Fixable Viability dye staining (BD Biosciences). The dataset was refined to single, viable cells; these preprocessing steps are not shown in the figures depicting gating strategies.

CD11b^+^ DCs (cDC2s and moDCs) are characterized as CD45^+^ CD11c^hi^ MHC II^hi^ Siglec F^−^ CD11b^+^ (or SIRPa^+^) XCR1^−^. cDC2s are CD64^−^, while moDCs are CD64^+^^24^. CD11b (or SIRPa) and XCR1 are mutually exclusive on cDC2s and cDC1s^24^. Figure 4c shows a representative example of the gating strategy.

#### RNA isolation and qRT-PCR

Frozen lung tissue was homogenized at 2 × 20 s, 5000 rpm in a Precellys sample homogenizer using the CKMIX tissue homogenizing kit (Bertin GmbH, Frankfurt am Main, Germany) with 600 µl RLT buffer (Qiagen, Hilden, Germany). 5 × 10^5^ CD45^+^ lung leukocytes were restimulated in vitro with 50 µg/ml HDM for 6 h. Cells were harvested and homogenized in 350 µl RLT buffer using Qiashredder spin columns (Qiagen). RNA was extracted using the RNeasy Kit (Qiagen) and transcribed with the SuperScript IV Reverse Transcriptase (Thermo Fisher, Darmstadt, Germany). Relative gene levels were quantified using a SYBR Green Master Mix (Thermo Fisher) and gene-specific primers on a 7500 Real-Time PCR System amplificator (Applied Biosystems, California, USA) with the ΔΔCt method. Transcript levels were normalized to *Rps29*. Primer sequences are provided in Supplementary Table 2.

### Human study

#### Immunostaining lung sections

Human lung serial paraffin sections were immune-stained after antigen retrieval (citrate buffer, pH 6.0, 20 min at 120 °C in a pressure cooker) with Abs directed to CD97 (Merck, Cat. No HPA013707) and catenin beta-1 (Santa Cruz Biotechnology Inc., Dallas, USA; Cat No sc-7199) overnight at 4 °C. The CELSR3 Ab (Merck, Cat. No HPA062866) was used as a control Ab, as lung epithelial cells do not express *ADGRC3*, which encodes CELSR3 (http://www.proteinatlas.org). Afterwards, the sections were incubated with the respective HRP-labeled secondary Ab 1 h min at room temperature, followed by colorimetric detection using DAB. Finally, the sections were counterstained with hematoxylin.

#### Lower airway mucosal sample collection and scRNA-seq

Lower airway mucosal samples were collected before and 24 h after segmental allergen challenge using standardized HDM (*Dermatophagoides pteronyssinus;* Greer Laboratories) or cat hair extract (*Felis catis*; Greer Laboratories), as previously described^29^. In brief, male and female participants between 18 and 50 years old with a clinical history of allergic rhinitis and/or conjunctivitis to HDM and/or cat hair and a positive skin prick test to the same allergen were enrolled. Allergic asthmatics additionally had a clinical history of mild asthma, a baseline forced expiratory volume (FEV1) in one second greater than or equal to 75% of the predicted value, and a positive methacholine challenge test. Participants underwent research bronchoscopy with baseline endobronchial brushings collected from the lingula using a 4 mm sterile nylon cytology brush under direct visualization. Allergen (2 mL) was then administered to the lateral segment of the right middle lobe, and 24 h later allergen-challenged endobronchial brush samples were obtained. Endobronchial brush samples were collected in phenol red-free RPMI 1640 (Thermo Fisher) with 2% human AB serum and ROCK inhibitor Y-27632 (Bio-Techne Co, Minneapolis, US) on ice, and all samples were processed within 60 min of collection. Single-cell suspensions were depleted of dead cells using annexin V-conjugated beads (STEMCELL Technologies, Cambridge, US) and of red blood cells using Abs directed against glycophorin A (STEMCELL). Cell viability was assessed via 0.4% trypan blue staining and manual cell counting via hemocytometer before and after dead cell and red blood cell removal to achieve >95% viability. Viable cells were resuspended at a concentration of 800–1000 cells per μL and loaded onto the 10X Chromium controller instrument (10X Genomics, Pleasanton, USA), and gene expression libraries were sequenced on an Illumina NextSeq 500/550 system, as previously described^29^.

#### Statistics

Data were analysed using GraphPad Prism10.2 (GraphPad Software, Boston, MA, USA). Unless otherwise stated, comparisons between two independent groups were performed using the two-tailed Mann–Whitney *U* test. In all box-and-whisker plots, each data point represents an individual mouse. The centre line indicates the median, the box represents the interquartile range (IQR), and the whiskers extend from the minimum to the maximum value unless otherwise specified. Lung resistance data are presented as the median with IQR.

All mouse experiments were performed independently at least three times unless otherwise stated. The number of mice per group (*n*) is indicated in the corresponding figure legends. Differences between experimental groups (control, disease, genotype, and antibody-treated groups) were considered statistically significant at *p* < 0.05, exactly *p*-values are indicated.

Gene expression comparisons between disease groups and experimental conditions for the human data were performed on pseudobulk count matrices using the DESeq2 package (v1.32.0, R v4.1.0), and significance was determined using a Wald test (two-sided, false discovery rate, FDR < 0.1) for lineage-level comparisons and *p* < 0.05 for MNP clusters.

### Reporting summary

Further information on research design is available in the Nature Portfolio Reporting Summary linked to this article.

## Supplementary information


Supplementary Information
Description of Additional Supplementary Files
Supplementary Data 1
Reporting Summary
Transparent Peer Review file


## Source data


Source Data


## Data Availability

Mouse RNA-seq raw data, metadata, and processed data have been deposited in the GEO database (https://www.ncbi.nlm.nih.gov/geo/query/acc.cgi?acc=GSE339436). Human scRNA-seq count matrices and related data are deposited in the GEO database (under accession number GSE193816 https://www.ncbi.nlm.nih.gov/geo/query/acc.cgi?acc=GSE193816), and raw human sequencing data are available in the controlled access repository dbGaP (www.ncbi.nlm.nih.gov/gap/) under accession number phs003101.v1.p1 (https://dbgap.ncbi.nlm.nih.gov/beta/study/phs003101.v1.p1/#study). Source code for data analysis in each main Figure is available on GitHub (GitHub - villani-lab/airway_allergic_asthma · GitHub) and has been archived to Zenodo (https://zenodo.org/badge/latestdoi/412260708). A user-friendly portal is available to browse the single-cell data generated in this manuscript. Users can query the single-cell clustering solutions, visualize gene expression, and browse differential expression of genes. Users may navigate the portal by visiting https://villani.mgh.harvard.edu/allergy-asthma. All other relevant raw data from figures generated in this study have been provided in the Source Data file. All other data are available in the article and its Supplementary files or from the corresponding author upon request. Source data are provided with this paper.
