# Supplementary material for: CD97/ADGRE5 attenuates the induction of adaptive type 2 immune responses in allergic asthma
Source: Nat Commun. 2026 Aug 28;17:9226. doi: 10.1038/s41467-026-76948-9 (PMC13522487; doi:10.1038/s41467-026-76948-9)
Supplement: Supplementary file 1 [file 41467_2026_76948_MOESM1_ESM.pdf]

CD97/*ADGRE5* attenuates the induction of adaptive type 2 immune responses in allergic asthma

Gabriela Aust, Anna-Lena Hoh, Jehan Alladina, Neal P. Smith, Florian Höhna, Bingyu Wang, Christiane Kerner, Marita Wagner, Knut Krohn, Arkadiusz Pierzchalski, Nele Häussler, Alexandra Chloe Villani, Josalyn L. Cho, Benjamin D. Medoff, Ana C. Zenclussen, Matthias Steinert, Jörg Hamann, Marianne Quaas, and Tobias Polte

SUPPLEMENTARY INFORMATION

Supplementary Figures

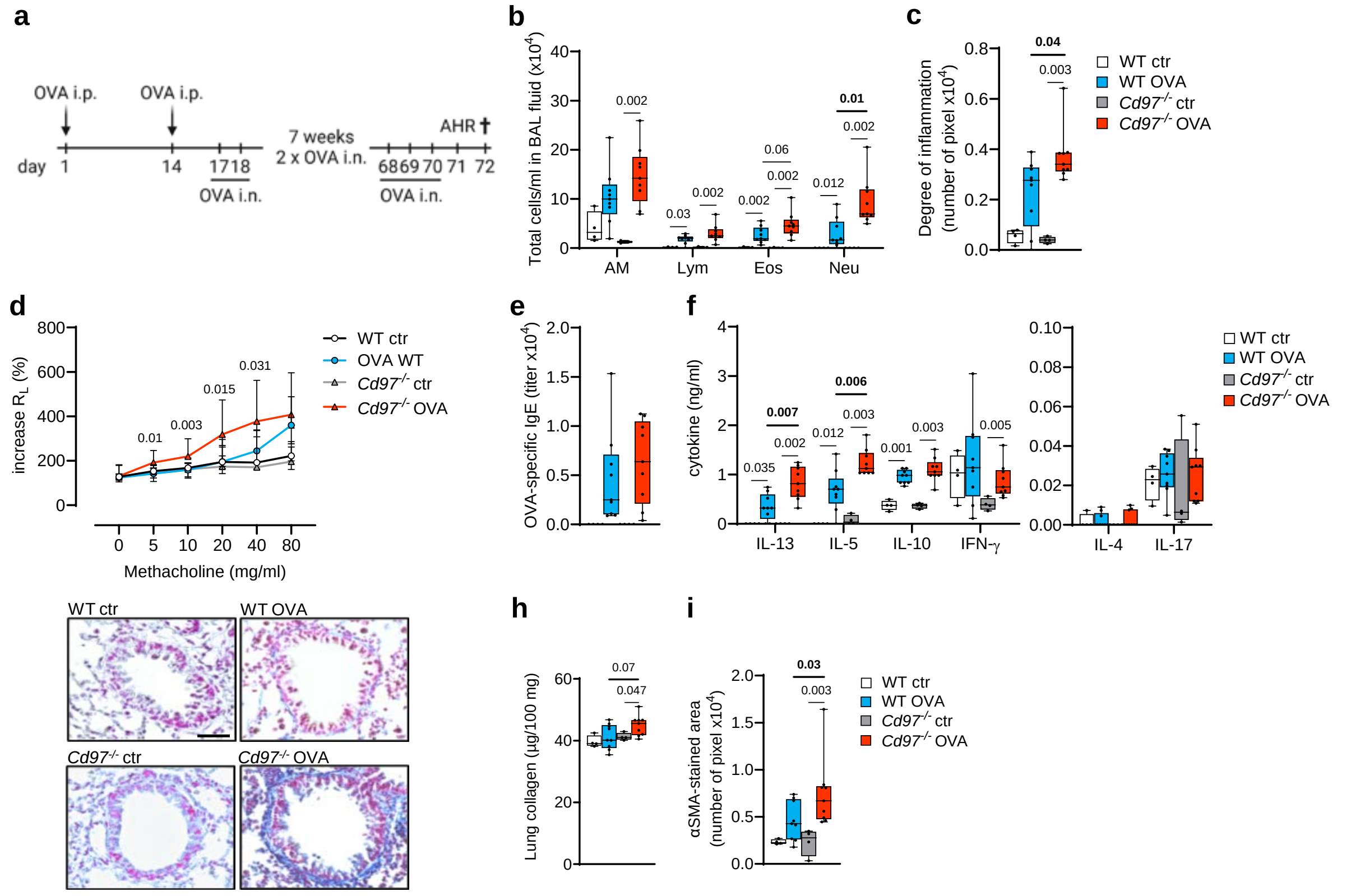

Supplementary Fig. 1. Loss of CD97 leads to an exacerbated chronic asthma phenotype in OVA-sensitized mice.

(a) Using a chronic asthma protocol, (b) total cell number in BAL fluid, (c) inflammation of lung tissue, (d) lung resistance (AHR), (e) OVA-specific IgE levels and (f) cytokine production in the spleen were determined in WT and *Cd97*$^{-/-}$ mice. (g) Lung sections of OVA-sensitized WT and *Cd97*$^{-/-}$ mice were immuno-stained for matrix deposition of collagen and fibrin with Martius scarlet blue. (h) The amount of total lung collagen and (i) numbers of airway smooth muscle cells (αSMA-stained lung sections, quantified by an investigator-independent computer-based analysis) were determined. Data of two independent experiments. (b, c, e-i) WT ctr, *Cd97*$^{-/-}$ ctr: n = 4, WT OVA, *Cd97*$^{-/-}$ OVA: n = 9 mice/group. (g) Representative micrographs from 4 (WT ctr, *Cd97*$^{-/-}$ ctr) and 9 (WT OVA, *Cd97*$^{-/-}$ OVA) mice are shown. In figures where data are presented as box-and-whisker plots, each data point represents an individual mouse, the centre line indicates the median, the box represents the IQR, and whiskers extend from minimum to the maximum. Lung resistance data (d) are presented as median with IQR. For statistical analysis, 2-sided Mann-Whitney U test was used.

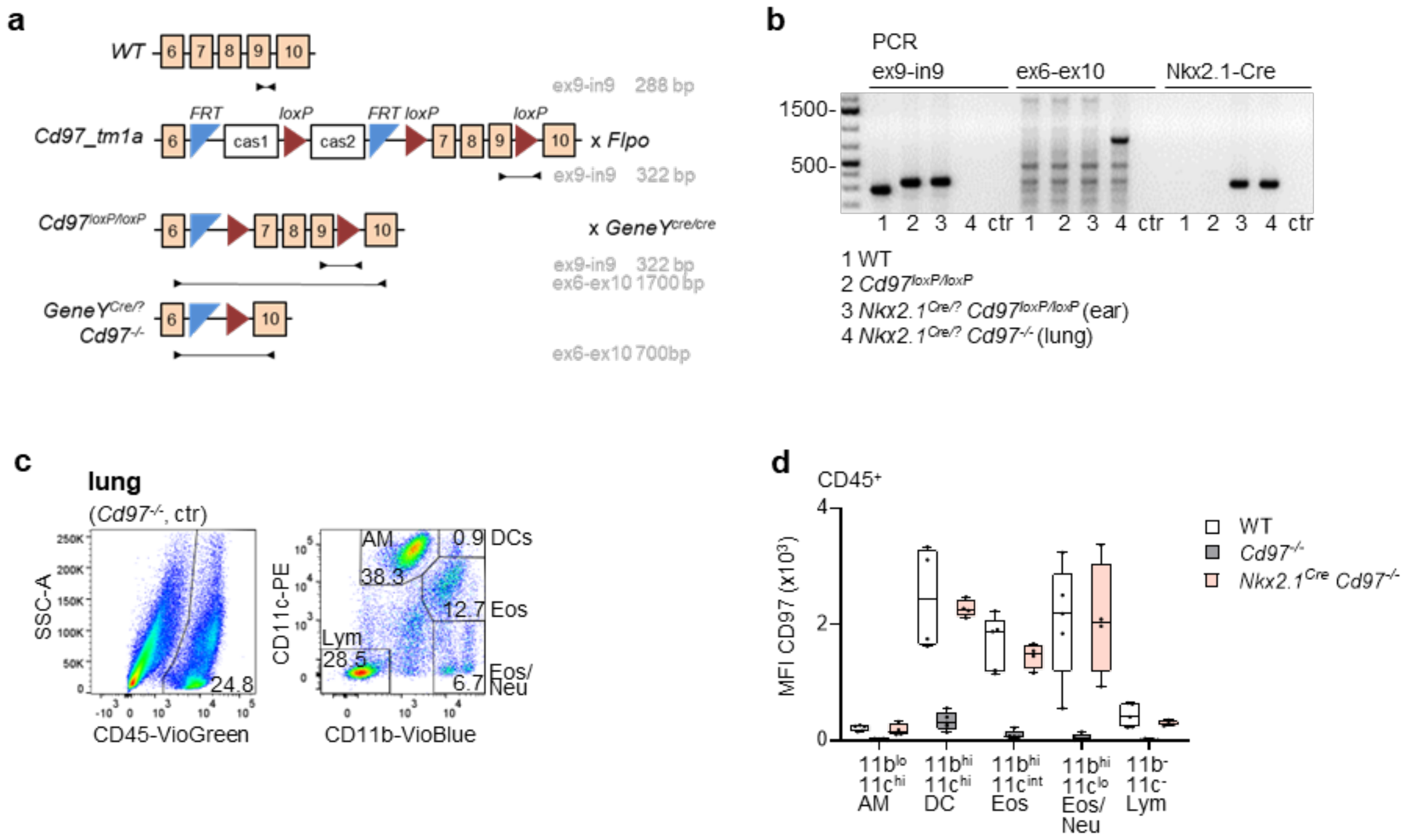

Supplementary Fig. 2. Generation and characterization of conditional *Cd97*$^{-/-}$ mice.

(a) Mutant *Cd97/Adgre5* embryonic stem cells, obtained from the European Mouse Mutant Cell Repository (EuMMCR; Munich, Germany) were transferred into *C57BL/6* mice. The critical exons 7-9 of *Cd97* (chromosome 8) are flanked by loxP sites. To remove two gene cassettes in the generated *Cd97_tm1a* mouse, a "conditional ready" (floxed) allele was created by *Flp* recombinase expression in mice carrying this allele. Subsequent tissue-specific Cre expression resulted in a tissue-specific conditional *Cd97Ko.* (b) PCRs examining exemplarily the resulting *Nkx2.1*$^{Cre/?}$ *Cd97*$^{-/-}$ mice. PCR strategy, primer locations and PCR products see a. (c-d) Characterization of isolated lung cells of WT, *Cd97*$^{-/-}$, and *Nkx2.1*$^{Cre/?}$ *Cd97*$^{-/-}$ mice by flow cytometry. (c) Flow cytometry, gating strategy to quantify the expression of CD97 in lung leukocyte subsets. After excluding doublets and debris, leukocytes and non-immune cells (CD45^{-}) were separated by CD45. Leukocytes were subtyped by CD11b and CD11c. CD11b^{low} (11b^{lo}) CD11c^{high} (11c^{hi}) cells are mainly alveolar macrophages (AM), 11b^{hi} 11c^{hi} DCs, 11b^{hi} 11c^{int} eosinophils (Eos), 11b^{hi} 11c^{lo} eosinophils/neutrophils (Eos/Neu), and 11b^{-} 11c^{-} lymphocytes (Lym). The percentage of each subset is indicated within the gate (relative to 100 % of all cells in the respective window); a representative untreated *Cd97*$^{-/-}$ mouse is shown. (d) Quantitation of CD97 (median fluorescence intensity, MFI) on these lung leukocyte subsets in the indicated, untreated mouse strains; (d) WT, *Cd97*$^{-/-}$: n = 5; *Nkx2.1*$^{Cre/?}$ *Cd97*$^{-/-}$ n = 4 mice/group. Data are presented as box-and-whisker plots, each data point represents an individual mouse, the centre line indicates the median, the box represents the IQR, and whiskers extend from minimum to the maximum.

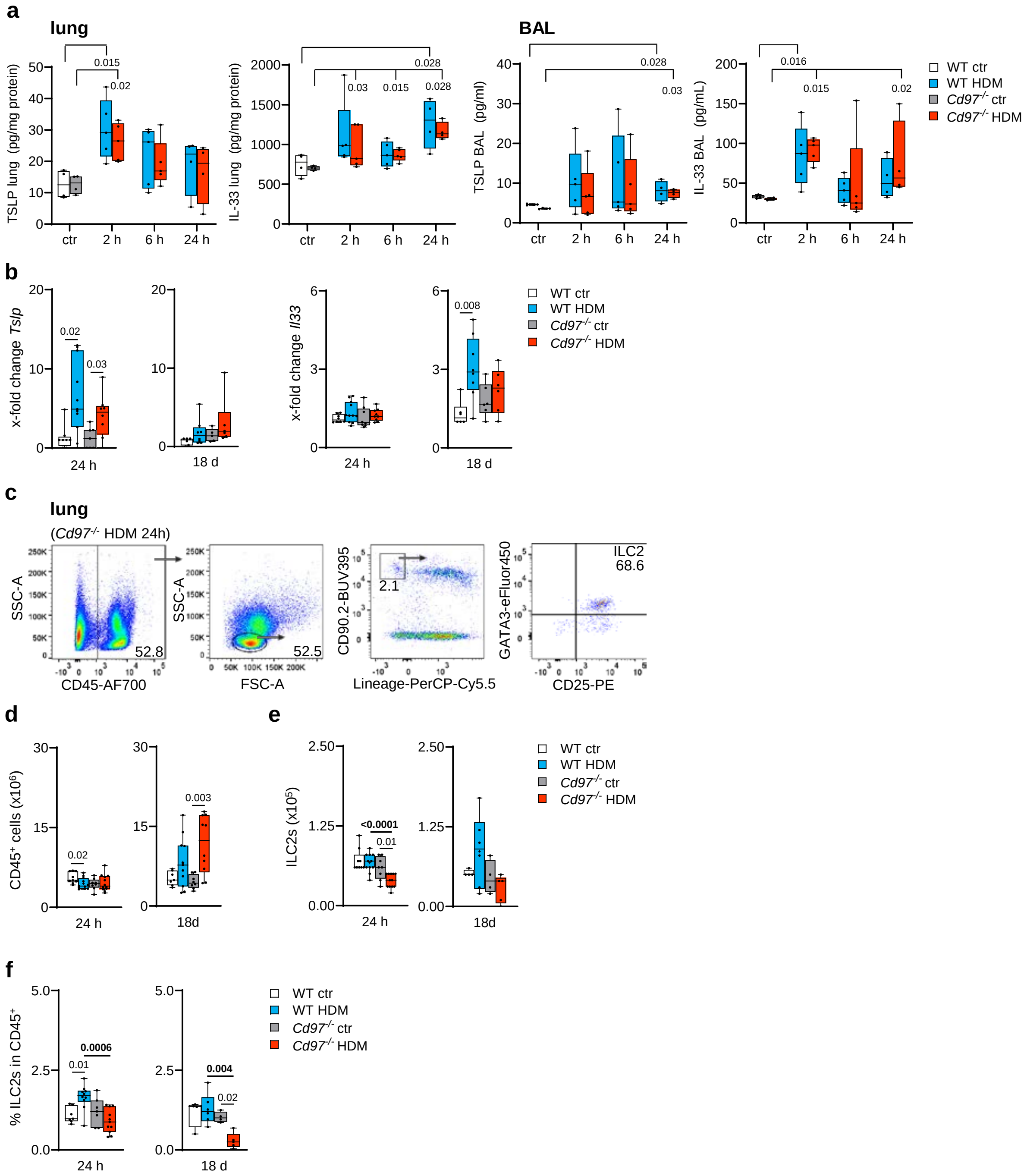

Supplementary Fig. 3. Alarmins and ILC2s in the lung of WT and *Cd97*$^{-/-}$ mice

(a) Levels of TSLP and IL-33 in lung homogenates and BAL fluid of untreated mice and 2, 6 and 24 h after HDM sensitization. (b) *Tslp* and *Il33* mRNA levels in lung tissue samples of WT and *Cd97*$^{-/-}$ mice 24 h post-sensitization and at day 18 of the acute asthma model. (c-f) Isolated lung cells were characterized for the presence of ILC2s. (c) Gating strategy in flow cytometry to determine the frequency of ILC2s in lung leukocytes (CD45^{+}). Lineage markers were excluded (CD5^{-}, CD8a^{-}, CD19^{-}, FcεRIα^{-}, NK1.1^{-}, TCRγ/δ^{-}, Ter119^{-}). ILCs were CD45^{+} CD92.2^{hi} lin^{-}. Within these cells, ILC2s were defined as GATA3^{+} CD25^{+}. (d-e) Absolute numbers of (d) lung leukocytes and (e) ILC2s after enzymatic lung

dissociation. (f) Frequency of ILC2s in $CD45^{+}$ leukocytes 24 and 72 h post-sensitization and at day 18 of the acute asthma model. (a) 2 h, 4 h: n = 6; 24 h: n = 5 (b) *Tslp*: ctr: n = 7-8, WT HDM: n = 11, *Cd97*$^{-/-}$ HDM: n = 8, *Il33*: ctr: n = 6, WT HDM: n = 8, *Cd97*$^{-/-}$ HDM: n = 6 (d) 24 h: ctr n = 8-9, HDM: n = 11-12; 18 d: ctr n = 7-8, HDM: n = 10-11; (e-f) 24 h: ctr: n = 7, HDM: n = 10, 18 d: ctr: n = 4, HDM: n = 5-6 mice/group. In figures where data are presented as box-and-whisker plots, each data point represents an individual mouse, the centre line indicates the median, the box represents the IQR, and whiskers extend from minimum to the maximum. For statistical analysis, 2-sided Mann-Whitney U test was used.

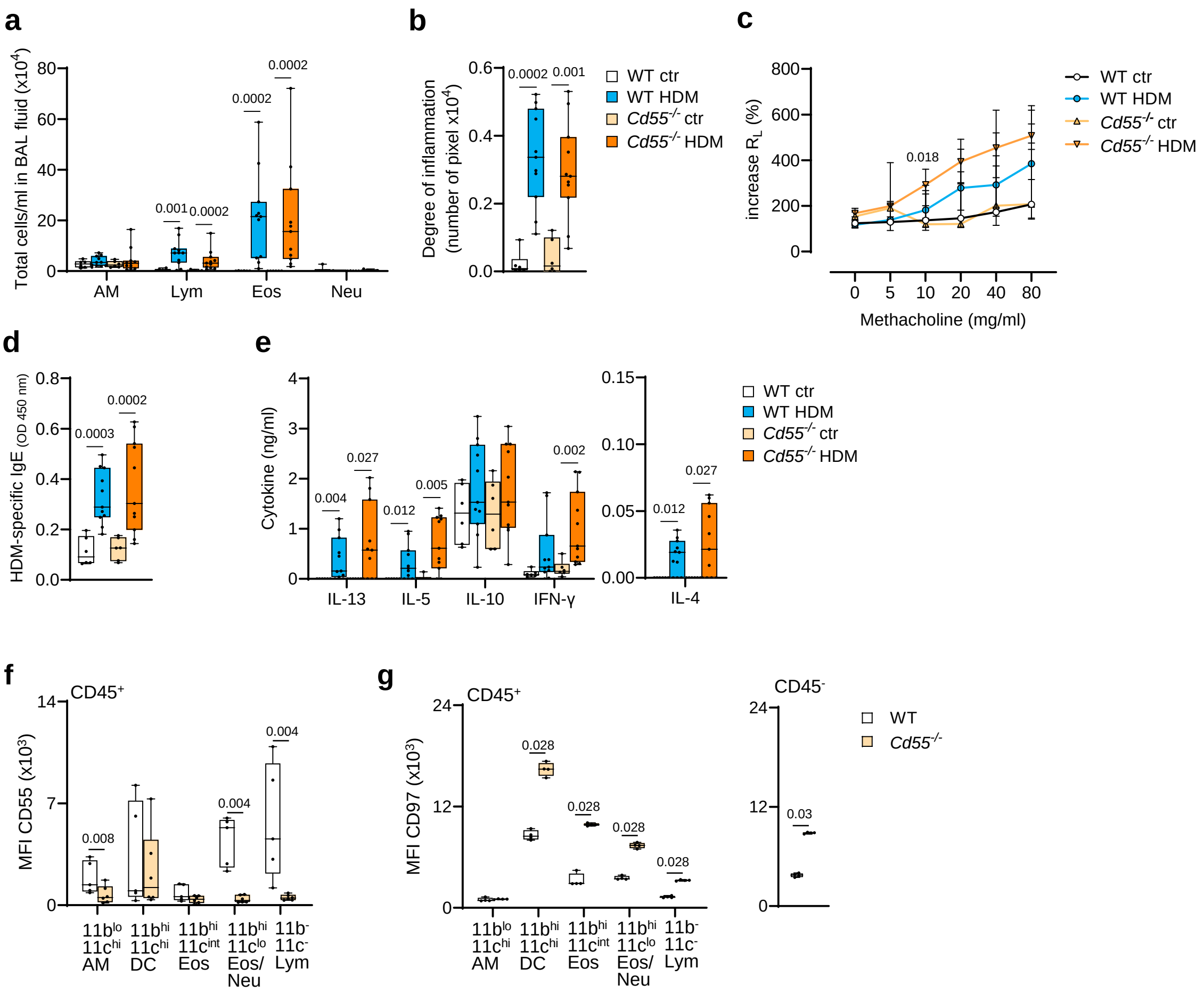

Supplementary Fig. 4. Depletion of *Cd55* has no effect on the allergic asthma phenotype. In the HDM-induced asthma model (see Fig. 1k), the asthma phenotype of WT and *Cd55*$^{-/-}$ mice was assessed by (a) cell numbers in the BAL fluid, (b) lung inflammation, (c) lung resistance (AHR), (d) HDM-specific IgE, and (e) cytokine production in re-stimulated splenocytes. (f, g) Characterization of isolated lung cells of the *Cd55*$^{-/-}$ mouse by flow cytometry; for the gating strategy and designations, see Fig. S2c. Expression of (f) CD55 and (g) CD97 on lung leukocyte subsets and CD45^{-} cells (CD97 only). (a-e) WT ctr, *Cd55*$^{-/-}$ ctr: n = 6; WT HDM, *Cd55*$^{-/-}$ HDM: n = 11; (f) WT: n = 5; *Cd55*$^{-/-}$ n = 6 (g) n = 4 mice/group. In figures where data are presented as box-and-whisker plots, each data point represents an individual mouse, the centre line indicates the median, the box represents the IQR, and whiskers extend from minimum to the maximum. Lung resistance data (c) are presented as median with IQR. For statistical analysis, 2-sided Mann-Whitney U test was used.

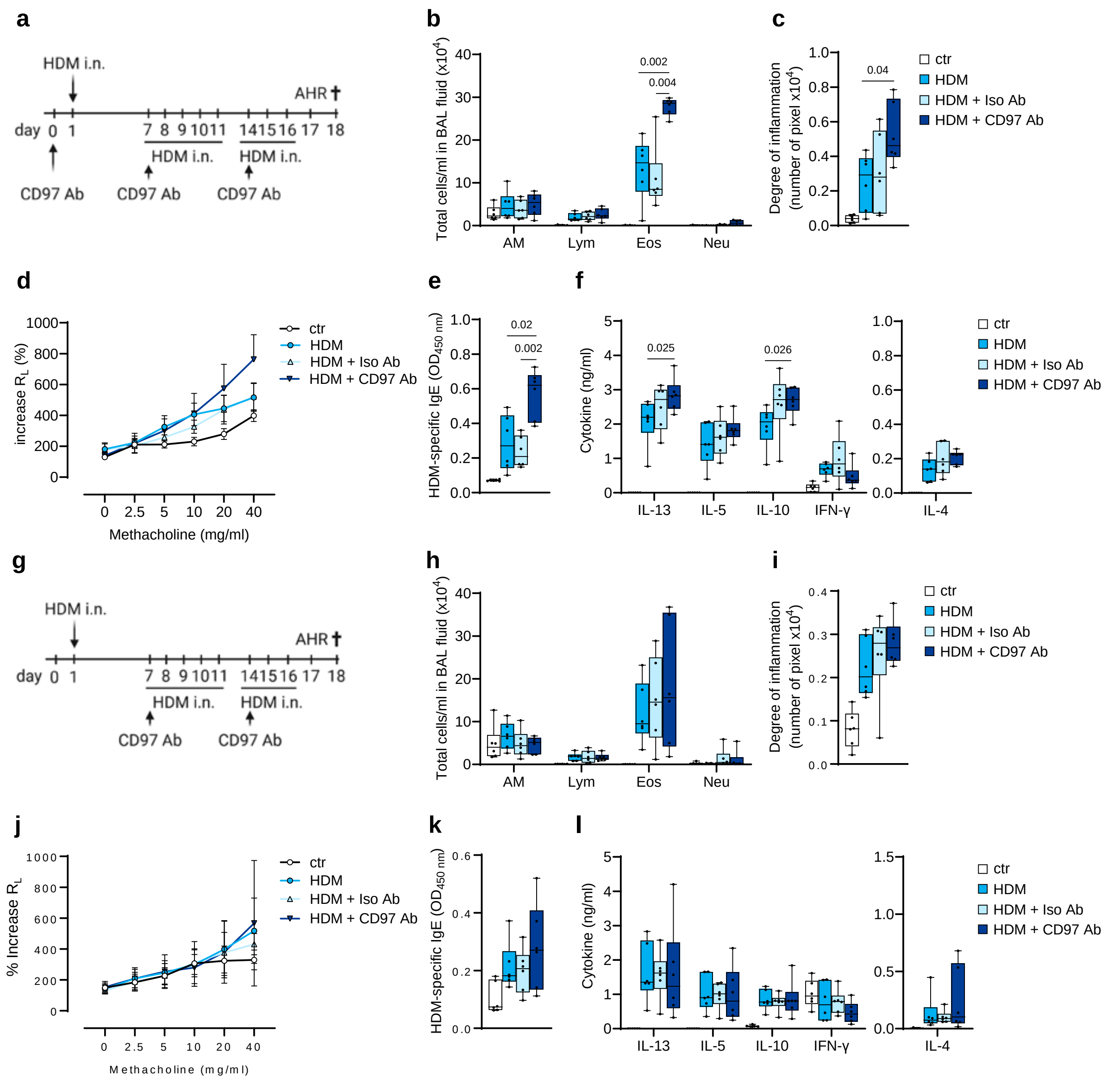

Supplementary Fig. 5. Effect of CD97 Ab application at different time points on asthma development.

(a) Protocol; the Abs were applied on days 0, 7 and 14 of the acute HDM model. (b) Total cell numbers in BAL fluid, (c) inflammation of lung tissues, (d) lung resistance (AHR) (e) HDM-specific IgE and (f) cytokine release of restimulated spleen cells of CD97 Ab- compared to isotype Ab-treated Balb/c mice were determined. (g) In another protocol Abs were applied on days 7 and 14. (h) Total cell numbers in BAL fluid, (i) inflammation of lung tissues, (j) lung resistance, (AHR), (k) HDM-specific IgE, and (l) cytokine production of restimulated spleen cells of CD97 Ab- compared to isotype Ab-treated Balb/c mice. Data of two independent experiments, n = 6 mice/group. In figures where data are presented as box-and-whisker plots, each data point represents an individual mouse, the centre line indicates the median, the box represents the IQR, and whiskers extend from minimum to the maximum. Lung resistance data (d, j) are presented as median with IQR. For statistical analysis, 2-sided Mann-Whitney U test was used.

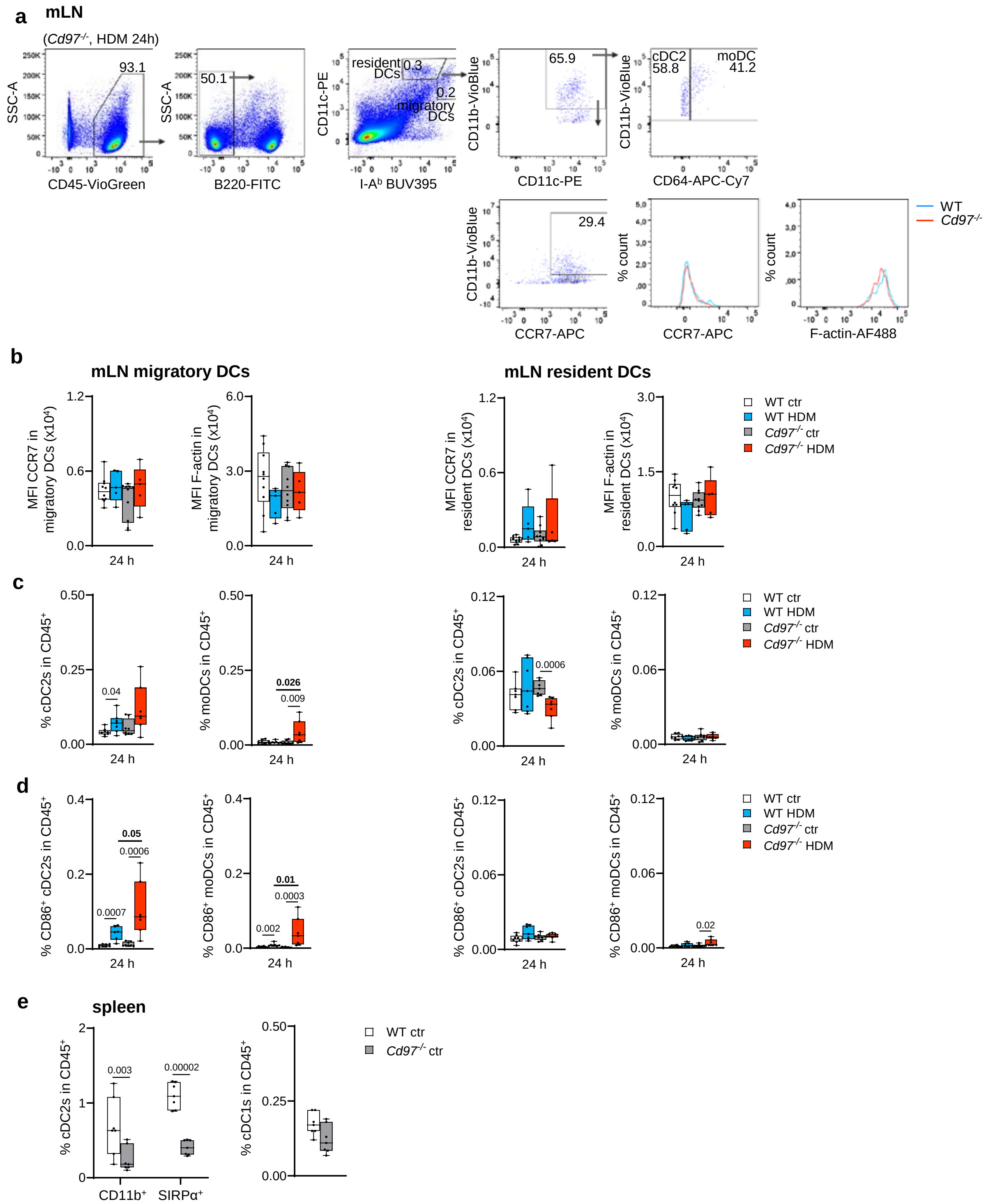

Supplementary Fig. 6. DCs in mLNs and spleen of WT and *Cd97$^{-/-}$* mice

(**a-c**) DCs in mLNs of naïve mice and 24 h post-sensitization. (**a**) Flow cytometry gating strategy for the characterization of DCs among mLN leukocytes ($CD45^{+}$). Within non-B cells ($CD45R/B220^{-}$) cells, two DC subsets were distinguished based on CD11c and I-A^{b} (MHC II) expression: migratory ($CD11c^{hi\text{-}int}$ I-$A^{b\ hi}$) and resident ($CD11c^{hi}$ I-$A^{b\ int}$). (**b**) CCR7 and F-actin MFI of resident and migratory mLN $CD11b^{+}$ DCs (consisting of cDC2s and moDCs). (**c, d**) Frequency of (**c**) all and of (**d**) activated resident and migratory cDC2s and moDCs among mLN leukocytes (**e**) Frequency of cDC2s (defined by $CD11b^{+}$ or

SIRPa⁺) and cDC1s among spleen leukocytes in naïve mice. moDCs, which also express CD11b⁺, are rare or essentially absent in spleen of naïve mice. (**b**) ctr: n = 10, HDM: n = 5; (**c-d**) ctr: n = 8; HDM: n = 7; (**e**) n = 7 mice/group. In figures where data are presented as box-and-whisker plots, each data point represents an individual mouse, the centre line indicates the median, the box represents the IQR, and whiskers extend from minimum to the maximum. For statistical analysis, 2-sided Mann-Whitney U test was used.

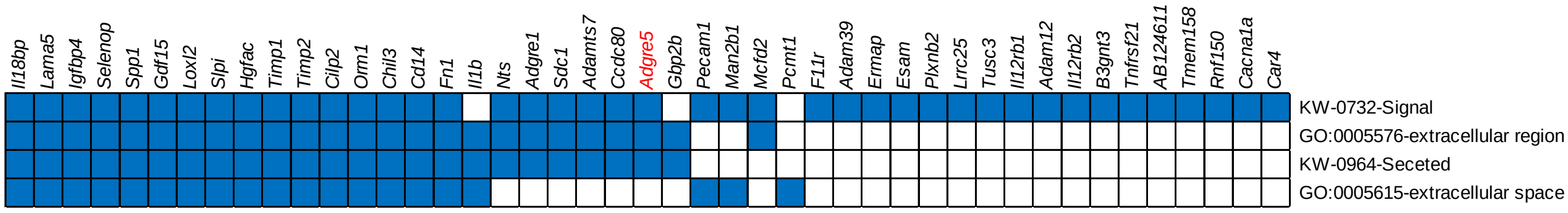

Supplementary Fig. 7. Gene set enrichment analysis of differentially expressed genes between WT and *Cd97*$^{-/-}$ CD11c^{+} OVA-pulsed BMDCs

A total of 153 differentially expressed genes (Supplementary Data 1) were analyzed using the Database for Annotation, Visualization, and Integrated Discovery (DAVID; https://davidbioinformatics.nih.gov). The displayed cluster of four Gene Ontology (GO) and UniProt annotation terms (vertical labels) was highly significantly enriched (DAVID enrichment score = 3.48). A subset of 45 differentially expressed genes is shown. These genes are associated with at least one of the four enriched annotation terms, as indicated by blue squares.

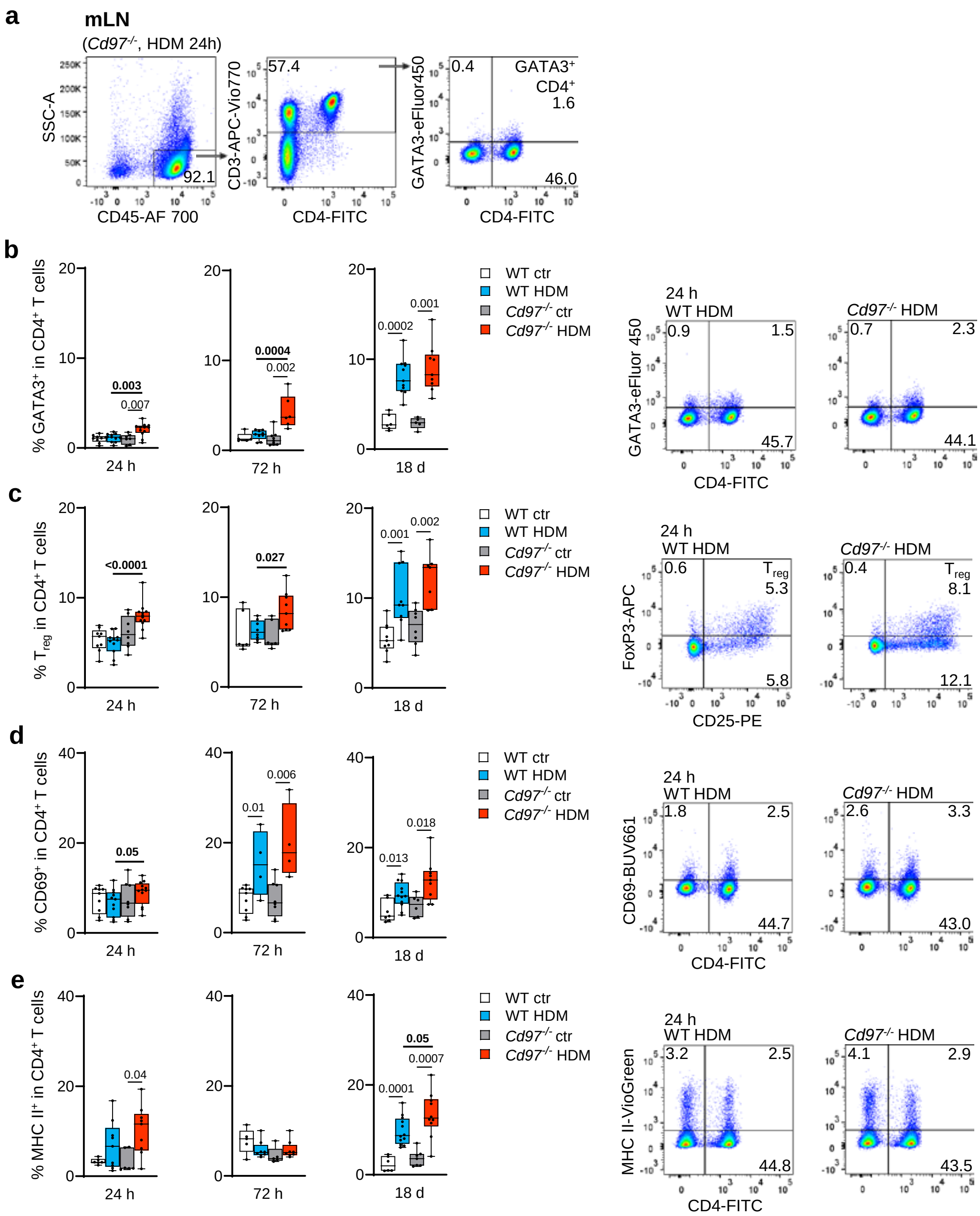

Supplementary Fig. 8 GATA3⁺ cells, T_{regs} and activated cells in mLN CD4⁺ T cells

At 24 and 72 h after HDM sensitization and on day 18 of the acute HDM-asthma model (protocol Fig. 4b), the frequencies of GATA3⁺ cells, T_{regs} (FoxP3⁺ CD25⁺) and activated cells among CD4⁺ T cells were quantified by flow cytometric. (**a**) Gating strategy. Frequency of (**b**) GATA3⁺, (**c**) T_{regs}, (**d**) CD69⁺, and (**e**) MHC II⁺ cells among CD4⁺ T cells. Right panels: Representative dot blots of an HDM-treated *Cd97*⁻/⁻ mouse 24 h post-sensitization. (**b**) 24 h: ctr: n = 7-9, HDM: n = 11-12; 72 h: ctr, WT HDM: n = 9; *Cd97*⁻/⁻ HDM: n = 6; 18 d: ctr: n = 6, HDM: n = 10-11; (**c**) 24 h: ctr: n = 9, HDM: n = 11; 72 h: ctr: n = 6, HDM: n = 8-9; 18 d: ctr: n = 9-10, HDM: n = 7-9; (**d**) 24 h: ctr: n = 8-9, HDM: n = 11; 72 h: ctr: n = 9, HDM: n = 5; 18 d: ctr: n = 6-7, HDM: n = 9-12; (**e**) 24 h: ctr: n = 6, HDM: n = 9; 72 h: ctr: n = 5, HDM: n = 8; 18 d: ctr: n = 6, HDM: n = 10-12 mice/group. In figures wheredata are presented as box-and-whisker plots, each data point represents an individual mouse, the centre line indicates the median, the box represents the IQR, and whiskers extend from minimum to the maximum. For statistical analysis, 2-sided Mann-Whitney U test was used.

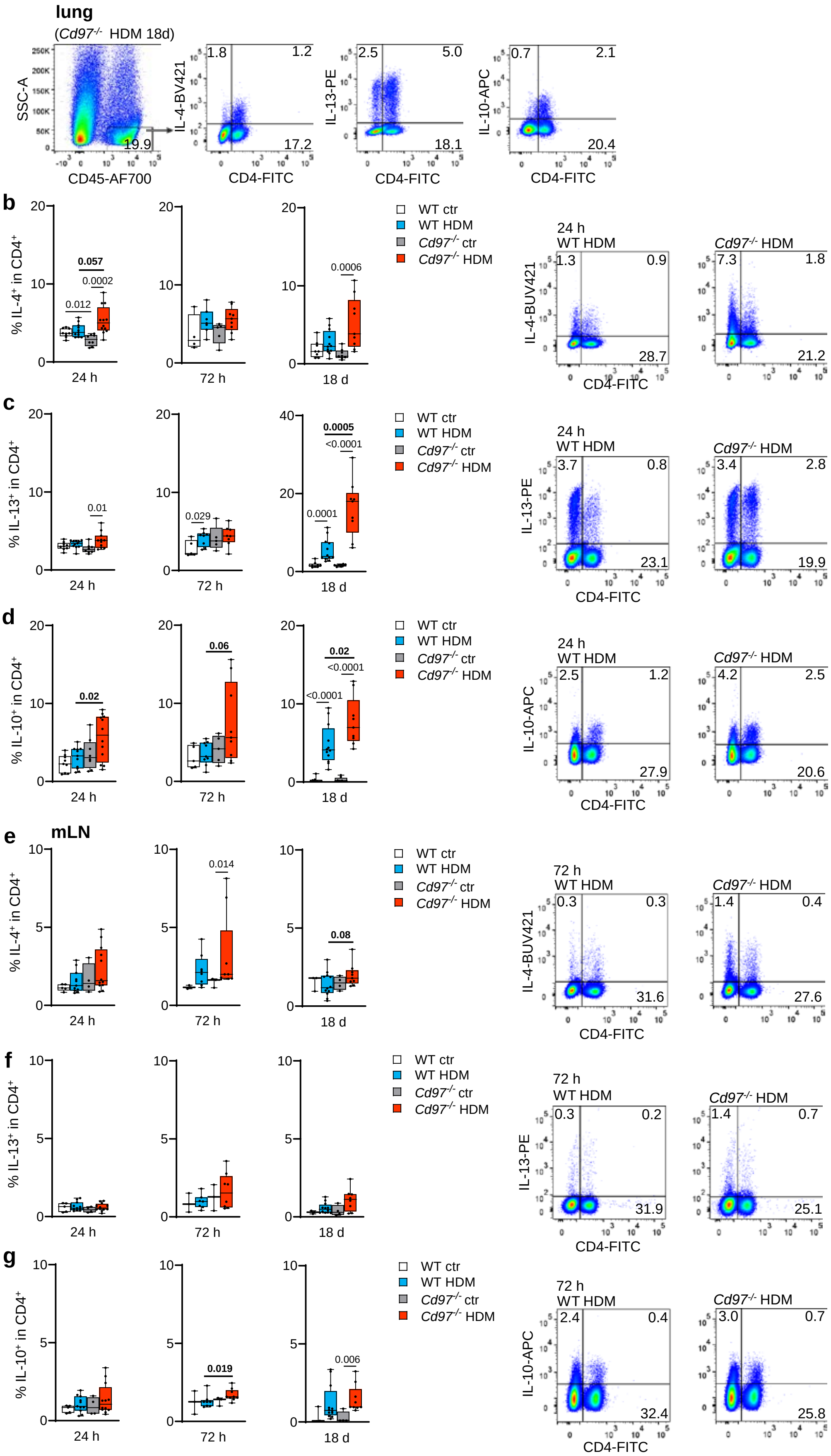
lung
(Cd97-/- HDM 18d)
SSC-A
CD45-AF700
IL-4-BV421
IL-13-PE
IL-10-APC
CD4-FITC
b
c
d
e
mLN
f
g
% IL-4+ in CD4+
% IL-13+ in CD4+
% IL-10+ in CD4+
24 h
72 h
18 d
WT ctr
WT HDM
Cd97-/- ctr
Cd97-/- HDM
24 h WT HDM
72 h WT HDM
IL-4-BUV421

Supplementary Fig. 9. Intracellular cytokine expression in restimulated lung and mLN lymphocytes

Isolated lung and mLN cells, isolated 24 and 72 h post-sensitization, and on day 18 of the acute HDM-model (protocol see Fig. 4b) were restimulated with PMA/ionomycin for 4 h in the presence of brefeldin A. The cells were CD4 surface-stained, intracellularly stained for cytokines, and analyzed in flow cytometry; (**a**) gating strategy. Frequency of (**b, e**) IL-4$^+$, (**c, f**) IL-13$^+$, and (**d, g**) IL-10$^+$ cells among (**b-d**) CD4$^+$ lung and (**e-g**) mLN lymphocytes. Right panels: Representative dot plots from mice 24 h post-sensitization. (**b-d**) 24 h: ctr: n = 8-9, HDM: n = 11-12; 72 h: ctr: n = 5, HDM: n = 8-9; 18 d: ctr: n = 8, HDM: n = 9-12; (**e-g**) 24 h: ctr: n = 4-5, HDM: n = 11-12; 72 h: ctr: n = 3, HDM: n = 8-9; 18 d: ctr: n = 3-4, HDM: n = 9-12 mice/group. In figures where data are presented as box-and-whisker plots, each data point represents an individual mouse, the centre line indicates the median, the box represents the IQR, and whiskers extend from minimum to the maximum. For statistical analysis, 2-sided Mann-Whitney U test was used.

Supplementary Methods

RNA-sequencing (RNA-seq) and Gene Ontology (GO) analysis of $CD11c^+$ BMDCs

A total 2 x 10^6 enriched $CD11c^+$ BMDCs were pulsed with 200 µg/ml OVA for 22 h (WT and *Cd97*$^{-/-}$, n=4 each). Total RNA was isolated using TRIzol (Thermo Fisher), and RNA quality was assessed on a Fragment Analyzer 5200 (Agilent Technilogies, Santa Clara, USA) using the High Sensitivity RNA quantification kit and Fragment Analyzer Controller Software (Agilent v 3.1.0.12). Random-primed libraries were prepared from 150 ng of total RNA using the Watchmaker RNA library prep kit with Polaris depletion (Watchmaker Genomics, Boulder, USA). Purified libraries were quantified by Qubit fluorometry (Thermo Fischer), and library size distribution was analysed on the Fragment Analyzer 5200. Sequencing of 2 x 150 bp was performed on an Illumina NovaSeq 6000 platform (Illumina, San Diego, USA). After demultiplexing with bcl2fastq software (Illumina, v2.20) and read polishing using FASTP [1], reads were aligned to the telomere-to-telomere mouse genome assembly (mhaESC v1.1 [2]) using HISAT2 [3]. Stringtie and the R package DESeq2 [4] were employed to collect and normalize read counts. DESeq2 was further used for differential expression analysis between WT and *Cd97*$^{-/-}$. A list of differentially expressed genes was filtered according to Benjamini-Hochberg adjusted p-values (padj) with a cut off below 0.01 and submitted to the Database for Annotation, Visualization, and Integrated Discovery (DAVID [5,6]) to discover enriched functional-related gene groups.

Supplementary Tables

Supplementary Table 1. Abs and dyes used in flow cytometry

Antigen/ target/dye	Fluorophore	Clone	Supplier	Cat. no.	Lot number	Dilution
Cell surface staining						
CD3	APC-Vio 770	REA641	Miltenyi	130-119-793	5230100154	1:50
CD4	FITC	RM4-4	BD	553055	9156647	1:400
CD4	PE-Vio 615	REA1211	Miltenyi	130-123-210	5200801140	1:50
CD11b	Vio Blue	REA592	Miltenyi	130-113-810	5251005876	1:50
CD11c	PE	REA754	Miltenyi	130-110-838	5220609685	1:100
CD25	PE	REA568	Miltenyi	130-120-766	5230403665	1:50
CD40	Vio Bright FITC	FGK45.5	Miltenyi	130-105-170	5250901649	1:10
CD45	VioGreen	REA737	Miltenyi	130-110-665	5220405520	1:50
CD45	Alexa Fluor 700	30-F11	Thermo	56-0451-82	2211060	1:400
CD45R/B220	FITC	REA755/RA3-6B2	Miltenyi	130-110-845	5251200239	1:50
CD64	APC-Vio 770	REA286	Miltenyi	130-118-685	5231105213	1:50
CD69	BUV661	H1.2F3	BD	741478	3221370	1:33
CD80	PE-Vio770	REA983	Miltenyi	130-116-462	5250904544	1:150
CD86	APC	REA1190	Miltenyi	130-122-130	5251100915	1:50
CD90.2	BUV395	53-2.1	BD	565257	1174425	1:33
CD97v2	Vio Bright FITC	REA678	Miltenyi	130-110-230	5201008276	1:10
CCR7*	APC	REA685	Miltenyi	130-126-307	5251003041	1:50
MHC II	PerCP-Vio 700	REA813	Miltenyi	130-112-234	5220405533	1:50
MHC II	VioGreen	REA813	Miltenyi	130-112-395	5230306677	1:50
MHC II I-A^{b}	BUV395	25-9-17	BD	745580	5337335	1:100
Siglec F	BUV395	E50-2440	BD	740280	1287475	1:100
SIRPa/ CD172a	PE-Vio 770	REA1201	Miltenyi	130-123-154	5250804207	1:50
XCR1	Vio Bright FITC	REA707	Miltenyi	130-111-378	5250804191	1:50
Lineage panel**						
CD5	Biotin	REA421	Miltenyi	130-106-238	5220808338	1:50
CD8a	Biotin	53-6.7	Miltenyi	130-118-147	5220703239	1:50
CD19	Biotin	REA749	Miltenyi	130-112-034	5230306671	1:50
FcεRIα	Biotin	MAR-1	Miltenyi	130-101-874	5220405563	1:50
NK1.1	Biotin	REA1162	Miltenyi	130-120-513	5220703244	1:50
TCRγ/δ	Biotin	REA633	Miltenyi	130-109-795	5220808342	1:10
Ter119	Biotin	Ter119	Miltenyi	130-120-828	5220703247	1:10
Biotin	PerCP	REA746	Miltenyi	130-110-960	5221001674	1:33
Intracellular staining***						
GATA3	eFluor 450	TWAJ	Thermo	48-9966-42	2731837	1:20
FoxP3	APC	REA788	Miltenyi	130-111-679	5230306295	1:50
IL-4	BV421	11B11	BioLegend	504119	B408057	1:20
IL-10	APC	JES5-16E3	BD	554468	1040473	1:200
IL-13	PE	W17010B	BioLegend	159403	B345441	1:200
F-actin staining****						
phalloidin	Alexa Fluor 488		Merck	A12379	808465	0.125 U/mL

Abs have been used in different combinations;
* CCR7 Ab incubation at 37ºC, 30 min;
** to exclude lineage markers for ILC analysis; detection using the Biotin-PerCP Ab;
*** re-add the CD4 Ab during intracellular staining;
**** final F-actin labelling with phalloidin-Atto488 according to manufacturer’s protocol

Supplementary Table 2. Primers for qRT-PCR

Gene	Primer sequence 5’ to 3’		NCBI reference sequence
	Forward	Reverse	
Rps29	atgggtcaccagcagctcta	gcctatgtccttcgcgtact	NM_009093.3
Il4	ggtctcaacccccagctagt	gccgatgatctctctcaagtgat	NM_021283.2
Il5	tcaccgagctctgttgacaa	ccacacttctctttttggcg	NM_010558.1
Il10	atttgaattccctgggtgagaag	cacaggggagaaatcgatgaca	NM_010548.2
Il13	acacaagaccagactcccct	ctcattagaaggggccgtgg	NM_008355.3
Il33	ggatgggaagaagctgatggtg	tccgaggactttttgtgaaggac	NM_001164724.2
Ifng	gaggaactggcaaaaggatggt	gctgatggcctgattgtctttc	NM_008337.4
Tslp	gcgacagcatggttcttctc	tgctcgaacttagccccttt	NM_021367.2

Supplementary References

1. Chen, S., Zhou, Y., Chen, Y. & Gu, J. fastp: an ultra-fast all-in-one FASTQ preprocessor. *Bioinformatics.* **34,** i884-i890; 10.1093/bioinformatics/bty560 (2018).
2. Liu, J. *et al.* The complete telomere-to-telomere sequence of a mouse genome. *Science* **386,** 1141–1146; 10.1126/science.adq8191 (2024).
3. Kim, D., Langmead, B. & Salzberg, S. L. HISAT: a fast spliced aligner with low memory requirements. *Nat. Methods* **12,** 357–360; 10.1038/nmeth.3317 (2015).
4. Love, M. I., Huber, W. & Anders, S. Moderated estimation of fold change and dispersion for RNA-seq data with DESeq2. *Genome Biol.* **15,** 550; 10.1186/s13059-014-0550-8 (2014).
5. Sherman, B. T. *et al.* DAVID: a web server for functional enrichment analysis and functional annotation of gene lists (2021 update). *Nucleic Acids Res* **50,** W216-W221; 10.1093/nar/gkac194 (2022).
6. Huang, d. W., Sherman, B. T. & Lempicki, R. A. Systematic and integrative analysis of large gene lists using DAVID bioinformatics resources. *Nat. Protoc.* **4,** 44–57 (2009).
7. Hornsteiner, F., Sykora, M. M., Tripp, C. H., Sopper, S. & Stoitzner, P. Mouse dendritic cells and other myeloid subtypes in healthy lymph nodes and skin: 26-Color flow cytometry panel for immune phenotyping. *Eur J Immunol* **52,** 2006–2009; 10.1002/eji.202250004 (2022).
