# Supplementary material for: CD97/ADGRE5 attenuates the induction of adaptive type 2 immune responses in allergic asthma
Source: Nat Commun. 2026 Aug 28;17:9226. doi: 10.1038/s41467-026-76948-9 (PMC13522487; doi:10.1038/s41467-026-76948-9)
Supplement: Supplementary file 2 [file 41467_2026_76948_MOESM2_ESM.pdf]

Description of Additional Supplementary Files

Supplementary Data 1: Differentially expressed genes in CD11c+ BMDCs of WT and Cd97-/- mice
