# Supplementary material for: CD97/ADGRE5 attenuates the induction of adaptive type 2 immune responses in allergic asthma
Source: Nat Commun. 2026 Aug 28;17:9226. doi: 10.1038/s41467-026-76948-9 (PMC13522487; doi:10.1038/s41467-026-76948-9)
Supplement: Supplementary file 3 [file 41467_2026_76948_MOESM4_ESM.pdf]

nature portfolio

Corresponding author(s): Tobias Polte

Last updated by author(s): Jul 22, 2026

Reporting Summary

Nature Portfolio wishes to improve the reproducibility of the work that we publish. This form provides structure for consistency and transparency in reporting. For further information on Nature Portfolio policies, see our Editorial Policies and the Editorial Policy Checklist.

Statistics

For all statistical analyses, confirm that the following items are present in the figure legend, table legend, main text, or Methods section.

n/a	Confirmed	
☐	☒	The exact sample size (*n*) for each experimental group/condition, given as a discrete number and unit of measurement
☐	☒	A statement on whether measurements were taken from distinct samples or whether the same sample was measured repeatedly
☐	☒	The statistical test(s) used AND whether they are one- or two-sided<br>*Only common tests should be described solely by name; describe more complex techniques in the Methods section.*
☒	☐	A description of all covariates tested
☐	☒	A description of any assumptions or corrections, such as tests of normality and adjustment for multiple comparisons
☐	☒	A full description of the statistical parameters including central tendency (e.g. means) or other basic estimates (e.g. regression coefficient) AND variation (e.g. standard deviation) or associated estimates of uncertainty (e.g. confidence intervals)
☐	☒	For null hypothesis testing, the test statistic (e.g. *F*, *t*, *r*) with confidence intervals, effect sizes, degrees of freedom and *P* value noted<br>*Give P values as exact values whenever suitable.*
☒	☐	For Bayesian analysis, information on the choice of priors and Markov chain Monte Carlo settings
☒	☐	For hierarchical and complex designs, identification of the appropriate level for tests and full reporting of outcomes
☒	☐	Estimates of effect sizes (e.g. Cohen's *d*, Pearson's *r*), indicating how they were calculated

Our web collection on statistics for biologists contains articles on many of the points above.

Software and code

Policy information about availability of computer code

Data collection	*Provide a description of all commercial, open source and custom code used to collect the data in this study, specifying the version used OR state that no software was used.*
Data analysis	Mouse data were analyzed using GraphPad Prism10.2. Gene expression comparisons between disease groups and experimental conditions for the human data were performed on pseudobulk count matrices using the DESeq2 package (v1.32.0, R v4.1.0). Differentially expressed genes were filtered according to Benjamini-Hochberg adjusted p-values (padj) with a cut off below 0.01 and submitted to the Database for Annotation, Visualization, and Integrated Discovery (DAVID 5,6) to discover enriched functional-related gene groups.

For manuscripts utilizing custom algorithms or software that are central to the research but not yet described in published literature, software must be made available to editors and reviewers. We strongly encourage code deposition in a community repository (e.g. GitHub). See the Nature Portfolio guidelines for submitting code & software for further information.

Data

Policy information about availability of data

All manuscripts must include a data availability statement. This statement should provide the following information, where applicable:

- Accession codes, unique identifiers, or web links for publicly available datasets
- A description of any restrictions on data availability
- For clinical datasets or third party data, please ensure that the statement adheres to our policy

Mouse RNA-seq raw data, metadata and processed data have been deposited in the GEO database (under accession number GSE339436).

Human scRNA-seq count matrices and related data are deposited in the GEO database (under accession number GSE193816), and raw human sequencing data are available in the controlled access repository dbGaP (www.ncbi.nlm.nih.gov/gap/) under accession number phs003101.v1.p1. Source code for data analysis in each main figure is available on GitHub (https://github.com/villani-lab/airway_allergic_asthma) and has been archived to Zenodo (https://zenodo.org/badge/latestdoi/412260708). A user-friendly portal is available to browse the single-cell data generated in this manuscript. Users can query the single-cell clustering solutions, visualize gene expression, and browse differential expression of genes. Users may navigate the portal by visiting https://villani.mgh.harvard.edu/allergy-asthma. All other relevant raw data from figures generated in this study have been provided in the Source Data file.

Research involving human participants, their data, or biological material

Policy information about studies with human participants or human data. See also policy information about sex, gender (identity/presentation), and sexual orientation and race, ethnicity and racism.

Reporting on sex and gender	The human study in this manuscript is from a nonrandomized, nonblinded mechanistic study with the primary aim of comparing the airway mucosal response to allergen challenge in allergic asthmatics and allergic non-asthmatic controls. The study protocol was approved by the Massachusetts General Brigham Institutional Review Board (IRB# 2007P001050) and registered at clinicaltrials.gov (NCT00595491). A full list of participants has been previously published in Science Immunology 5 May 2023 Vol 8, Issue 83 DOI: 10.1126/sciimmunol.abq6352 (Compiled data file S1). We recruited both males and females (self-reported) for the study, but sex was not analyzed as a biological variable. For the single cell RNA sequencing 8 participants were analyzed, four allergic asthmatics (AA) and four allergic non-asthmatic controls (AC). All of the AC subjects were female, while the the AA subjects studied included 3 females and 1 male.
Reporting on race, ethnicity, or other socially relevant groupings	We collected race and ethnicity data for the participants. Three of the AC subjects were white and the 4th was Latino. Three of the AA subjects were white and the 4th was black.
Population characteristics	This has been previously published in in Science Immunology 5 May 2023 Vol 8, Issue 83 DOI: 10.1126/sciimmunol.abq6352 (Compiled data file S1).
Recruitment	Subjects were recruited via posters placed in the MBTA in Boston
Ethics oversight	The study protocol was approved by the Massachusetts General Brigham Institutional Review Board (IRB# 2007P001050).

Note that full information on the approval of the study protocol must also be provided in the manuscript.

Field-specific reporting

Please select the one below that is the best fit for your research. If you are not sure, read the appropriate sections before making your selection.

[x] Life sciences [ ] Behavioural & social sciences [ ] Ecological, evolutionary & environmental sciences

For a reference copy of the document with all sections, see nature.com/documents/nr-reporting-summary-flat.pdf

Life sciences study design

All studies must disclose on these points even when the disclosure is negative.

Sample size	For mouse experiments an analysis with G-Power (3.1) was performed based on values received from earlier studies with a similar approach.
Data exclusions	No mice were excluded from the analysis.
Replication	Mouse experiments included groups of 3-6 mice/cage and were performed at least two times. Ex vivo/in vitro experiments were performed 4-8 times.
Randomization	Mice of one genotype were randomly allocated to the treatment groups.
Blinding	In the mouse study, tissue and cell processing was performed blinded to genotype and treatment, with mice identified by numeric codes only.

Reporting for specific materials, systems and methods

We require information from authors about some types of materials, experimental systems and methods used in many studies. Here, indicate whether each material, system or method listed is relevant to your study. If you are not sure if a list item applies to your research, read the appropriate section before selecting a response.

Materials & experimental systems

n/a	Involved in the study	
	x	Antibodies
x		Eukaryotic cell lines
x		Palaeontology and archaeology
	x	Animals and other organisms
	x	Clinical data
x		Dual use research of concern
x		Plants

Methods

n/a	Involved in the study	
		ChIP-seq
	x	Flow cytometry
		MRI-based neuroimaging

Antibodies

Antibodies used

Antigen, Fluorophore, Clone, Supplier, Cat. no., Lot number,Dilution:
Cell surface staining
CD3 APC-Vio 770, REA641, Miltenyi, 130-119-793, 5230100154, 1:50
CD4 FITC, RM4-4, BD, 553055, 9156647, 1:400
CD4 PE-Vio615, REA1211, Miltenyi 130-123-210, 5200801140, 1:50
CD11b VioBlue, REA592, Miltenyi, 130-113-810, 5251005876, 1:50
CD11c PE, REA754, Miltenyi, 130-110-838, 5220609685, 1:100
CD25 PE, REA568, Miltenyi, 130-120-766, 5230403665, 1:50
CD40 VioBright FITC, FGK45.5, Miltenyi 130-105-170, 5250901649, 1:10
CD45 VioGreen, REA737, Miltenyi, 130-110-665, 5220405520, 1:50
CD45 Alexa Fluor 700, 30-F11, Thermo, 56-0451-82, 2211060, 1:400
CD45R/B220 FITC, REA755/RA3-6B2, Miltenyi, 130-110-845, 5251200239, 1:50
CD64 APC-Vio770, REA286, Miltenyi, 130-118-685, 5231105213, 1:50
CD69 BUV661, H1.2F3, BD, 741478, 3221370, 1:33
CD80 PE-Vio770, REA983, Miltenyi, 130-116-462, 5250904544, 1:150
CD86 APC, REA1190, Miltenyi,130-122-130, 5251100915, 1:50
CD90.2 BUV395, 53-2.1, BD 565257, 1174425, 1:33
CD97v2 VioBright FITC, REA678, Miltenyi, 130-110-230, 5201008276, 1:10
CCR7 APC, REA685, Miltenyi, 130-126-307, 5251003041, 1:50
MHC II PerCP-Vio700, REA813, Miltenyi, 130-112-234, 5220405533, 1:50
MHC II VioGreen, REA813, Miltenyi, 130-112-395, 5230306677, 1:50
MHC II I-Ab BUV395, 25-9-17, BD, 745580, 5337335, 1:100
Siglec F BUV395, E50-2440, BD, 740280, 1287475, 1:100
SIRPa/ CD172a PE-Vio770, REA1201, Miltenyi, 130-123-154, 5250804207, 1:50
XCR1 Vio Bright FITC, REA707, Miltenyi, 130-111-378, 5250804191, 1:50
Lineage panel
CD5 Biotin, REA421, Miltenyi, 130-106-238, 5220808338, 1:50
CD8a Biotin, 53-6.7, Miltenyi, 130-118-147, 5220703239, 1:50
CD19 Biotin, REA749, Miltenyi, 130-112-034 ,5230306671, 1:50
FcεRIα Biotin, MAR-1 Miltenyi, 130-101-874, 5220405563, 1:50
NK1.1 Biotin, REA1162, Miltenyi, 130-120-513, 5220703244, 1:50
TCRγ/δ Biotin, REA633, Miltenyi, 130-109-795, 5220808342, 1:10
Ter119 Biotin, Ter119, Miltenyi, 130-120-828, 5220703247, 1:10
Biotin PerCP, REA746, Miltenyi, 130-110-960 ,5221001674, 1:33
Intracellular staining
GATA3 eFluor 450, TWAJ, Thermo, 48-9966-42, 2731837, 1:20
FoxP3 APC, REA788, Miltenyi, 130-111-679, 5230306295, 1:50
IL-4 BV421, 11B11, BioLegend, 504119, B408057, 1:20
IL-10 APC, JES5-16E3, BD, 554468, 1040473, 1:200
IL-13 PE, W17010B, BioLegend, 159403, B345441, 1:200

Validation

The CD97 and CD55 antibodies were validated by comparing the staining patterns of isolated splenocytes and lung-derived cells from wild-type mice with those from their respective knockout counterparts (Cd97-/-, Cd55-/-). All other antibodies are commercially available and were not validated by us.

Animals and other research organisms

Policy information about studies involving animals; ARRIVE guidelines recommended for reporting animal research, and Sex and Gender in Research

Laboratory animals	Species, mouse strains, sex and age are described in METHODS. C57BL/6 Cd97-/- and the respective wild-type (WT) mice were characterized for tissue-specific CD97 expression. To attribute the CD97-dependent effects in asthma to a certain cell type, we generated a Cd97loxP/loxP mouse in which exons 7-9 of Cd97 are flanked by loxP sites. By crossing this mouse line with a cell type-specific Cre-recombinase mouse, we can generate conditional Cd97-/- mice. C57BL/6J-Tg(Nkx2-1-cre)2Sand/J and C57B were obtained from The Jackson Laboratory (Bar Harbor, US). Female BALB/cByJ mice, 6-8 weeks of age, were obtained from the Elevage Janvier Laboratory (Le Genest St Isle, France) with a 7 day adaption period before the start of experiments. Furthermore, we used Cd55-/- mice, formerly designated as Daf1-/-, and their respective WT mice.
Wild animals	The study did not involve wild animals/animals captured in the field.
Reporting on sex	Only female mice were investigated due to their well-described higher susceptibility in mouse asthma models.
Field-collected samples	The study did not involve samples collected from the field/from wild animals.
Ethics oversight	All mouse experiments were performed in accordance with the institutional and state guidelines, as approved by the Committee on Animal Welfare of Saxony, Germany (TVV 61/16, TVV 56/20, and TVV 38/21).

Note that full information on the approval of the study protocol must also be provided in the manuscript.

Clinical data

Policy information about clinical studies

All manuscripts should comply with the ICMJE guidelines for publication of clinical research and a completed CONSORT checklist must be included with all submissions.

Clinical trial registration	NCT00595491, registered at clinicaltrials.gov
Study protocol	The study protocol was registered at clinicaltrials.gov (NCT00595491) and has been published in Science Immunology 5 May 2023 Vol 8, Issue 83 DOI: 10.1126/sciimmunol.abq6352. Male and female participants were included.
Data collection	Samples collected at Massachusetts General Hospital from 2018-2020
Outcomes	RNA expression in cells isolated from airway brushings

Plants

Seed stocks	*Report on the source of all seed stocks or other plant material used. If applicable, state the seed stock centre and catalogue number. If plant specimens were collected from the field, describe the collection location, date and sampling procedures.*
Novel plant genotypes	*Describe the methods by which all novel plant genotypes were produced. This includes those generated by transgenic approaches, gene editing, chemical/radiation-based mutagenesis and hybridization. For transgenic lines, describe the transformation method, the number of independent lines analyzed and the generation upon which experiments were performed. For gene-edited lines, describe the editor used, the endogenous sequence targeted for editing, the targeting guide RNA sequence (if applicable) and how the editor was applied.*
Authentication	*Describe any authentication procedures for each seed stock used or novel genotype generated. Describe any experiments used to assess the effect of a mutation and, where applicable, how potential secondary effects (e.g. second site T-DNA insertions, mosiacism, off-target gene editing) were examined.*

ChIP-seq

Data deposition

☐ Confirm that both raw and final processed data have been deposited in a public database such as GEO.

☐ Confirm that you have deposited or provided access to graph files (e.g. BED files) for the called peaks.

Data access links<br>*May remain private before publication.*	*For "Initial submission" or "Revised version" documents, provide reviewer access links. For your "Final submission" document, provide a link to the deposited data.*
Files in database submission	*Provide a list of all files available in the database submission.*
Genome browser session<br>(e.g. UCSC)	*Provide a link to an anonymized genome browser session for "Initial submission" and "Revised version" documents only, to enable peer review. Write "no longer applicable" for "Final submission" documents.*

Methodology

Replicates	*Describe the experimental replicates, specifying number, type and replicate agreement.*
Sequencing depth	*Describe the sequencing depth for each experiment, providing the total number of reads, uniquely mapped reads, length of reads and whether they were paired- or single-end.*
Antibodies	*Describe the antibodies used for the ChIP-seq experiments; as applicable, provide supplier name, catalog number, clone name, and lot number.*
Peak calling parameters	*Specify the command line program and parameters used for read mapping and peak calling, including the ChIP, control and index files used.*
Data quality	*Describe the methods used to ensure data quality in full detail, including how many peaks are at FDR 5% and above 5-fold enrichment.*
Software	*Describe the software used to collect and analyze the ChIP-seq data. For custom code that has been deposited into a community repository, provide accession details.*

Flow Cytometry

Plots

Confirm that:

- [x] The axis labels state the marker and fluorochrome used (e.g. CD4-FITC).
- [x] The axis scales are clearly visible. Include numbers along axes only for bottom left plot of group (a 'group' is an analysis of identical markers).
- [x] All plots are contour plots with outliers or pseudocolor plots.
- [x] A numerical value for number of cells or percentage (with statistics) is provided.

Methodology

Sample preparation	preparation of lung-derived cells: Lung tissue was placed on ice and the enzyme solution (Lung Dissociation Kit mouse, Miltenyi Biotec, Bergisch Gladbach, Germany) was injected gently into each lobe. The tissue was minced and subsequently dissociated using the gentleMACS Octo Dissociator with Heaters (Miltenyi; program 37C_m_LDK_1). The resulting cell suspension was passed through a 70 µm strainer and washed with FACS buffer (PBS pH 7.2, 0.5% BSA, 1 mM EDTA). Erythrocytes were lysed using the ammonium chloride-potassium lysis buffer (3 min, room temperature). After passing the cells through a 40 µm strainer and washing, they were stained for flow cytometric analysis.<br>The bronchoalveolar lavage (BAL) fluid was obtained post-euthanasia through a small incision in the trachea, followed by the insertion of a syringe to lavage the right lung thrice with 400 µl of phosphate-buffered saline.<br>Spleens were mashed through a 70 µm cell strainer in RPMI 1640/10% FCS using the plunger end of a syringe. The resulting cells were washed once with PBS. Erythrocytes were lysed in 2 mL ammonium chloride-potassium buffer for 2 min, followed by two PBS washes.<br>Lymph nodes (LNs) were manually collected under a stereo microscope, homogenized in RPMI 1640/10% FCS using a 0.1 mL micro tissue grinder (pestle clearance 0.1–0.15 mm) and washed once before use<br>Bone marrow (BM) cells were collected, depleted of red blood cells using ammonium chloride lysis puffer, and grown in RPMI1640 medium/10% FCS and 20 ng/ml rmGM-CSF for 7 days. CD11c+ cells were separated by using magnetic cell sorting systems and CD11c Microbeads (Milteny Biotec, Bergisch Gladbach, Germany). To induce BM-dendritic cell (DC) maturation, cultures were treated with OVA (200 µg/ml), LPS (10 ng/ml, Merck), recombinant mouse (rm) TNF (100 ng/ml) and rm sCD40L (1 µg/ml, both Immunotools, Friesoythe, Germany) for 18 h at day 7.
Instrument	BD LSRFortessaTM X-20 Cell Analyzer (BD Biosciences, Franklin Lakes, US)
Software	FlowJo Software 10 (FlowJo LLC, Ashland, US)
Cell population abundance	Cell population abundances are provided both in the gating strategy figures (showing one representative example) and in the respective figures presenting the data from all mice included in each analysis.
Gating strategy	Gating strategies have been added to the figures in both the main text and the supplementary file.

- [x] Tick this box to confirm that a figure exemplifying the gating strategy is provided in the Supplementary Information.

Magnetic resonance imaging

Experimental design

Design type	*Indicate task or resting state; event-related or block design.*

Design specifications | *Specify the number of blocks, trials or experimental units per session and/or subject, and specify the length of each trial or block (if trials are blocked) and interval between trials.*

Behavioral performance measures | *State number and/or type of variables recorded (e.g. correct button press, response time) and what statistics were used to establish that the subjects were performing the task as expected (e.g. mean, range, and/or standard deviation across subjects).*

Acquisition

Imaging type(s) | *Specify: functional, structural, diffusion, perfusion.*

Field strength | *Specify in Tesla*

Sequence & imaging parameters | *Specify the pulse sequence type (gradient echo, spin echo, etc.), imaging type (EPI, spiral, etc.), field of view, matrix size, slice thickness, orientation and TE/TR/flip angle.*

Area of acquisition | *State whether a whole brain scan was used OR define the area of acquisition, describing how the region was determined.*

Diffusion MRI ☐ Used ☐ Not used

Preprocessing

Preprocessing software | *Provide detail on software version and revision number and on specific parameters (model/functions, brain extraction, segmentation, smoothing kernel size, etc.).*

Normalization | *If data were normalized/standardized, describe the approach(es): specify linear or non-linear and define image types used for transformation OR indicate that data were not normalized and explain rationale for lack of normalization.*

Normalization template | *Describe the template used for normalization/transformation, specifying subject space or group standardized space (e.g. original Talairach, MNI305, ICBM152) OR indicate that the data were not normalized.*

Noise and artifact removal | *Describe your procedure(s) for artifact and structured noise removal, specifying motion parameters, tissue signals and physiological signals (heart rate, respiration).*

Volume censoring | *Define your software and/or method and criteria for volume censoring, and state the extent of such censoring.*

Statistical modeling & inference

Model type and settings | *Specify type (mass univariate, multivariate, RSA, predictive, etc.) and describe essential details of the model at the first and second levels (e.g. fixed, random or mixed effects; drift or auto-correlation).*

Effect(s) tested | *Define precise effect in terms of the task or stimulus conditions instead of psychological concepts and indicate whether ANOVA or factorial designs were used.*

Specify type of analysis: ☐ Whole brain ☐ ROI-based ☐ Both

Statistic type for inference | *Specify voxel-wise or cluster-wise and report all relevant parameters for cluster-wise methods.*

(See Eklund et al. 2016)

Correction | *Describe the type of correction and how it is obtained for multiple comparisons (e.g. FWE, FDR, permutation or Monte Carlo).*

Models & analysis

n/a	Involved in the study
☐	☐ Functional and/or effective connectivity
☐	☐ Graph analysis
☐	☐ Multivariate modeling or predictive analysis

Functional and/or effective connectivity | *Report the measures of dependence used and the model details (e.g. Pearson correlation, partial correlation, mutual information).*

Graph analysis | *Report the dependent variable and connectivity measure, specifying weighted graph or binarized graph, subject- or group-level, and the global and/or node summaries used (e.g. clustering coefficient, efficiency, etc.).*

Multivariate modeling and predictive analysis | *Specify independent variables, features extraction and dimension reduction, model, training and evaluation metrics.*
