# Supplementary material for: CD97/ADGRE5 attenuates the induction of adaptive type 2 immune responses in allergic asthma
Source: Nat Commun. 2026 Aug 28;17:9226. doi: 10.1038/s41467-026-76948-9 (PMC13522487; doi:10.1038/s41467-026-76948-9)
Supplement: Supplementary file 4 [file 41467_2026_76948_MOESM5_ESM.pdf]

nature portfolio

Peer Review File

CD97/ADGRE5 attenuates the induction of adaptive type 2 immune responses in allergic asthma

Corresponding Author: Dr Tobias Polte

This file contains all reviewer reports in order by version, followed by all author rebuttals in order by version.

Version 0:

Reviewer comments:

Reviewer #1

(Remarks to the Author)
This manuscript described the role of CD97/ADGRE5 on immune responses in allergic asthma and correlated findings to samples from human data. The manuscript presents several mechanisms of altering CD97, genetic knockout, cell-specific knockout, and antibody neutralization with consistent findings. Moreover the findings are are shown to not be strain dependent (C57BL/6 and BALB/c were used). Evidence is given for human samples from control and those with allergic asthma showing strong correlation between phenotype and CD97 expression levels. Overall, this is an interesting manuscript. Some items need to be addressed.

Main points:
• Line 130. Consider the comment on the chronic model. OVA model is IP OVA/ALUM then OVA in. The chronic 8 week OVA may show less inflammation in C57BL/6 due to development of tolerance (REF). Figure S1 may indicate that the loss of CD97 does not allow for as much tolerance to occur in B6, rather than promoting "more" inflammation or exacerbation of inflammation. Different mechanisms may be at play.
• Line 136. It is noted loss of CD97 on bronchial cells had "no impact" on asthma phenotype nor TSLP or IL-33 (Fig 2). The error bar on Fig 1B CD97-/- is quite large suggesting insufficient power? Also the degree of inflammation (2C) is quite lower as well as are IL-13 levels (F). What statistics did not find significance for IL-13 or degree of inflammation in these figures. Moreover, TSLP and IL-33 are usually measured at the protein level in BAL fluid at 1-4 hr time points post allergen exposure due to their quick release and function in the lung. Both 24 hours and mRNA may not be sufficient (particularly as IL-13 was so low, a key downstream cytokine of these chemokines). It is unclear if there was sufficient power and correct timing to make the statement of no change.Regardless, it does appear that the increased inflammation in CD97 full knockout is represented in the CSFR1-Cre CD97 mice indicating it is an immune population that leads to that phenotype.
• Line 152 is confusing. It is noted Alveolar macrophage, eosinophils, neutrophils, lymphocytes were CD55 negative (are you referring to the WT mice or knockout mice as it would be anticipated) as Fig 3G does show CD55 on WT eosinophil/neutrophils and lymphocytes. (clear bar). To be clear, it reads that CD55 is actually two genes in mice, thus explaining the reason AM still had some antibody recognition of CD55 in CD55 KO mice?
• Section line 188. It would be ideal to show activated DCs in lung draining lymph nodes to parallel the lung data, to see if there is an increase after sensitization (more DCs in DLN) in CD97KO mice. (Fig 5 shows the flow pattern for DLN but does not do the same profiling of the DCS, and is more broad (CD4 + or -). For this reason as well, line 215 is not as clear as DCs were not specifically stained for in Figure 6. This also brings into question kinetics on the "simultaneous" changes in T cells and DCs although it is noted in discussion this needs further analysis.
• Line 201 and Line 257. It is noted ILC2 were lower in CD97 KO HDM at all time points. Yet WT HDM did not have more ILC2s, which is a well published event in the lung of HDM challenged mice. Please explain. This makes it difficult to interpret the drop in ILC2s (below control PBS mice)- Does this mean there is an intrinsic deficiency in the ILC2s in CD97 KO mice? Line 257 Use of the word unlikely softens the statement, but consider interpretation.

Minor comments:
• The only introduction of CD97 Is that it is highly expressed on immune cells and epithelial cells and acts as a GPCR. Would it be helpful to provide a sentence on the potential functions that are noted in Ref 10 'adhesion, migration…'?
• Collagen assay is in main methods, but no data is shown there. Methods should move to supplement where collagen is shown in fig 1h.
• Line 225, no isotype control for IHC is shown for mouse and human lung to indicate no non-specific binding, particularly as

this antibody is staining a large number of cells in the lung.
• Line 258. ILC2 are leukocytes (all white blood cells are leukocytes) technical on gramma.
• Reference for “morphological criteria” (50, 51) seem odd as they are not about cell morphology/staining techniques but about epigenetic modifications and syndecan-4 in allergic inflammation. Particularly as reference 51 is also used for whole lung histological morphometric imaging analysis. Does this mean the BAL cell differentials were done by automated image analysis. It appears reference 51 then references a 15, 16… so on. Please reference appropriate primary reference for the technique.

Reviewer #2

(Remarks to the Author)
In the current manuscript Quaas et al examined the impact of CD97 deficiency on the development of allergic asthma using multiple models. The authors demonstrate that CD97 KO mice develop more robust BAL eosinophilia, lung inflammation, IgE prodiction, and Th2 cytokine (Il-5/IL-13) production following exposure to both OVA or HDM (both acute and chronic), compared to WT mice. Selective depletion of CD97 on structural lung cells (Nkx2.1 Cre-driven), or on leukocytes (Csf1R-Cre-driven) reveal that CD97 expression on leukocytes is responsible for driving the phenotype observed in whole body CD97-KO mice. Mice lacking CD55, one putative CD97 interacting partner, do not demonstrate increased severity of allergic asthma. Administration of blocking CD97 mAbs during the sensitization phase (but not during the challenge phase) was sufficient to increase severity of asthma. Blocking CD97 during sensitization did NOT alter ILC2 recruitment, or function, suggesting that DCs may be impacted. The authors demonstrate that CD97-/- BMDCs produce less IL-12 p70 and express more CD86 than WT DC following stimulation with OVA or LPS. Adoptive transfer of CD97 KO BMDCs to mice increased the development of eosinophilic inflammation and AHR compared to mice given WT BMDCs. The authors then demonstrate that HDM-treated CD97 KO mice have an increased frequency of CD86+ DCs in the lung after a single allergen exposure, and after the challenge phase, and that this is associated with an increased frequency of Th2 cells, and activated T cells during both the sensitization phase, and the challenge phase. The authors conclude by demonstrating that allergen challenge in human airways results in a significant decrease in CD97 expression on lung mononuclear phagocyte populations in individuals with asthma more strongly than in individuals without asthma. Thus, the authors conclude that CD97 plays a unique negative regulatory role in asthma through targeting of DC populations.

Major comments:

My chief concern is that the manuscript is overall very descriptive. There are several potential avenues to explore in this regard: Although they show that CD55 is NOT involved, there are several other partners CD97 has been described to interact with. Are one of them involved?

Alternatively, really digging into the role of CD97 in DCs could be a more mechanistic angle to pursue - Is CD97 regulating DC migration? Differentiation? Capacity to skew T cells? There is at least some evidence that CD97 regulates the balance of CDC1 versus CDC2 (PMID 35143305) in mice. as cDC2 are thought to be important drivers of allergic responses, this might explain some of the authors observations. This could definitely be expanded upon - do the lungs/LN of CD97 KO mice have an increased frequency of cDC2 at baseline or after allergen challenge? Do more of these cDC2s get to the draining LNs? Do cDC2 purified from WT and CD97-/- have differential capacity to drive T cell differentiation (or transfer disease)? This would get at the idea of whether this is an intrinsic difference in DC functionality, or whether it is simply a “numbers” game (i.e. there are MORE cDC2s in CD97-/- mice).

Additional points: It would be helpful to show the gating strategies for the T cells, and DC populations in supplementary figures

Why is OVA-specific IgE presented as titer, whereas HDM-specific IgE is presented as O.D?

The authors indicate there is a “trend” towards increased AHR in whole body CD97-/- mice. Fig 1F really suggests there is absolutely no difference. The only figure I would say there is a “trend” in is Sup Fig 3c – in CD55 KO mice.

Cytokine production in mice challenged with BMDCs is markedly higher than that observed following in vivo allergen sensitization/challenge. This is likely because animals were also sensitized to proteins in bovine serum, and these cells were similarly stimulated in the ex vivo cultures (if I am understanding the set up correctly). As such, I don’t really “trust” the interpretation of the cytokine data in Fig 4H and 4I.

Fig 5 – there may be an increase in total lung DCs expressing CD86 in CD97-/- Ctr mice versus WT Ctr mice. It is unclear from the description whether this difference was tested for significance, or not.

Fig 6 – While they show an increase in GATA3+ T cells, it would also be helpful to examine the frequency of Rorgt, Foxp3 or Tbet+ T cells to determine whether this is specific for Th2 cells, or it also applies to Th1, Treg, and Th17 cells. Alternatively, assessing expression of multiple cytokines (IL-5, IL-13, IL-17A) by lung T cells in this context would be helpful.

I am uncertain what an MHC Class II+ CD4+ T cell actually is? While MHC Class II has been reported as a marker of activation in human t cells, to my knowledge, mouse CD4+ T cells do not typically express this marker. An explanation of what this indicates (and showing the gating strategy, including FMOs) would be appropriate in this regard.

Several Supplementary figure panels appear to be missing, or truncated – Supp Fig 1A, Supp Fig 4A, 4G

The introduction could provide more focused information that would help set the stage for the paper – the authors spend several sentences discussing the role of epithelial cells in asthma, and the expression of CD97 on lung epithelial cells. However, since the authors rule those out as an focus for the CD97 effects, this is unneeded. More helpful would be a description of the types of DCs that play a role in asthma and/or the known signaling pathways/biology of CD97.

Similarly, the discussion of this manuscript leaves a lot to be desired. There is little placing of the current results in the context of the wider literature, and there is little flow to the discussion. Relevant areas for discussion would include the expected effects of CD97 on DCs (differentiation, migration, function) and or putative “interacting partners” of CD97 that may be mediating their observed effects.

Reviewer #3

(Remarks to the Author)
In this manuscript, Quaas et. al. investigated the role of CD97 in the development and progression of allergic asthma. The research group used a CD97-deficient mouse strain and CD97-blocking antibody applications in the classical laboratory strain Balb/c for this purpose.
In addition, the role of CD97 on DC was characterized in in vitro and cell transfer experiments and its influence on the activation of T cells was investigated. To investigate whether CD97/ADGRE5 also plays a role in humans, allergic and non-allergic asthmatics were provoked with allergen and the expression of CD97 was analyzed before and after provocation.
In both the acute OVA and HDM-specific model of allergic airway disease, it was shown that CD97-deficient animals exhibit an increased inflammatory response in BAL and lung tissue, as well as more allergen-specific IgE in serum and increased concentrations of type 2 cytokines in ex vitro stimulated cells. This could also be confirmed to a large extent in the chronic model of allergic airway disease. Using two further transgenic models, it was demonstrated that the expression of CD97 on immune cells and not on epithelial cells is responsible for the observed effects.
They were also able to demonstrate that the classic interaction partner CD55 does not appear to be directly involved in CD97-mediated regulation.
The application of CD97 blocking antibodies confirmed the functional role of CD97 in non-transgenic mice and showed that CD97 appears to have an immunomodulatory effect, especially in the primary sensitization phase.
In vitro experiments showed that CD97 on DC could play an important role in their activability. After activation with allergen or the TLR4 ligand LPS, the cells showed a reduced release of the Th1-driving cytokine IL-12. When allergen was administered, an increased expression of the co-stimulatory CD86 was observed.
In vivo transfer experiments showed that DC induce an exacerbated asthma phenotype, exhibit an increased degree of activation and contribute to the activation of type 2 T cells.
To test whether CD97 also plays a role in humans, its expression was examined in human lung immune cells with and without allergen stimulation. The group showed that CD97 expression is reduced after allergen stimulation, particularly in cells from allergic asthmatics.
The group concludes from the results that CD97 plays an important role in the regulation of DC functions, plays an important role in the induction of immune responses or sensitization and that CD97 could thus play an important role in future diagnostic screenings.
The manuscript is clearly structured and comprehensibly written. Graphics are as clear as possible. The following points should be addressed for clearer presentation and better understanding:

• The description of the implementation of the segmental allergen challenge, the collection of the samples and the implementation and quality assurance of the human samples is very brief.
• PAS staining: If there were differences in the number of mucus-producing cells in the airways, an evaluation would be carried out in addition to the purely histological presentation.
• CD97 main function in the sensitization phase. The authors assume that CD97 mainly plays an important role in the sensitization phase. The experiments investigating this aspect are significantly smaller in comparison to other approaches. The following aspects should be investigated:
o In vitro: How are other messengers and surface molecules regulated besides Il12p70 and CD86. How do CD97-deficient DC react to other stimuli? How do CD97 deficient DC interact with T cells in vitro?
o In vivo: Is allergen uptake and migration of DC and their interaction with T cells also modulated in vivo?
• HDM model: This model is described as adjuvant-free, at the same time it is mentioned in the material and that the allergen extract contains LPS. Please indicate the batch of HDM.
• In line 138, reference is made to Supplementary Figure 1C,D, but this should be 2 C,D.
• Figure 5 describes the increased number of CD86 positive DC within the CD11b positive cells. In the figure, however, the increased proportion of cells within the population of CD45 positive cells is shown. To better assess an activation shift, I think it would be more useful to show the MFI of CD86 within the population of CD11c/MHCII/CD11b positive cDC, or if high and low are so clearly separated, the % of high from low expressing cells. In addition to the visualization, it would also be interesting to see whether the absolute numbers of cells were also increased.
• Flow cytometry: In addition to the description of the procedure and the antibodies, it would also be interesting to see the

gating strategies for detecting DC, T cells, etc.
• Figure 6A shows the increased proportion of GATA3 positive CD4 T cells, Figure 6 B the proportion of CD69 positive CD4 + /- T cells and Figure C the proportion of MHCII in CD4+/- T cells in relation to CD3 total T cells. In my opinion, this type of analysis distorts the message. The key message should be that more CD4 T cells differentiate into Th2 cells and that these are more strongly activated. Therefore, I think it makes sense to characterize the degree of activation and differentiation within the population of CD4 positive cells and not within the entirety of CD3 positive cells in which CD8 cells can also contribute to a percentage shift. In addition to the relative modulation, it would also be interesting to see whether the absolute numbers of the individual subpopulations are also influenced.
• Why were BALB/C mice used in the antibody experiments and not CD57BL/6, which is the background strain of the transgenic animals?
• Please briefly describe the purification of spleen and lymph node cells.
• In the discussion, more reference should be made to current relevant literature: e.g. CD97 promotes spleen dendritic cell homeostasis through the mechanosensing of red blood cells; DOI: 10.1126/science.abi5965

Version 1:

Reviewer comments:

Reviewer #1

(Remarks to the Author)
Concerns were adequately addressed

Reviewer #2

(Remarks to the Author)
The authors have addressed the concerns of this reviewer.

Reviewer #3

(Remarks to the Author)
The authors have addressed all comments in detail. The introduction, materials and methods, results, and discussion have been revised. However, the manuscript still remains highly descriptive. The data regarding the role of CD97 on DCs have been further strengthened. From my perspective, the following questions nevertheless remain open:
1. Is the primary regulatory mechanism mediated through DCs? What role does CD97 play on other immune cells? How is the receptor expressed under homeostatic versus inflammatory conditions?
2. Liu et al. (DOI: 10.1126/science.abi5965) describe CD97 as a regulator required for maintaining splenic homeostasis, with receptor deficiency leading to impaired antibacterial responses. How do the authors interpret these findings in the context of their own work?
3. Why does differential regulation of DCs occur in vitro, even though an adhesion receptor is differentially regulated?

Version 2:

Reviewer comments:

Reviewer #3

(Remarks to the Author)
The authors have addressed all of my questions.
Additional gene expression data have been included, demonstrating that differential regulation occurs in WT and CD97 deficient DCs. Multiple aspects indicate that CD97 on DCs could be responsible for the altered phenotype. In the discussion, the authors note that involvement of other cell types cannot be completely ruled out.

Open Access This Peer Review File is licensed under a Creative Commons Attribution 4.0 International License, which permits use, sharing, adaptation, distribution and reproduction in any medium or format, as long as you give appropriate credit to the original author(s) and the source, provide a link to the Creative Commons license, and indicate if changes were made.
In cases where reviewers are anonymous, credit should be given to 'Anonymous Referee' and the source.
The images or other third party material in this Peer Review File are included in the article's Creative Commons license, unless indicated otherwise in a credit line to the material. If material is not included in the article's Creative Commons license and your intended use is not permitted by statutory regulation or exceeds the permitted use, you will need to obtain permission directly from the copyright holder.
To view a copy of this license, visit https://creativecommons.org/licenses/by/4.0/

Answers to the reviewers' comments on the manuscript:

"CD97/ADGRE5 attenuates the induction of adaptive type 2 immune responses in allergic asthma"

We thank the reviewers for their helpful comments on our manuscript. We have addressed all the issues raised in the responses below and conducted additional experiments, the results of which are included in the revised manuscript. Additional data and changes to the manuscript are indicated in red. The Discussion has been completely revised.
We hope that we have clarified the remaining questions and appreciate your time and effort in reviewing our work.

Reviewer #1 (Remarks to the Author):

This manuscript described the role of CD97/ADGRE5 on immune responses in allergic asthma and correlated findings to samples from human data. The manuscript presents several mechanisms of altering CD97, genetic knockout, cell-specific knockout, and antibody neutralization with consistent findings. Moreover the findings are shown to not be strain dependent (C57BL/6 and BALB/c were used). Evidence is given for human samples from control and those with allergic asthma showing strong correlation between phenotype and CD97 expression levels. Overall, this is an interesting manuscript. Some items need to be addressed.

Main points
Comment 1: Line 130. Consider the comment on the chronic model. OVA model is IP OVA/ALUM then OVA in. The chronic 8 week OVA may show less inflammation in C57BL/6 due to development of tolerance (REF). Figure S1 may indicate that the loss of CD97 does not allow for as much tolerance to occur in B6, rather than promoting "more" inflammation or exacerbation of inflammation. Different mechanisms may be at play.

Response:
We appreciate the reviewer's thoughtful comments on the chronic asthma model. Although the OVA-induced chronic asthma model is well-established and widely used (reviewed in [1,2]), we agree with the reviewer that continuous OVA inhalation can induce immunological tolerance. This has been shown by reduced eosinophilic inflammation in the chronic compared to the acute model [3,4], and demonstrated directly [5,6]. Thus, we cannot exclude the possibility that CD97 counteract tolerance induction in the chronic model. However, we believe that other mechanisms may underlie the observed effects of CD97, as our chronic model is characterized by increased neutrophils in the BAL of *Cd97*$^{-/-}$ mice, suggesting a distinct inflammatory response. We now address this point in the revised Discussion.

Comment 2: Line 136. It is noted loss of CD97 on bronchial cells had "no impact" on asthma phenotype nor TSLP or IL-33 (Fig 2). The error bar on Fig 1(2)B CD97$^{-/-}$ is quite large suggesting insufficient power? Also the degree of inflammation (2C) is quite lower as well as are IL-13 levels (F). What statistics did not find significance for IL-13 or degree of inflammation in these figures.

Moreover, TSLP and IL-33 are usually measured at the protein level in BAL fluid at 1-4 hr time points post allergen exposure due to their quick release and function in the lung. Both 24 hours and mRNA may not be sufficient (particularly as IL-13 was so low, a key downstream cytokine of these chemokines). It is unclear if there was sufficient power and correct timing to make the statement of no change. Regardless, it does appear that the increased inflammation in CD97 full knockout is represented in the CSFR1-Cre CD97 mice indicating it is an immune population that leads to that phenotype.

Response:
We re-examined our *Nkx2.1*$^{Cre/?}$ *Cd97*$^{-/-}$ mouse data and identified missing values related to inflammation and cytokine measurements, which have now been included. We apologize for this mistake.

The objective of using *Nkx2.1*$^{Cre/?}$ *Cd97*$^{-/-}$ mice was to determine whether the deletion of CD97 in epithelial cells reproduces the *Cd97*$^{-/-}$ phenotype, which would indicate a key role of CD97 in epithelial cells. However, the results do not support this, as the conditional mice did not show an enhanced asthma

phenotype. Although increasing group sizes might reveal significant differences, this would likely correspond to a reduced asthma phenotype and would therefore further support the conclusion that epithelial cells do not contribute to the asthma-promoting effect observed after CD97 loss.

As noted by Reviewer, the timeline for detecting alarmins in lung samples was suboptimal. We therefore quantified IL-33 and TSLP protein in lung homogenates and BAL fluid from naive mice and at 2, 6, and 24 h after allergen exposure (Fig. S3a) to verify immediate allergen-induced release. No differences were observed between WT and *Cd97*$^{-/-}$ mice. The mRNA data indicate that later in the time course alarmins were newly synthesized, but WT and *Cd97*$^{-/-}$ mice again were similar.

Together, the results with *Nkx2.1*$^{Cre/?}$ *Cd97*$^{-/-}$ mice and the similar TSLP/IL-33 levels in *Cd97*$^{-/-}$ and WT mice indicate that epithelial cells are not critical for the asthma-attenuating effect of CD97. Accordingly, we revised the wording to "an asthma phenotype comparable to WT mice".

Comment 3: Line 152 is confusing. It is noted Alveolar macrophage, eosinophils, neutrophils, lymphocytes were CD55 negative (are you referring to the WT mice or knockout mice as it would be anticipated) as Fig S 3G does show CD55 on WT eosinophil/neutrophils and lymphocytes. (clear bar). To be clear, it reads that CD55 is actually two genes in mice, thus explaining the reason AM still had some antibody recognition of CD55 in CD55 KO mice?

Response:
The observed CD55 staining in lung cDCs of *Cd55*$^{-/-}$ mice is most likely attributable to the second gene encoding mouse CD55/DAF, *Cd55b*, which remains intact in these mice [7]. Notably, also Liu et al. targeted only *Cd55* [8]. We have revised this point for clarity.

Comment 4: Section line 188. It would be ideal to show activated DCs in lung draining lymph nodes to parallel the lung data, to see if there is an increase after sensitization (more DCs in DLN) in CD97KO mice. (Fig 5 shows the flow pattern for DLN lung but does not do the same profiling of the DCS, and is more broad (CD4 + or -). For this reason, as well, line 215 is not as clear as DCs were not specifically stained for in Figure 6. This also brings into question kinetics on the "simultaneous" changes in T cells and DCs although it is noted in discussion this needs further analysis.

Response:
In the lungs and mediastinal lymph nodes (mLNs), we now performed a more detailed analysis of conventional type 2 dendritic cells (cDC2s) and monocyte-derived DCs (moDCs). Both subsets contribute to the establishment and maintenance of HDM-induced Th2 immunity, with cDC2s acting as potent inducers of Tfh2 cells in the mLNs [9–11]. Recently, it has been shown that HDM sensitization increases both the number and frequency of cDC2s and, in particular, moDCs in the lungs and mLNs [9].

We confirmed these findings and observed increased frequencies of cDC2s and (activated) moDCs exclusively in the lungs of *Cd97*$^{-/-}$ mice as early as 24 h after sensitization (Fig. 4). Concurrently, in the mLNs, the frequency of migratory (activated) moDCs was increased in *Cd97*$^{-/-}$ compared to WT mice (Fig. S6), whereas the frequencies of resident DC subsets remained unchanged in both genotypes. In the same time frame, *Cd97*$^{-/-}$ CD4^{+} T cells exhibited increased activation and upregulated GATA3 expression, particularly in the lungs (Fig. 7).

Comment 5: Line 201 and Line 257. It is noted ILC2 were lower in CD97 KO HDM at all time points. Yet WT HDM did not have more ILC2s, which is a well published event in the lung of HDM challenged mice. Please explain. This makes it difficult to interpret the drop in ILC2s (below control PBS mice)- Does this mean there is an intrinsic deficiency in the ILC2s in CD97 KO mice? Line 257 Use of the word unlikely softens the statement, but consider interpretation.

Response:
Our initial reference point for quantifying ILCs by flow cytometry was incorrect; the data are now related to lung leukocytes. As expected, WT mice showed increased ILC2 frequencies among lung leukocytes at 24 h after sensitization (Fig. S3f). In *Cd97*$^{-/-}$ mice no difference was observed between naïve and HDM-sensitized mice. At day 18, HDM-treated *Cd97*$^{-/-}$ mice displayed even lower ILC2 frequencies. Because the ILC2 frequencies were calculated relative to lung leukocytes, the marked leukocytic

infiltration in HDM-treated *Cd97*$^{-/-}$ mice (e.g. eosinophils and neutrophils) likely resulted in a relative reduction in ILC2 frequencies.

In contrast to the findings of Klein Wolterink et al. [12], we did not observe increased absolute ILC2 numbers in WT mice following HDM treatment (Fig. S3e). Several factors may account for this difference. First, different treatment protocols were used to induce allergic asthma. We applied a relatively mild protocol because CD97 deficiency resulted in worsened disease. Second, our antibody panel used to define ILC2s (CD45^{+} lin^{-} CD90^{+} GATA3^{+} CD25^{+}) differed from that used by Klein Wolterink et al., who defined ILC2s as CD45^{+} lin^{-} CD127^{+} T1/ST2^{+} CD25^{+}.

Minor points

1. The only introduction of CD97 Is that it is highly expressed on immune cells and epithelial cells and acts as a GPCR. Would it be helpful to provide a sentence on the potential functions that are noted in Ref 10 'adhesion, migration…'?

Response:
We added information on CD97 on structure, signaling, and function in the Introduction, including its well-defined signaling mechanism (induced by shedding of the CD97 N-terminal fragment by CD55). In the Discussion, we relate our results to published data on CD97 localization and its still unclear role in the T cell immunological synapse.

2. Collagen assay is in main methods, but no data is shown there. Methods should move to supplement where collagen is shown in fig 1h.

Response:
Thank you for this comment. However, we have decided against dividing the Methods section according to whether the results are presented in the main text or in the supplement, as this would not only affect the collagen assay but also other (partial) data. We believe that describing all methods in the main text is appropriate for interpreting all the results provided.

3. Line 225, no isotype control for IHC is shown for mouse and human lung to indicate no non-specific binding, particularly as this antibody is staining a large number of cells in the lung.

Response:
We included staining with an antibody directed against another adhesion GPCR, CELSR3/*ADGRC3* (from the same company and generated as part of the same Ab initiative as the CD97 Ab) as a negative control. Lung epithelial cells do not express *ADGRC3* (www.proteinatlas.org).

4. Line 258. ILC2 are leukocytes (all white blood cells are leukocytes) technical on gramma.

Response:
We have revised the wording.

5. Reference for "morphological criteria" (50, 51) seem odd as they are not about cell morphology/staining techniques but about epigenetic modifications and syndecan-4 in allergic inflammation. Particularly as reference 51 is also used for whole lung histological morphometric imaging analysis. Does this mean the BAL cell differentials were done by automated image analysis. It appears reference 51 then references a 15, 16… so on. Please reference appropriate primary reference for the technique.

Response:
As suggested by the reviewer we have removed the references mentioned and replaced them with the primary reference for the technique.

Reviewer #2 (Remarks to the Author):

In the current manuscript Quaas et al examined the impact of CD97 deficiency on the development of allergic asthma using multiple models. The authors demonstrate that CD97 KO mice develop more robust BAL eosinophilia, lung inflammation, IgE prodiction, and Th2 cytokine (Il-5/IL-13) production following exposure to both OVA or HDM (both acute and chronic), compared to WT mice. Selective depletion of CD97 on structural lung cells (Nkx2.1 Cre-driven), or on leukocytes (Csf1R-Cre-driven) reveal that CD97 expression on leukocytes is responsible for driving the phenotype observed in whole body CD97-KO mice. Mice lacking CD55, one putative CD97 interacting partner, do not demonstrate increased severity of allergic asthma. Administration of blocking CD97 mAbs during the sensitization phase (but not during the challenge phase) was sufficient to increase severity of asthma. Blocking CD97 during sensitization did NOT alter ILC2 recruitment, or function, suggesting that DCs may be impacted. The authors demonstrate that CD97-/- BMDCs produce less IL-12 p70 and express more CD86 than WT DC following stimulation with OVA or LPS. Adoptive transfer of CD97 KO BMDCs to mice increased the development of eosinophilic inflammation and AHR compared to mice given WT BMDCs. The authors then demonstrate that HDM-treated CD97 KO mice have an increased frequency of CD86+ DCs in the lung after a single allergen exposure, and after the challenge phase, and that this is associated with an increased frequency of Th2 cells, and activated T cells during both the sensitization phase, and the challenge phase. The authors conclude by demonstrating that allergen challenge in human airways results in a significant decrease in CD97 expression on lung mononuclear phagocyte populations in individuals with asthma more strongly than in individuals without asthma. Thus, the authors conclude that CD97 plays a unique negative regulatory role in asthma through targeting of DC populations.

Main points

Comment 1: My chief concern is that the manuscript is overall very descriptive. There are several potential avenues to explore in this regard: Although they show that CD55 is NOT involved, there are several other partners CD97 has been described to interact with. Are one of them involved?

Response:

Further studies are required to identify the CD97 interaction partner(s) mediating its effects in allergic asthma. Cell surface receptors CD55 and THY1 bind the CD97 NTF [13,14]. In our study, *Cd55* loss had no impact on asthma development. However, lung DCs still expressed CD55 likely from the *Cd55.b* gene, and *Cd55*$^{-/-}$ mice showed increased CD97 levels on lung immune cells. Therefore, we cannot completely exclude a potential role for the CD97-CD55 interaction in allergic asthma.

Previously, we investigated the role of THY1 in OVA-induced allergic asthma and observed reduced infiltration of eosinophils and monocytes in *Thy1*$^{-/-}$ mice [15], which makes it unlikely that THY1 is involved in mediating CD97 effects. Moreover, THY1 is abundantly expressed on T cells and DCs in mice, but not in humans [16], which limits the relevance of those findings to human pathology. Additionally, chondroitin-sulphate B (CS) [17], and integrins αvβ3 and α5β1 [18] interact with human CD97 on immune cells. However, murine CD97 lacks both the CS binding domain [19] and the integrin-binding site, precluding their investigation in our models.

We have added these arguments and data to the Discussion section.

Comment 2: Alternatively, really digging into the role of CD97 in DCs could be a more mechanistic angle to pursue - Is CD97 regulating DC migration? Differentiation? Capacity to skew T cells? There is at least some evidence that CD97 regulates the balance of CDC1 versus CDC2 (PMID 35143305) in mice as cDC2 are thought to be important drivers of allergic responses, this might explain some of the authors observations. This could definitely be expanded upon - do the lungs/LN of CD97 KO mice have an increased frequency of cDC2 at baseline or after allergen challenge? Do more of these cDC2s get to the draining LNs? Do cDC2 purified from WT and CD97-/- have differential capacity to drive T cell differentiation (or transfer disease)? This would get at the idea of whether this is an intrinsic difference in DC functionality, or whether it is simply a “numbers” game (i.e. there are MORE cDC2s in CD97-/- mice).

Response:

The attenuated asthma phenotype observed in CD97-deficient mice cannot be explained by altered lung or mLN DC numbers in naïve animals. The numbers and frequencies of total and activated cDC2s and moDCs in lungs and mLNs were similar in naïve WT and *Cd97*$^{-/-}$ mice (Fig. 4, S6). Likewise, peripheral LN cDC2s, as well as BM- and pre-splenic DC frequencies were comparable between WT and *Adgre5*$^{-}$

$^{-/-}$ mice at baseline, as reported previously [8]. In contrast, splenic cDC2 numbers were reduced in naïve *Adgre5*$^{-/-}$ mice [8], which we confirmed in our *Cd97*$^{-/-}$ mice (Fig. S6e). cDC2s localize in the splenic bridging channels, where the CD97 NTF is shed upon interaction with CD55 on circulating erythrocytes—a mechanism we previously described [20]. This promotes splenic retention of cDC2s, whereas in *Adgre5*$^{-/-}$ mice more cDC2s enter the circulation [8].
Lung and mLN DCs numbers are unaffected by CD97 deficiency, as DCs in these tissues do not contact circulating erythrocytes. We expanded the Discussion accordingly, incorporating findings from the published study [8]. Instead, 24 h after sensitization, *Cd97*$^{-/-}$ mice showed higher frequencies and a more activated phenotype of lung (and partly mLN) cDC2s and moDCs than WT mice, while absolute numbers of lung cDC1s were lower.

Our *in vitro* and *in vivo* BMDC data indicate an intrinsic functional difference between WT and *Cd97*$^{-/-}$ DCs. As suggested by the reviewer, we performed additional studies: functional assessment of BMDCs after stimulation, including activation markers, secreted cytokines, migratory capacity, and their ability to stimulate allergen-primed $CD4^+$ OT-II cells (Fig. 5).

Additional points:
1. It would be helpful to show the gating strategies for the T cells, and DC populations in supplementary figures.

Response:
Gating strategies have been added at the beginning of each section in which new flow cytometric analyses are presented.

2. Why is OVA-specific IgE presented as titer, whereas HDM-specific IgE is presented as O.D?

Response:
OVA-specific IgE is typically reported as a titer, reflecting the highest serum dilution that yields a positive signal, an approach suited to endpoint dilution assays commonly used in animal models with OVA as a defined antigen. In contrast, HDM-specific IgE is usually presented as optical density (O.D.), as it is measured by ELISA, where absorbance at a specific wavelength directly reflects Ab binding intensity.

3. The authors indicate there is a "trend" towards increased AHR in whole body CD97$^{-/-}$ mice. Fig 1F really suggests there is absolutely no difference. The only figure I would say there is a "trend" in is Sup Fig 3c – in CD55 KO mice.

Response:
To avoid any misinterpretations, we have removed the term "trend" from the manuscript and now describe the results strictly according to the statistical analysis.

4. Cytokine production in mice challenged with BMDCs is markedly higher than that observed following in vivo allergen sensitization/challenge. This is likely because animals were also sensitized to proteins in bovine serum, and these cells were similarly stimulated in the ex vivo cultures (if I am understanding the set up correctly). As such, I don't really "trust" the interpretation of the cytokine data in Fig 4H and 4I.

Response:
That is indeed an interesting observation. Prior to transfer, BMDCs are washed with PBS, resuspended in physiological saline (0.9% NaCl), and administered intrapulmonarily; thus, mice are not directly exposed to bovine serum proteins. However, BMDCs may be stimulated by serum components during *in vitro* differentiation, which could explain the elevated cytokine levels observed *in vivo*. Importantly, this effect applies equally to WT and *Cd97*$^{-/-}$ BMDCs and therefore does not account for the differences observed between them.

5. Fig 5 – there may be an increase in total lung DCs expressing CD86 in CD97$^{-/-}$ ctr mice versus WT ctr mice. It is unclear from the description whether this difference was tested for significance, or not.

Response:
We phenotyped lung and mLN DCs in greater detail, and we display all statistical significances between genotypes, treatment groups etc. in the figures. In naïve mice of both genotypes, the numbers and frequencies of DC subsets were comparable. Significant differences between WT and *Cd97*$^{-/-}$ emerged already after sensitization and often persisted until the end of the acute asthma model.

6. Fig 6 – While they show an increase in GATA3^{+} T cells, it would also be helpful to examine the frequency of Rorgt, Foxp3 or Tbet^{+} T cells to determine whether this is specific for Th2 cells, or it also applies to Th1, T_{reg}, and Th17 cells. Alternatively, assessing expression of multiple cytokines (IL-5, IL-13, IL-17A) by lung T cells in this context would be helpful.

Response:
We examined the frequency of lung and mLN T regulatory cells (T_{regs}; FoxP3^{+} CD25^{+}) 24 and 72 h post-sensitization, as well as on day 18 of the acute HDM model in WT and *Cd97*$^{-/-}$ mice (Fig. 7d, Fig. S7c) and quantified intracellular cytokine expression of *in vitro* restimulated CD4^{+} lung an mLN cells (Fig. S8). In this flow cytometry antibody panel, we included IL-4, IL-13 and IL-10. Unfortunately, IL-17 was not a focus at the time when we conducted these flow cytometric experiments.

7. I am uncertain what an MHC Class II^{+} CD4^{+} T cell actually is? While MHC Class II has been reported as a marker of activation in human t cells, to my knowledge, mouse CD4^{+} T cells do not typically express this marker. An explanation of what this indicates would be appropriate in this regard.

Response:
In humans, MHC II expression on T cells, a marker of activation (reviewed in [21]), is elevated on CD4^{+} T cells in severe asthma [22,23], and identifies recently divided antigen-specific effector CD4^{+} T cells [24], whereas in mice it is epigenetically silenced via CIITA repression [25,26]. Allergic asthma regulates epigenetically Th differentiation and memory [27], and other immune functions [28], likely enabling murine lung T cells to express MHC II after HDM treatment. We have added these notes to the Discussion.

We controlled this result carefully using the respective fluorescence minus one (FMO) control in flow cytometry. We added dot blots visualizing MHC II (with the respective control FMO) at lung T cells of HDM treated *Cd97*$^{-/-}$ mice (Fig. 7f).

8. Several Supplementary figure panels appear to be missing, or truncated – Supp Fig 1A, Supp Fig 4A, 4G

Response:
We have checked the numbering of all figures and corrected it accordingly.

9. The introduction could provide more focused information that would help set the stage for the paper – the authors spend several sentences discussing the role of epithelial cells in asthma, and the expression of CD97 on lung epithelial cells. However, since the authors rule those out as a focus for the CD97 effects, this is unneeded. More helpful would be a description of the types of DCs that play a role in asthma and/or the known signaling pathways/biology of CD97.

Response:
We have revised the introduction and provided more information on the types of DCs that play a role in allergic asthma, as well as on the structure, signaling and function of CD97.

10. Similarly, the discussion of this manuscript leaves a lot to be desired. There is little placing of the current results in the context of the wider literature, and there is little flow to the discussion. Relevant areas for discussion would include the expected effects of CD97 on DCs (differentiation, migration, function) and or putative "interacting partners" of CD97 that may be mediating their observed effects.

Response:

We have completely updated and revised the discussion and included additional information on the impact of CD97 on DC function, as well as its potential interaction partners.

Reviewer #3 (Remarks to the Author):

In this manuscript, Quaas et. al. investigated the role of CD97 in the development and progression of allergic asthma.
The research group used a CD97-deficient mouse strain and CD97-blocking antibody applications in the classical laboratory strain Balb/c for this purpose.
In addition, the role of CD97 on DC was characterized in in vitro and cell transfer experiments and its influence on the activation of T cells was investigated. To investigate whether CD97/ADGRE5 also plays a role in humans, allergic and non-allergic asthmatics were provoked with allergen and the expression of CD97 was analyzed before and after provocation.
In both the acute OVA and HDM-specific model of allergic airway disease, it was shown that CD97-deficient animals exhibit an increased inflammatory response in BAL and lung tissue, as well as more allergen-specific IgE in serum and increased concentrations of type 2 cytokines in ex vitro stimulated cells. This could also be confirmed to a large extent in the chronic model of allergic airway disease. Using two further transgenic models, it was demonstrated that the expression of CD97 on immune cells and not on epithelial cells is responsible for the observed effects.
They were also able to demonstrate that the classic interaction partner CD55 does not appear to be directly involved in CD97-mediated regulation.
The application of CD97 blocking antibodies confirmed the functional role of CD97 in non-transgenic mice and showed that CD97 appears to have an immunomodulatory effect, especially in the primary sensitization phase.
In vitro experiments showed that CD97 on DC could play an important role in their activability. After activation with allergen or the TLR4 ligand LPS, the cells showed a reduced release of the Th1-driving cytokine IL-12. When allergen was administered, an increased expression of the co-stimulatory CD86 was observed.
In vivo transfer experiments showed that DC induce an exacerbated asthma phenotype, exhibit an increased degree of activation and contribute to the activation of type 2 T cells.
To test whether CD97 also plays a role in humans, its expression was examined in human lung immune cells with and without allergen stimulation. The group showed that CD97 expression is reduced after allergen stimulation, particularly in cells from allergic asthmatics.
The group concludes from the results that CD97 plays an important role in the regulation of DC functions, plays an important role in the induction of immune responses or sensitization and that CD97 could thus play an important role in future diagnostic screenings.
The manuscript is clearly structured and comprehensibly written. Graphics are as clear as possible. The following points should be addressed for clearer presentation and better understanding:

Comment 1: The description of the implementation of the segmental allergen challenge, the collection of the samples and the implementation and quality assurance of the human samples is very brief.

Response:
We have added these methods in more detail. The detailed inclusion/exclusion criteria, sample collection, quality assurance, etc., have been previously published as cited [29].

Comment 2: PAS staining: If there were differences in the number of mucus-producing cells in the airways, an evaluation would be carried out in addition to the purely histological presentation.

Response:
We have added the results of a software-based evaluation of mucus production by goblet cells based on the number of pixels in PAS-stained lung sections (Fig. 1f).

Comment 3: CD97 main function in the sensitization phase. The authors assume that CD97 mainly plays an important role in the sensitization phase. The experiments investigating this aspect are significantly smaller in comparison to other approaches. The following aspects should be investigated:
a) In vitro: How are other messengers and surface molecules regulated besides Il12p70 and CD86. How do CD97-deficient DC react to other stimuli? How do CD97 deficient DC interact with T cells in vitro?
b) In vivo: Is allergen uptake and migration of DC and their interaction with T cells also modulated in vivo?

Response:
As suggested by the reviewer, we analyzed BMDCs activated with additional stimuli (TNFα, CD40L), assessing further activation markers (CD80, CD40) and cytokines (IL-1β, IL-6, IL-23, TNFα). OVA and

LPS increased CD86, CD80, and partly CD40 expression, while TNFα raised CD40 more in *Cd97*$^{-/-}$ BMDCs. Aside from differences in IL-12 release, cytokine secretion after stimulation was similar between *Cd97*$^{-/-}$ and WT BMDCs.
To evaluate DC–T cell interactions, OVA-pulsed CD11c^{+} BMDCs and lung cells from WT and *Cd97*$^{-/-}$ mice were co-cultured with CD4^{+} OT-II cells expressing an OVA-specific αβTCR. CD11c^{+} DCs from *Cd97*$^{-/-}$ mice induced stronger CD4^{+} OT-II proliferation and Th2 cytokine secretion (Fig. 5f, g). We performed adoptive transfer of allergen-primed BMDCs from both genotypes into naïve WT mice, followed by allergen challenge. *Cd97*$^{-/-}$ BMDCs induced a stronger allergic phenotype, supporting a proof-of-concept (Fig. 6).
Furthermore, 24 h after HDM sensitization, we observed an increased frequency of activated cDC2s and, particularly, moDCs exclusively in *Cd97*$^{-/-}$ mice. Both DC subsets play key roles in initiating and sustaining Th2-mediated immunity [9,10]. Concurrently, the frequency of GATA3^{+} and IL-4^{+} CD4^{+} T cells was increased in the lungs of *Cd97*$^{-/-}$ mice.
Both the *in vitro* and *in vivo* data support our assumption that CD97 plays a significant role in the sensitization phase.

Comment 4: HDM model: This model is described as adjuvant-free, at the same time it is mentioned in the material and that the allergen extract contains LPS. Please indicate the batch of HDM.

Response:
We have added the batch/LOT number of the HDM we used and corrected the information on endotoxin concentration. The 7.925 referred to the vial and not to ml.

Comment 5: In line 138, reference is made to Supplementary Figure 1C,D, but this should be 2 C,D. Control at the end

Response:
Thank you for pointing that out. We have corrected it.

Comment 6: Figure 5 (old) describes the increased number of CD86 positive DC within the CD11b positive cells. In the figure, however, the increased proportion of cells within the population of CD45 positive cells is shown. To better assess an activation shift, I think it would be more useful to show the MFI of CD86 within the population of CD11c/MHCII/CD11b positive cDC, or if high and low are so clearly separated, the % of high from low expressing cells. In addition to the visualization, it would also be interesting to see whether the absolute numbers of cells were also increased.

Response:
We have now added representative dot plots from flow cytometric analyses of lung cDC2s and moDCs, visualizing CD86 expression (Fig. 4j). The plots show clear separation of CD86^{-} and CD86^{+} cells within the DC subsets making the frequency more informative than CD86 MFI (which was similar among CD86^{+} cells). In contrast, *in vitro*-generated BMDCs stimulated with different agents display shifts in CD86 MFI without clear separation of CD86^{-} and CD86^{+} cells (Fig. 5a).

We also included absolute numbers of lung cDC2s, moDCs and cDC1s (Fig. 4 d, e). After sensitization, DC numbers were very low and increased only in in *Cd97*$^{-/-}$ mice. By day 18, absolute numbers had increased in both groups, but the increase was significantly greater in *Cd97*$^{-/-}$ mice than in WT mice. Because total cell yields after enzymatic lung digestion varied considerably, we additionally quantified absolute numbers of CD11c^{hi} MHC II^{hi} DCs (cDC2s, moDCs, cDC1s [30]) by immunostaining of lung sections and observed no difference between naïve WT and *Cd97*$^{-/-}$ mice (Fig. 4a). Absolute DC numbers in mLNs could not be provided, as they depend on the number of mLNs that can be isolated under the stereomicroscope, which is particularly challenging in naïve mice.

Comment 7: Flow cytometry: In addition to the description of the procedure and the antibodies, it would also be interesting to see the gating strategies for detecting DC, T cells, etc.

Response:
Gating strategies have been added at the beginning of each section in which new flow cytometric analyses are presented.

Comment 8: Figure 6A shows the increased proportion of GATA3 positive CD4 T cells, Figure 6 B the proportion of CD69 positive CD4 + /- T cells and Figure C the proportion of MHCII in CD4+/- T cells in relation to CD3 total T cells. In my opinion, this type of analysis distorts the message. The key message should be that more CD4 T cells differentiate into Th2 cells and that these are more strongly activated. Therefore, I think it makes sense to characterize the degree of activation and differentiation within the population of CD4 positive cells and not within the entirety of CD3 positive cells in which CD8 cells can also contribute to a percentage shift.

Response:
We now report the frequency of GATA3$^+$, CD69$^+$, or MHCII$^+$ cells within the CD4$^+$ and CD4$^-$ T cells in lung (Fig. 7) and mLNs (Fig. S7).

In addition to the relative modulation, it would also be interesting to see whether the absolute numbers of the individual subpopulations are also influenced.

Response:
The absolute number of lung (CD4$^+$) T cells was similar in naïve mice of both genotypes (Fig. 7b). HDM challenge increased these numbers similarly.

Comment 9: Why were BALB/C mice used in the antibody experiments and not CD57BL/6, which is the background strain of the transgenic animals?

Response:
BALB/c mice are commonly used in allergic asthma models due to their stronger Th2-biased immune response compared to C57BL/6 mice [31]. C57BL/6 mice, however, are the standard background for most transgenic lines and were the only available background for the *Cd97*$^{-/-}$ mice. Importantly, the use of two genetically distinct strains, both showing enhanced allergic responses upon CD97 deficiency, strengthens our conclusions regarding the role of CD97 in allergic asthma.

Comment 10: Please briefly describe the purification of spleen and lymph node cells.

Response: We have added a brief description of the purification of spleen and LN cells to the Methods section.

Comment 11: In the discussion, more reference should be made to current relevant literature: e.g. CD97 promotes spleen dendritic cell homeostasis through the mechanosensing of red blood cells; DOI: 10.1126/science.abi5965

Response: We have now discussed the role of CD97 in DC homeostasis in detail.

References

1. Nials, A. T. & Uddin, S. Mouse models of allergic asthma: acute and chronic allergen challenge. *Dis Model Mech.* **1,** 213–220; 10.1242/dmm.000323 (2008).
2. Woodrow, J. S., Sheats, M. K., Cooper, B. & Bayless, R. Asthma: the use of animal models and their translational utility. *Cells* **12**; 10.3390/cells12071091 (2023).
3. Fernandez-Rodriguez, S., Ford, W. R., Broadley, K. J. & Kidd, E. J. Establishing the phenotype in novel acute and chronic murine models of allergic asthma. *Int Immunopharmacol* **8,** 756–763; 10.1016/j.intimp.2008.01.025 (2008).
4. Pae, S. *et al.* Chronic allergen challenge induces bronchial mast cell accumulation in BALB/c but not C57BL/6 mice and is independent of IL-9. *Immunogenetics* **62,** 499–506; 10.1007/s00251-010-0452-1 (2010).
5. Sakai, K., Yokoyama, A., Kohno, N., Hamada, H. & Hiwada, K. Prolonged antigen exposure ameliorates airway inflammation but not remodeling in a mouse model of bronchial asthma. *Int Arch Allergy Immunol* **126,** 126–134; 10.1159/000049503 (2001).
6. Schramm, C. M. *et al.* Chronic inhaled ovalbumin exposure induces antigen-dependent but not antigen-specific inhalational tolerance in a murine model of allergic airway disease. *Am. J. Pathol.* **164,** 295–304; 10.1016/S0002-9440(10)63119-7 (2004).
7. Lin, F. *et al.* Tissue distribution of products of the mouse decay-accelerating factor (DAF) genes. Exploitation of a Daf1 knock-out mouse and site-specific monoclonal antibodies. *Immunology.* **104,** 215–225 (2001).
8. Liu, D. *et al.* CD97 promotes spleen dendritic cell homeostasis through the mechanosensing of red blood cells. *Science* **375,** eabi5965; 10.1126/science.abi5965 (2022).
9. Plantinga, M. *et al.* Conventional and monocyte-derived CD11b(+) dendritic cells initiate and maintain T helper 2 cell-mediated immunity to house dust mite allergen. *Immunity* **38,** 322–335; 10.1016/j.immuni.2012.10.016 (2013).
10. Sakurai, S. *et al.* Conventional type 2 lung dendritic cells are potent inducers of follicular helper T cells in the asthmatic lung. *Allergol Int* **70,** 351–359; 10.1016/j.alit.2021.01.008 (2021).
11. Olsthoorn, S. E. M., van Krimpen, A., Hendriks, R. W. & Stadhouders, R. Chronic inflammation in asthma: looking beyond the Th2 Cell. *Immunol. Rev.* **330,** e70010; 10.1111/imr.70010 (2025).
12. Klein Wolterink, Roel G J *et al.* Pulmonary innate lymphoid cells are major producers of IL-5 and IL-13 in murine models of allergic asthma. *Eur J Immunol* **42,** 1106–1116; 10.1002/eji.201142018 (2012).
13. Hamann, J. *et al.* Characterization of the CD55 (DAF)-binding site on the seven-span transmembrane receptor CD97. *Eur. J Immunol.* **28,** 1701–1707 (1998).
14. Wandel, E., Saalbach, A., Sittig, D., Gebhardt, C. & Aust, G. Thy-1 (CD90) is an interaction partner for CD97 on activated endothelial cells. *J. Immunol.* **188,** 1442–1450 (2012).
15. Schubert, K. *et al.* Thy-1 (CD90) regulates the extravasation of leukocytes during inflammation. *Eur. J. Immunol.* **41,** 645–656 (2011).
16. Mestas, J. & Hughes, C. C. W. Of mice and not men: differences between mouse and human immunology. *J Immunol* **172,** 2731–2738; 10.4049/jimmunol.172.5.2731 (2004).
17. Stacey, M. *et al.* The epidermal growth factor-like domains of the human EMR2 receptor mediate cell attachment through chondroitin sulphate glycosaminoglycans. *Blood* **102,** 2916–2924 (2003).
18. Wang, T. *et al.* CD97, an adhesion receptor on inflammatory cells, stimulates angiogenesis through binding integrin counter receptors on endothelial cells. *Blood* **105,** 2836–2844 (2004).
19. Kwakkenbos, M. J. *et al.* Expression of the largest CD97 and EMR2 isoforms on leukocytes facilitates a specific interaction with chondroitin sulfate on B cells. *J. Leukoc. Biol.* **77,** 112–119 (2005).
20. Karpus, O. N. *et al.* Shear stress-dependent downregulation of the adhesion-G protein-coupled receptor CD97 on circulating leukocytes upon contact with its ligand CD55. *J. Immunol.* **190,** 3740–3748 (2013).
21. Revenfeld, A. L. S. *et al.* Presence of HLA-DR molecules and HLA-DRB1 mRNA in circulating CD4(+) T cells. *Scand. J Immunol.* **84,** 211–221; 10.1111/sji.12462 (2016).
22. Corrigan, C. J. & Kay, A. B. CD4 T-lymphocyte activation in acute severe asthma. Relationship to disease severity and atopic status. *Am Rev Respir Dis* **141,** 970–977; 10.1164/ajrccm/141.4_Pt_1 (1990).
23. Plumpton, E. L. *et al.* Activation status of airway immune cells is a defining feature of severe asthma, regardless of fungal sensitisation. *Mucosal Immunol* **19,** 1587–1598; 10.1016/j.mucimm.2025.11.004 (2026).
24. Tippalagama, R. *et al.* HLA-DR marks recently divided antigen-specific effector CD4 T Cells in active tuberculosis patients. *J Immunol* **207,** 523–533; 10.4049/jimmunol.2100011 (2021).

25. Muhlethaler-Mottet, A., Otten, L. A., Steimle, V. & Mach, B. Expression of MHC class II molecules in different cellular and functional compartments is controlled by differential usage of multiple promoters of the transactivator CIITA. *EMBO J* **16,** 2851–2860; 10.1093/emboj/16.10.2851 (1997).
26. Schooten, E., Klous, P., van Den Elsen, Peter J & Holling, T. M. Lack of MHC-II expression in activated mouse T cells correlates with DNA methylation at the CIITA-PIII region. *Immunogenetics* **57,** 795–799; 10.1007/s00251-005-0051-8 (2005).
27. Tumes, D. J. *et al.* Epigenetic regulation of T-helper cell differentiation, memory, and plasticity in allergic asthma. *Immunol Rev* **278,** 8–19; 10.1111/imr.12560 (2017).
28. Sharma, S., Yang, I. V. & Schwartz, D. A. Epigenetic regulation of immune function in asthma. *J. Allergy Clin. Immunol.* **150,** 259–265; 10.1016/j.jaci.2022.06.002 (2022).
29. Alladina, J. *et al.* A human model of asthma exacerbation reveals transcriptional programs and cell circuits specific to allergic asthma. *Sci. Immunol.* **8,** eabq6352; 10.1126/sciimmunol.abq6352 (2023).
30. Hoffmann, F. *et al.* Origin, localization, and immunoregulatory properties of pulmonary phagocytes in allergic asthma. *Front. Immunol.* **7,** 107; 10.3389/fimmu.2016.00107 (2016).
31. Gueders, M. M. *et al.* Mouse models of asthma: a comparison between C57BL/6 and BALB/c strains regarding bronchial responsiveness, inflammation, and cytokine production. *Inflamm. Res* **58,** 845–854 (2009).

Answers to the reviewers' comments on the manuscript:

"CD97/ADGRE5 attenuates the induction of adaptive type 2 immune responses in allergic asthma"

We would like to thank the reviewers once again for the time and effort they have invested in reviewing our work. We have addressed all the points raised by Reviewer 3, provided additional data and revised the manuscript accordingly.

REVIEWER COMMENTS

Reviewer #1 (Remarks to the Author): Concerns were adequately addressed
Reviewer #2 (Remarks to the Author): The authors have addressed the concerns of this reviewer.

Reviewer #3 (Remarks to the Author):

The authors have addressed all comments in detail. The introduction, materials and methods, results, and discussion have been revised. However, the manuscript still remains highly descriptive. The data regarding the role of CD97 on DCs have been further strengthened. From my perspective, the following questions nevertheless remain open:

1. Is the primary regulatory mechanism mediated through DCs?

Yes, our data support the conclusion that the primary regulatory mechanism is mediated by DCs. This is caused by an intrinsic functional difference between WT and *Cd97*$^{-/-}$ DCs. We found increased frequencies of (activated) DC subsets (moDCs, partly cDC2s) in lung and mediastinal lymph nodes (mLNs) that drive type 2 immune responses (Fig. 4, Fig. S6). Furthermore, we demonstrated that BMDCs from WT and *Cd97*$^{-/-}$ differ functionally both *in vitro* (Fig. 5) and ultimately *in vivo* (Fig. 6). Nevertheless, as mentioned in the Discussion, we cannot completely rule out the possibility that CD97 also plays a role in CD4^{+} T cells independently of DCs.
Notably, WT and *Cd97*$^{-/-}$ allergen-pulsed BMDCs differ in the expression of more than 150 genes (new Supplementary Table 1, Supplementary Fig. 7) whereby a gene set is highly enriched in UniProt and Gene Ontology (GO) terms "signal", "extracellular region", "secreted", and "extracellular space". These transcriptional differences may explain the altered DC functions we observed and thereby support a primary role for DCs in mediating the effects of CD97.

What role does CD97 play on other immune cells? How is the receptor expressed under homeostatic versus inflammatory conditions?

It is unclear whether the reviewer is asking this generally or specifically about allergic asthma, so we will address both aspects.
CD97 is expressed on all circulating and many, but not all, tissue-resident immune cells in both humans and mice, although at varying levels (www.proteinatlas.org). In asthma, DCs, T cells, B cells, innate lymphoid cells (ILCs), eosinophils/neutrophils, and mast cells, are relevant and express CD97. Under homeostatic conditions, DCs showed the highest CD97 expression among lung leukocyte subsets, with eosinophils displaying the next highest levels, followed by neutrophils and lymphocytes, whereas alveolar macrophages were CD97-negative (Supplementary Fig. S2d).
In our study, we discussed a potential, yet unresolved role of CD97 in the DC–T cell immunological synapse, where CD97 is present on both cell types. The number of lung ILCs was similar in untreated and allergen-challenged WT and *Cd97*$^{-/-}$ mice, suggesting that CD97 on these cells is not primarily involved. Likewise, the expression of CD97 on eosinophils and neutrophils is unlikely to play a key role in allergic asthma. This is because the treatment of WT mice with a CD97 antibody during allergen challenge (see Supplementary Fig. 5g–l), when granulocytes infiltrate the lung, did not affect disease severity (see first paragraph in the Discussion).

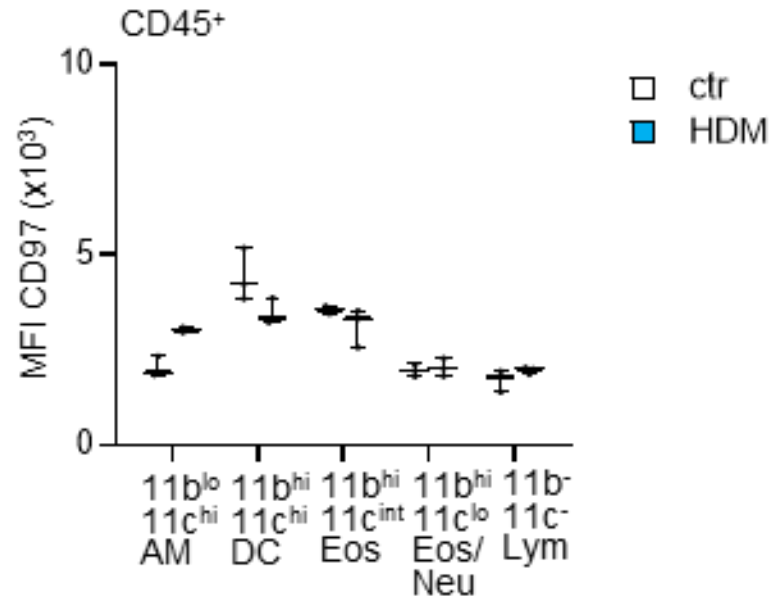

Fig. 1. CD97 expression on lung leukocytes under inflammatory conditions. CD97 expression was quantified by flow cytometry in lung leukocyte subsets from untreated and HDM-treated WT mice at the end of the acute HDM model. For the gating strategy, leukocyte subtyping based on CD11b/CD11c expression, and subset definitions, see Supplementary Fig. 2c; n=3 mice/group.

In the allergic asthma model, CD97 expression on lung leukocyte subsets under inflammatory conditions (at the study endpoint) was assessed in only three mice per group (see figure above). We were unable to increase the group size, as repeating the long-term asthma model solely for this purpose was not permitted under the approved ethical regulations.

What is known for CD97 in general under inflammatory conditions in human? In the pan-disease whole blood profile (www.proteinatlas.org) an enhanced *ADGRE5* expression is only observed in many, although not all, infectious diseases. Furthermore, in a study on patients with non-infectious systemic inflammatory response syndrome (SIRS) and sepsis, CD97 expression on blood neutrophils remained unchanged compared with healthy controls [1]. CD97 protein has been detected in various immune cells within inflammatory tissue samples from patients with chronic eczematous dermatitis [2], psoriasis[3], and multiple sclerosis [4]. Overall, our understanding of CD97 expression across leukocyte subsets under inflammatory conditions remains incomplete and warrants further study.

2. Liu et al. (DOI: 10.1126/science.abi5965) describe CD97 as a regulator required for maintaining splenic homeostasis, with receptor deficiency leading to impaired antibacterial responses. How do the authors interpret these findings in the context of their own work?

As discussed in our study, the mechanism described by Liu *et al.* [5] appears to be specific to splenic cDC2s, which reside in splenic bridging channels and directly contact circulating CD55-expressing red blood cells. CD97–CD55 interaction induces shedding of the CD97 N-terminal fragment, releasing the tethered internal agonist and activating Gα13–Rho GTPase signaling. This promotes actin remodeling and altered migration, ultimately retaining cDC2s within the spleen [5–7]. Accordingly, splenic cDC2 numbers were reduced in naïve *Adgre5*$^{-/-}$ mice [5], which we confirmed in our *Cd97*$^{-/-}$ mice (Fig. S6e).

This spleen-specific mechanism is unlikely to operate in the lung or mLNs, which differ in microanatomy and lack direct DC–red blood cell contact. Consistently, WT and *Cd97*$^{-/-}$ lung and mLN DCs of naïve animals are not expected to differ in activation status or actin content, which is indeed the case (e.g., Fig. 4; Suppl Fig S6). Moreover, the numbers and frequencies of total and activated cDC2s and moDCs in lungs and mLNs were similar in naïve mice (Fig. 4, S6). Likewise, peripheral LN cDC2s, as well as BM- and pre-splenic DC frequencies were comparable between WT and *Adgre5*$^{-/-}$ mice at baseline, as reported [5].

3. Why does differential regulation of DCs occur in vitro, even though an adhesion receptor is differentially regulated?

The reviewer may question how a cell surface receptor such as CD97, an adhesion GPCR, can regulate DC function. Indeed, adhesion receptors including E-cadherin and integrins are well-established regulators of DC migration, activation, and T-cell interactions [8,9].
The term "*adhesion GPCR*" reflects their large extracellular domains that mediate interactions with other cells or the extracellular matrix, as is the case for CD97. However, adhesion represents only one facet of adhesion GPCR functions (detailed review [10]). Receptor activation also alters the physiology of metabolically active cells such as adipocytes, hepatocytes, pancreatic islet cells, and myocytes [10]. CD97 has been linked *in vitro* to glycolytic metabolism [11] and apoptosis [12], where adhesion is unlikely to play a direct role. Accordingly, our data showing CD97-dependent changes in DC function *in vitro* support a broader regulatory role for adhesion GPCRs beyond adhesion.

References

1. Lewis, S. M. *et al.* Expression of CD11c and EMR2 on neutrophils: potential diagnostic biomarkers for sepsis and systemic inflammation. *Clin. Exp. Immunol.* **182,** 184–194; 10.1111/cei.12679 (2015).
2. Gray, J. X. *et al.* CD97 is a processed, seven-transmembrane, heterodimeric receptor associated with inflammation. *J. Immunol.* **157,** 5438–5447 (1996).
3. Wandel, E., Saalbach, A., Sittig, D., Gebhardt, C. & Aust, G. Thy-1 (CD90) is an interaction partner for CD97 on activated endothelial cells. *J. Immunol.* **188,** 1442–1450 (2012).
4. Visser, L. *et al.* Expression of the EGF-TM7 receptor CD97 and its ligand CD55 (DAF) in multiple sclerosis brain lession. *J. Neuroimmunol.* **132,** 156–163 (2002).
5. Liu, D. *et al.* CD97 promotes spleen dendritic cell homeostasis through the mechanosensing of red blood cells. *Science* **375,** eabi5965; 10.1126/science.abi5965 (2022).
6. Ward, Y. *et al.* LPA receptor heterodimerizes with CD97 to amplify LPA-initiated RHO-dependent signaling and invasion in prostate cancer cells. *Cancer Res.* **71,** 7301–7311 (2011).
7. Hilbig, D. *et al.* Mechano-dependent phosphorylation of the PDZ-binding motif of CD97/ADGRE5 modulates cellular detachment. *Cell Rep.* **24,** 1986–1995 (2018).
8. Schittenhelm, L., Hilkens, C. M. & Morrison, V. L. β(2) Integrins as regulators of dendritic cell, monocyte, and macrophage function. *Front Immunol.* **8,** 1866; 10.3389/fimmu.2017.01866 (2017).
9. Jiang, A. *et al.* Disruption of E-cadherin-mediated adhesion induces a functionally distinct pathway of dendritic cell maturation. *Immunity* **27,** 610–624; 10.1016/j.immuni.2007.08.015 (2007).
10. Langenhan, T. *et al.* Adhesion G protein-coupled receptors. *Pharmacol Rev.* **78,** 100116; 10.1016/j.pharmr.2026.100116 (2026).
11. Ravn-Boess, N. *et al.* The expression profile and tumorigenic mechanisms of CD97 (ADGRE5) in glioblastoma render it a targetable vulnerability. *Cell Rep.* **42,** 113374; 10.1016/j.celrep.2023.113374 (2023).
12. Hsiao, C. C. *et al.* The Adhesion GPCR CD97 inhibits apoptosis. *Int. J Biochem. Cell Biol.* **65,** 197–208 (2015).
