# Supplementary material for: CD97/ADGRE5 attenuates the induction of adaptive type 2 immune responses in allergic asthma
Source: Nat Commun. 2026 Aug 28;17:9226. doi: 10.1038/s41467-026-76948-9 (PMC13522487; doi:10.1038/s41467-026-76948-9)
Supplement: Supplementary file 5 [file 41467_2026_76948_MOESM3_ESM.docx]

**Supplementary Data File**

**Differentially expressed genes in CD11c^+^ BMDCs of WT and *Cd97^-/-^* mice**

| gene | mean WT | mean *Cd97^-/-^* | log2 FoldChange (lfc) | lfcSE | stat | p-value | p-adj |
| --- | --- | --- | --- | --- | --- | --- | --- |
| Adgre5 | 3126.1 | 246.6 | -3.7 | 0.2 | -22.4 | 4.83E-111 | 1.15E-106 |
| Gm16867 | 43.3 | 1073.6 | 4.6 | 0.4 | 13.2 | 1.56E-39 | 1.86E-35 |
| Dynlt1b | 117.6 | 1219.3 | 3.4 | 0.3 | 11.6 | 6.09E-31 | 4.83E-27 |
| Cacna1a | 13.9 | 618.4 | 5.6 | 0.5 | 10.3 | 5.44E-25 | 3.24E-21 |
| Gm38378 | 0.0 | 197.6 | 10.0 | 1.1 | 9.4 | 4.06E-21 | 1.93E-17 |
| Gm37094 | 110.5 | 504.9 | 2.2 | 0.2 | 9.3 | 1.52E-20 | 6.04E-17 |
| ENSMUSG00000121395 | 10.0 | 221.9 | 4.5 | 0.5 | 8.3 | 7.65E-17 | 2.18E-13 |
| ENSMUSG00000121430 | 662.3 | 1444.1 | 1.1 | 0.1 | 8.3 | 8.25E-17 | 2.18E-13 |
| Inpp4b | 8.5 | 160.5 | 5.0 | 0.6 | 8.4 | 6.77E-17 | 2.18E-13 |
| Chil3 | 383.7 | 1274.8 | 1.7 | 0.2 | 8.0 | 1.13E-15 | 2.70E-12 |
| Gm54588 | 1910.7 | 0.0 | -26.9 | 3.4 | -8.0 | 1.74E-15 | 3.77E-12 |
| Lama5 | 759.2 | 1878.3 | 1.3 | 0.2 | 7.9 | 2.85E-15 | 5.65E-12 |
| Gm14327 | 0.0 | 127.9 | 9.4 | 1.2 | 7.8 | 8.43E-15 | 1.54E-11 |
| Gm37847 | 24.4 | 164.9 | 2.8 | 0.4 | 7.6 | 2.03E-14 | 3.44E-11 |
| Fn1 | 21939.0 | 44733.7 | 1.0 | 0.1 | 7.6 | 2.65E-14 | 4.21E-11 |
| D8Ertd738e | 2026.2 | 37.1 | -5.8 | 0.8 | -7.4 | 1.01E-13 | 1.50E-10 |
| Dynlt1a | 202.4 | 456.6 | 1.2 | 0.2 | 7.4 | 1.23E-13 | 1.72E-10 |
| Gdf15 | 314.8 | 77.4 | -2.0 | 0.3 | -7.4 | 1.65E-13 | 2.19E-10 |
| B3gnt3 | 1473.3 | 710.1 | -1.1 | 0.1 | -7.4 | 1.85E-13 | 2.32E-10 |
| Tmem254 | 339.0 | 787.3 | 1.2 | 0.2 | 7.3 | 3.35E-13 | 3.99E-10 |
| Tusc3 | 861.4 | 1582.6 | 0.9 | 0.1 | 7.2 | 6.35E-13 | 7.19E-10 |
| Man2b1 | 5629.6 | 14198.2 | 1.3 | 0.2 | 7.0 | 1.91E-12 | 2.07E-09 |
| Tmem158 | 240.6 | 557.6 | 1.2 | 0.2 | 7.0 | 2.20E-12 | 2.28E-09 |
| Plxnb2 | 1296.2 | 2894.4 | 1.2 | 0.2 | 6.8 | 9.08E-12 | 9.00E-09 |
| Prag1 | 809.6 | 233.2 | -1.8 | 0.3 | -6.8 | 1.00E-11 | 9.55E-09 |
| Dynlt1-ps1 | 171.5 | 332.0 | 1.0 | 0.1 | 6.8 | 1.33E-11 | 1.22E-08 |
| Gm20634 | 98.3 | 0.0 | -22.7 | 3.4 | -6.7 | 1.93E-11 | 1.70E-08 |
| Igfbp4 | 8160.3 | 13877.2 | 0.8 | 0.1 | 6.3 | 3.52E-10 | 2.99E-07 |
| Ocel1 | 794.7 | 1512.0 | 0.9 | 0.2 | 6.0 | 1.51E-09 | 1.24E-06 |
| Ctr9 | 1046.3 | 590.7 | -0.8 | 0.1 | -6.0 | 1.79E-09 | 1.40E-06 |
| Rab4a | 81.1 | 10.0 | -3.0 | 0.5 | -6.0 | 1.83E-09 | 1.40E-06 |
| Prdx2 | 10138.7 | 7035.1 | -0.5 | 0.1 | -6.0 | 1.90E-09 | 1.41E-06 |
| Il1b | 14233.9 | 23033.5 | 0.7 | 0.1 | 6.0 | 2.66E-09 | 1.86E-06 |
| Loxl2 | 38.0 | 154.2 | 2.0 | 0.3 | 6.0 | 2.60E-09 | 1.86E-06 |
| Gm16183 | 0.0 | 29.1 | 7.2 | 1.2 | 5.9 | 2.74E-09 | 1.86E-06 |
| Orm1 | 193.0 | 29.4 | -2.7 | 0.5 | -5.9 | 4.48E-09 | 2.96E-06 |
| Rnf150 | 339.1 | 108.8 | -1.6 | 0.3 | -5.9 | 4.61E-09 | 2.97E-06 |
| Slc16a7 | 15.9 | 88.2 | 2.4 | 0.4 | 5.8 | 6.33E-09 | 3.97E-06 |
| Gm27177 | 10.1 | 68.1 | 2.7 | 0.5 | 5.8 | 7.57E-09 | 4.62E-06 |
| Igf2bp2 | 1637.8 | 859.0 | -0.9 | 0.2 | -5.8 | 8.02E-09 | 4.77E-06 |
| Gm35546 | 34.4 | 2.4 | -3.8 | 0.7 | -5.8 | 8.60E-09 | 4.90E-06 |
| Slc5a3 | 841.0 | 1238.9 | 0.6 | 0.1 | 5.8 | 8.64E-09 | 4.90E-06 |
| Timp2 | 336.7 | 532.5 | 0.7 | 0.1 | 5.7 | 1.02E-08 | 5.64E-06 |
| Car4 | 1346.6 | 4236.8 | 1.7 | 0.3 | 5.7 | 1.31E-08 | 7.08E-06 |
| Gbp2b | 643.2 | 389.8 | -0.7 | 0.1 | -5.7 | 1.47E-08 | 7.77E-06 |
| Gm3365 | 28.4 | 0.3 | -6.6 | 1.2 | -5.6 | 1.64E-08 | 8.48E-06 |
| Fam32a | 4116.2 | 6882.0 | 0.7 | 0.1 | 5.6 | 1.76E-08 | 8.93E-06 |
| Gm14418 | 91.8 | 199.6 | 1.1 | 0.2 | 5.6 | 2.51E-08 | 1.24E-05 |
| Cd14 | 10492.8 | 15922.1 | 0.6 | 0.1 | 5.5 | 3.16E-08 | 1.54E-05 |
| Ankhd1 | 702.8 | 300.9 | -1.2 | 0.2 | -5.5 | 3.54E-08 | 1.65E-05 |
| Gm4841 | 70.7 | 14.0 | -2.3 | 0.4 | -5.5 | 3.50E-08 | 1.65E-05 |
| Mtus1 | 1372.3 | 757.1 | -0.9 | 0.2 | -5.5 | 3.83E-08 | 1.75E-05 |
| Sdc1 | 8189.6 | 2468.5 | -1.7 | 0.3 | -5.5 | 4.11E-08 | 1.85E-05 |
| Gm35572 | 1.8 | 40.0 | 4.5 | 0.8 | 5.5 | 4.24E-08 | 1.87E-05 |
| Il12rb1 | 293.0 | 138.0 | -1.1 | 0.2 | -5.5 | 4.69E-08 | 2.03E-05 |
| Ccdc80 | 496.8 | 1000.2 | 1.0 | 0.2 | 5.4 | 7.75E-08 | 3.29E-05 |
| Dach1 | 453.3 | 714.2 | 0.7 | 0.1 | 5.4 | 8.42E-08 | 3.52E-05 |
| Mri1 | 116.5 | 28.1 | -2.0 | 0.4 | -5.4 | 8.71E-08 | 3.52E-05 |
| Snx15 | 693.9 | 1002.0 | 0.5 | 0.1 | 5.4 | 8.74E-08 | 3.52E-05 |
| Gm4631 | 357.6 | 599.0 | 0.7 | 0.1 | 5.3 | 1.08E-07 | 4.29E-05 |
| AW046200 | 222.4 | 94.1 | -1.2 | 0.2 | -5.3 | 1.28E-07 | 4.92E-05 |
| Gm9725 | 149.7 | 56.4 | -1.4 | 0.3 | -5.3 | 1.28E-07 | 4.92E-05 |
| Arhgef3 | 790.7 | 1366.3 | 0.8 | 0.1 | 5.3 | 1.41E-07 | 5.33E-05 |
| AI506816 | 385.7 | 566.1 | 0.6 | 0.1 | 5.2 | 1.57E-07 | 5.74E-05 |
| Pacsin3 | 12.7 | 56.9 | 2.2 | 0.4 | 5.2 | 1.56E-07 | 5.74E-05 |
| Gm14296 | 617.2 | 1060.1 | 0.8 | 0.1 | 5.2 | 1.69E-07 | 6.10E-05 |
| Gm14393 | 0.0 | 16.8 | 6.5 | 1.2 | 5.2 | 2.04E-07 | 7.23E-05 |
| Klhl26 | 682.6 | 1044.2 | 0.6 | 0.1 | 5.2 | 2.09E-07 | 7.32E-05 |
| Lrrc25 | 4267.2 | 2666.9 | -0.7 | 0.1 | -5.2 | 2.55E-07 | 8.80E-05 |
| Eps15l1 | 1261.7 | 453.7 | -1.5 | 0.3 | -5.1 | 3.21E-07 | 1.09E-04 |
| 2500002B13Rik | 125.9 | 302.3 | 1.3 | 0.2 | 5.1 | 3.36E-07 | 1.12E-04 |
| Hook2 | 1038.9 | 653.8 | -0.7 | 0.1 | -5.1 | 3.38E-07 | 1.12E-04 |
| Slpi | 5680.5 | 8589.5 | 0.6 | 0.1 | 5.1 | 3.67E-07 | 1.20E-04 |
| Adamts7 | 116.9 | 234.6 | 1.0 | 0.2 | 5.1 | 4.33E-07 | 1.39E-04 |
| Cilp2 | 2.0 | 41.5 | 4.3 | 0.9 | 5.0 | 4.87E-07 | 1.55E-04 |
| Gm10654 | 34.0 | 1.2 | -4.8 | 1.0 | -5.0 | 6.94E-07 | 2.17E-04 |
| Dynlt1c | 437.7 | 802.4 | 0.9 | 0.2 | 5.0 | 7.27E-07 | 2.25E-04 |
| Efcab7 | 137.8 | 57.8 | -1.3 | 0.3 | -4.9 | 7.74E-07 | 2.36E-04 |
| Nufip1 | 372.1 | 743.0 | 1.0 | 0.2 | 4.9 | 8.39E-07 | 2.53E-04 |
| Hgfac | 279.9 | 541.2 | 1.0 | 0.2 | 4.9 | 8.54E-07 | 2.54E-04 |
| Pnpla7 | 884.4 | 1601.7 | 0.9 | 0.2 | 4.9 | 1.11E-06 | 3.25E-04 |
| Maged1 | 3718.9 | 2564.2 | -0.5 | 0.1 | -4.9 | 1.15E-06 | 3.35E-04 |
| Mbnl1 | 6602.2 | 4816.8 | -0.5 | 0.1 | -4.8 | 1.47E-06 | 4.20E-04 |
| Zfp469 | 865.7 | 553.8 | -0.6 | 0.1 | -4.8 | 1.51E-06 | 4.27E-04 |
| Fabp7 | 193.0 | 396.4 | 1.0 | 0.2 | 4.8 | 1.76E-06 | 4.91E-04 |
| Mid1-ps1xf3 | 3.6 | 26.3 | 2.9 | 0.6 | 4.8 | 1.79E-06 | 4.91E-04 |
| Timp1 | 1334.1 | 2459.3 | 0.9 | 0.2 | 4.8 | 1.79E-06 | 4.91E-04 |
| Rnf157 | 176.8 | 605.2 | 1.8 | 0.4 | 4.8 | 1.91E-06 | 5.17E-04 |
| Arpin | 952.4 | 613.0 | -0.6 | 0.1 | -4.8 | 1.97E-06 | 5.27E-04 |
| F11r | 319.0 | 520.3 | 0.7 | 0.1 | 4.7 | 2.29E-06 | 5.98E-04 |
| Micu3 | 251.7 | 131.2 | -0.9 | 0.2 | -4.7 | 2.27E-06 | 5.98E-04 |
| Fam120b | 608.7 | 1329.1 | 1.1 | 0.2 | 4.7 | 2.72E-06 | 7.04E-04 |
| Adam39 | 13.4 | 0.0 | -6.2 | 1.3 | -4.7 | 2.79E-06 | 7.05E-04 |
| Cc2d1a | 1103.0 | 1834.7 | 0.7 | 0.2 | 4.7 | 2.78E-06 | 7.05E-04 |
| Gbp4 | 3546.7 | 1879.4 | -0.9 | 0.2 | -4.7 | 3.30E-06 | 8.26E-04 |
| Nts | 310.7 | 568.3 | 0.9 | 0.2 | 4.6 | 3.35E-06 | 8.31E-04 |
| Spp1 | 9206.3 | 16875.0 | 0.9 | 0.2 | 4.6 | 3.41E-06 | 8.38E-04 |
| Cish | 506.6 | 1121.2 | 1.1 | 0.2 | 4.6 | 3.48E-06 | 8.45E-04 |
| Pecam1 | 350.1 | 572.9 | 0.7 | 0.2 | 4.6 | 3.57E-06 | 8.51E-04 |
| Socs1 | 4510.4 | 3555.1 | -0.3 | 0.1 | -4.6 | 3.58E-06 | 8.51E-04 |
| Spred3 | 37.8 | 177.2 | 2.2 | 0.5 | 4.6 | 4.22E-06 | 9.94E-04 |
| Zfp709 | 659.3 | 414.2 | -0.7 | 0.1 | -4.6 | 4.26E-06 | 9.94E-04 |
| Ankrd37 | 2275.5 | 1472.1 | -0.6 | 0.1 | -4.6 | 4.89E-06 | 1.13E-03 |
| Zfp991 | 469.7 | 274.0 | -0.8 | 0.2 | -4.6 | 4.98E-06 | 1.14E-03 |
| Ermap | 0.0 | 15.5 | 6.3 | 1.4 | 4.6 | 5.07E-06 | 1.15E-03 |
| Il18bp | 4159.3 | 2013.9 | -1.0 | 0.2 | -4.6 | 5.31E-06 | 1.19E-03 |
| Gm9843 | 764.8 | 304.7 | -1.3 | 0.3 | -4.5 | 5.57E-06 | 1.24E-03 |
| Gm15784 | 19.9 | 79.0 | 2.0 | 0.4 | 4.5 | 6.91E-06 | 1.52E-03 |
| Esam | 476.2 | 259.0 | -0.9 | 0.2 | -4.5 | 7.75E-06 | 1.69E-03 |
| Asrgl1 | 416.5 | 652.5 | 0.6 | 0.1 | 4.4 | 9.81E-06 | 2.12E-03 |
| Gm14540 | 69.0 | 15.2 | -2.2 | 0.5 | -4.4 | 1.02E-05 | 2.18E-03 |
| Zfp791 | 2.5 | 215.7 | 6.4 | 1.5 | 4.4 | 1.09E-05 | 2.31E-03 |
| 2610203C22Rik | 13.2 | 56.8 | 2.1 | 0.5 | 4.4 | 1.15E-05 | 2.42E-03 |
| Btbd2 | 984.1 | 641.2 | -0.6 | 0.1 | -4.4 | 1.32E-05 | 2.73E-03 |
| Tnfrsf21 | 3513.7 | 5461.9 | 0.6 | 0.1 | 4.4 | 1.31E-05 | 2.73E-03 |
| Gm43864 | 0.0 | 11.6 | 5.9 | 1.4 | 4.3 | 1.38E-05 | 2.84E-03 |
| Selenop | 426.9 | 703.7 | 0.7 | 0.2 | 4.3 | 1.42E-05 | 2.89E-03 |
| Il12rb2 | 221.7 | 419.3 | 0.9 | 0.2 | 4.3 | 1.50E-05 | 3.02E-03 |
| Capn5 | 1041.8 | 1389.1 | 0.4 | 0.1 | 4.3 | 1.52E-05 | 3.03E-03 |
| A230103J11Rik | 37.6 | 113.2 | 1.6 | 0.4 | 4.3 | 1.53E-05 | 3.03E-03 |
| Atp8b1 | 0.8 | 22.6 | 4.9 | 1.1 | 4.3 | 1.66E-05 | 3.26E-03 |
| AB124611 | 537.1 | 774.5 | 0.5 | 0.1 | 4.3 | 1.87E-05 | 3.65E-03 |
| Ctcf | 992.3 | 646.0 | -0.6 | 0.1 | -4.2 | 2.16E-05 | 4.08E-03 |
| Mcfd2 | 2200.3 | 3139.9 | 0.5 | 0.1 | 4.2 | 2.15E-05 | 4.08E-03 |
| Tuba8 | 194.2 | 340.2 | 0.8 | 0.2 | 4.2 | 2.16E-05 | 4.08E-03 |
| Usp25 | 8580.1 | 6120.6 | -0.5 | 0.1 | -4.2 | 2.14E-05 | 4.08E-03 |
| Gm11808 | 3251.5 | 5030.3 | 0.6 | 0.1 | 4.2 | 2.23E-05 | 4.18E-03 |
| Gm14305 | 193.0 | 368.4 | 0.9 | 0.2 | 4.2 | 2.52E-05 | 4.69E-03 |
| Foxm1 | 25.8 | 71.0 | 1.4 | 0.3 | 4.2 | 2.69E-05 | 4.96E-03 |
| Mid1-ps1xf2 | 5.7 | 34.5 | 2.6 | 0.6 | 4.2 | 2.71E-05 | 4.96E-03 |
| Dgat2 | 611.9 | 896.3 | 0.6 | 0.1 | 4.2 | 3.00E-05 | 5.45E-03 |
| Mab21l3 | 750.5 | 452.4 | -0.7 | 0.2 | -4.1 | 3.44E-05 | 6.20E-03 |
| Gm10804 | 12.5 | 87.7 | 2.8 | 0.7 | 4.1 | 3.81E-05 | 6.81E-03 |
| Lhfpl2 | 1680.9 | 3377.8 | 1.0 | 0.2 | 4.1 | 3.85E-05 | 6.83E-03 |
| Gbp7 | 6423.5 | 4825.5 | -0.4 | 0.1 | -4.1 | 3.92E-05 | 6.91E-03 |
| Phrf1 | 1649.8 | 1100.7 | -0.6 | 0.1 | -4.1 | 3.98E-05 | 6.96E-03 |
| Cyp4f18 | 817.2 | 429.5 | -0.9 | 0.2 | -4.1 | 4.01E-05 | 6.96E-03 |
| Pcmt1 | 1893.0 | 1264.0 | -0.6 | 0.1 | -4.1 | 4.08E-05 | 7.03E-03 |
| Hid1 | 63.1 | 136.5 | 1.1 | 0.3 | 4.1 | 4.20E-05 | 7.19E-03 |
| Gm24644 | 28.8 | 4.3 | -2.7 | 0.7 | -4.1 | 4.37E-05 | 7.42E-03 |
| Adgre1 | 9812.7 | 23518.2 | 1.3 | 0.3 | 4.1 | 4.49E-05 | 7.57E-03 |
| Rbl1 | 1507.0 | 1071.6 | -0.5 | 0.1 | -4.1 | 4.62E-05 | 7.74E-03 |
| Adam12 | 154.3 | 70.1 | -1.1 | 0.3 | -4.1 | 4.74E-05 | 7.89E-03 |
| Gm47092 | 59.0 | 118.0 | 1.0 | 0.2 | 4.1 | 4.78E-05 | 7.90E-03 |
| Aldh3b1 | 2061.9 | 3598.2 | 0.8 | 0.2 | 4.0 | 5.21E-05 | 8.54E-03 |
| Ifitm6 | 96.1 | 202.7 | 1.1 | 0.3 | 4.0 | 5.31E-05 | 8.64E-03 |
| mt-Nd2 | 3229.0 | 7079.1 | 1.1 | 0.3 | 4.0 | 5.34E-05 | 8.64E-03 |
| Gm56867 | 51.3 | 110.4 | 1.1 | 0.3 | 4.0 | 5.56E-05 | 8.92E-03 |
| Kdm3a | 2263.3 | 1738.1 | -0.4 | 0.1 | -4.0 | 5.58E-05 | 8.92E-03 |
| Gm26581 | 90.1 | 178.0 | 1.0 | 0.2 | 4.0 | 5.85E-05 | 9.28E-03 |
| Gm45694 | 29.6 | 2.7 | -3.5 | 0.9 | -4.0 | 6.06E-05 | 9.55E-03 |
| Uba52 | 8464.1 | 12023.0 | 0.5 | 0.1 | 4.0 | 6.13E-05 | 9.60E-03 |
| Gm9484 | 123.6 | 70.6 | -0.8 | 0.2 | -4.0 | 6.40E-05 | 9.95E-03 |

Genes differentially expressed between CD11c^+^ OVA-pulsed BMDCs of WT and *Cd97^-/-^* mice (n=4 each); DESeq2 analysis. The list of differentially expressed genes was filtered according to Benjamini-Hochberg adjusted p-values (padj) with a cut off below 0.01. mean WT: average normalized count across WT samples, log2 FoldChange (lfc, *Cd97^-/-^* versus WT), lfcSE: standard error of lfc, stat: Wald statistic (2-sided), p-value: unadjusted p-value.
